# RNase1-driven ALK-activation is an oncogenic driver and therapeutic target in non-small cell lung cancer

**DOI:** 10.1038/s41392-025-02206-x

**Published:** 2025-04-18

**Authors:** Zhengyu Zha, Chunxiao Liu, Meisi Yan, Cong Chen, Cheng Yu, Yaohui Chen, Chenhao Zhou, Lu Li, Yi-Chuan Li, Hiro Yamaguchi, Leiguang Ye, Tong Liu, Ying-Nai Wang, Heng-Huan Lee, Wen-Hao Yang, Li-Chuan Chan, Baozhen Ke, Jennifer L. Hsu, Lieming Ding, Dong Ji, Peng Pan, Yiran Meng, Yue Pu, Lunxu Liu, Mien-Chie Hung

**Affiliations:** 1 Department of Thoracic Surgery and Institute of Thoracic Oncology, West China Hospital, University of Sichuan, Chengdu, Sichuan China; 2 Institute of Precision Medicine, The First Affiliated Hospital, Sun Yat-sen University, Guangzhou, Guangdong China; 3 Department of Pathology, School of Basic Medical Sciences, Harbin Medical University, Harbin, Heilongjiang China; 4 Department of Molecular and Cellular Oncology, The University of Texas MD Anderson Cancer Center, Houston, TX USA; 5 Department of Liver Cancer and Transplant, Zhongshan Hospital, Fudan University and Key Laboratory of Carcinogenesis and Cancer Invasion, Ministry of Education, Shanghai, China; 6 Department of Medical Oncology, Cancer Center, West China Hospital, Sichuan University, Chengdu, Sichuan China; 7 Lung Cancer Center, West China Hospital, Sichuan University, Chengdu, Sichuan China; 8 Betta Pharmaceuticals Co. Ltd, Hangzhou, China; 9 Hangzhou Repugene Technology Co., Ltd, Hangzhou, China

**Keywords:** Lung cancer, Cancer therapy

## Abstract

Targeted therapy has achieved significant success in the treatment of non-small cell lung cancer (NSCLC), particularly in patients harboring common oncogenic driver mutations such as EGFR, KRAS, and ALK rearrangement. However, ~35–50% of NSCLC patients without tyrosine kinase mutation or rearrangement (non-mutated) cannot benefit from these targeted treatments, highlighting the urgent need for novel therapeutic strategies for this patient population. In this study, we report a non-canonical role of human secretory ribonuclease 1 (RNase1), which binds to and activates wild-type ALK in lung cancer cells, thereby triggering its downstream signaling pathway. RNase1-driven ALK-activation (RDAA) cells exhibit enhanced cell proliferation, migration, and colony formation. Additionally, RDAA facilitates tumor formation in fibroblast models, further underscoring its oncogenic potential in vivo. Importantly, RDAA lung cancer cells exhibit marked sensitivity to FDA-approved ALK inhibitors. Tumor growth suppression and survival were substantially improved in both RDAA-positive NSCLC cell line-derived and patient-derived xenograft tumor models treated with ALK inhibitors. Monoclonal antibodies against RNase1 and phosphorylated-ALK were used to analyze two different human NSCLC tissue cohorts by immunohistochemical staining identified 10.4% (5/48) and 8.5% (100/1173) patients who were RDAA positive, respectively. Notably, among the nine RDAA-positive NSCLC patients who accepted ALK inhibitor treatment, five achieved objective response including two who experienced complete response (CR). Together, the current study identifies RDAA as an oncogenic driver and proposes an effective targeted therapy strategy for non-mutated NSCLC patients.

## Introduction

Lung cancer remains the leading cause of cancer-related deaths worldwide, with the majority of cases being non-small cell lung cancer (NSCLC).^1^At present, targeted therapy has become one of the primary treatment strategies for lung cancer patients, particularly those with mutant EGFR, KRAS G12C, and other typical oncogenic drivers in NSCLC.^2–6^ For example, treatment of sensitive EGFR-mutated tumors with third-generation EGFR-TKIs (such as osimertinib) had achieved response rates of more than 65% (median progression-free survival of 9–12.4 months).^7^ Targeted therapy with sotorasib or adagrasib have shown significant improvements in survival outcomes for KRAS G12C-mutant cancer patients. In clinical trials, sotorasib demonstrated an objective response rate (ORR) of 28.1%, compared to 13.2% for standard chemotherapy (median overall survival of 9.2–19.2 months) and adagrasib achieved an ORR of 42.9%.^8,9^ However, there are 35–50% of NSCLC patients without tyrosine kinase mutation or rearrangement (non-mutated) do not benefit from current target therapy.^6,10^Therefore, exploring new oncogenic drivers and elucidating their mechanisms, along with developing corresponding targeted therapies, are crucial steps toward expanding the cohort of NSCLC patients who can benefit from such treatments and improving overall prognosis.

The ALK gene, which was initially identified in 1994 by Morris from anaplastic large cell lymphoma (ALCL), encodes a receptor tyrosine kinase (RTK) belonging to the highly conserved insulin receptor superfamily. The wild-type (WT) ALK protein is known to play a crucial role in the development and functional regulation of the nervous system.^11^ In normal physiological conditions, exogenous ligands, such as ALKAL proteins, bind to the N-terminal extracellular domain of ALK, inducing dimerization of the receptor. This dimerization leads to autophosphorylation of the kinase domain of ALK, thereby activating intracellular downstream signaling pathways that regulate processes such as cell proliferation, survival, and differentiation. In malignancies, however, structural fusions or rearrangements occur between the 3′ terminal of the ALK gene, which encodes the kinase domain, and other partner genes at the 5′ terminal. These rearrangements result in ligand-independent dimerization and sustained hyperactivation of ALK, which in turn drives pro-mitogenic and anti-apoptotic signaling pathways, thereby facilitating tumor progression.^12–14^ In NSCLC, structural fusions involving ALK gene rearrangements are of critical importance. To date, over 90 distinct fusion partners have been identified in non-small cell lung cancer (NSCLC), with Echinoderm microtubule-associated protein-like 4 (EML4)-ALK being the most common ALK fusion in NSCLC, accounting for approximately 85% of all ALK-positive NSCLC cases.^15^ Given the critical role of ALK rearrangements in NSCLC progression, treatment with ALK tyrosine kinase inhibitors (TKIs), such as crizotinib, has been shown to significantly improve the survival outcomes of patients with ALK rearrangements or fusions, achieving a response rate of more than 65% and a median progression-free survival of 7.7 to 10.9 months.^16–18^ Notably, the third-generation ALK-TKIs—lorlatinib, not only address the resistance issues commonly associated with the earlier generations of ALK inhibitors but also demonstrate a dramatic reduction in the risk of central nervous system (CNS) progression compared to crizotinib, thereby significantly enhances survival and prognosis for patients with ALK-positive tumors (PFS: lorlatinib VS crizotinib: not reached vs 9.3 months, HR for disease progression or death of 0.28).^4,19^ Although ALK rearrangements are recognized as significant oncogenic drivers and effective therapeutic targets in NSCLC,^17,18,20–25^ little is known about the role of WT ALK in cancer development.^26^ Due to the limitations of current diagnostic technologies and methods, ALK-TKIs can only benefit patients diagnosed with ALK rearrangement or fusion.

Recent studies have identified additional potential oncogenic drivers that may serve as novel therapeutic targets. Among these, several members of the secretory ribonucleases (RNase) family have been found to bind to specific receptor tyrosine kinases (RTKs) and continuously activate their downstream pro-tumor signaling pathways to drive tumor progression. For instance, RNase1 has been shown to interact with EphA4 in breast cancer, RNase5 with EGFR in pancreatic cancer, RNase4 with AXL in prostate cancer, RNase7 with ROS1 in liver cancer,^27–30^ respectively. Our previous research demonstrated that RNase1 can promote immune progression in hepatocellular carcinoma by activating ALK signaling pathway in macrophages, thereby reshaping the tumor immune microenvironment.^31^ However, whether RNase1 directly functions as a tumor-driving factor remains uncertain. In current study, we discovered RNase1-driven-ALK-activation (RDAA) as a novel oncogenic driver in NSCLC, elucidated its oncogenic mechanisms, and explored an effective targeted therapy strategy targeting RDAA through preclinical experiments and small clinical therapeutic trials.

Our findings provide new insights into the application of TKI therapies and potential for targeted therapeutic strategies for patients who lack known oncogenic drivers, which could pave the way for more personalized and effective treatment approaches, improving prognosis and outcomes in this challenging patient cohort. This research also highlights the potential for expanding treatment options for NSCLC patients, including those who are currently underrepresented in targeted therapy treatment regimens.

## Results

### RNase1 binds to and activates ALK as its ligand in lung cancer cells

**Fig. 1:**
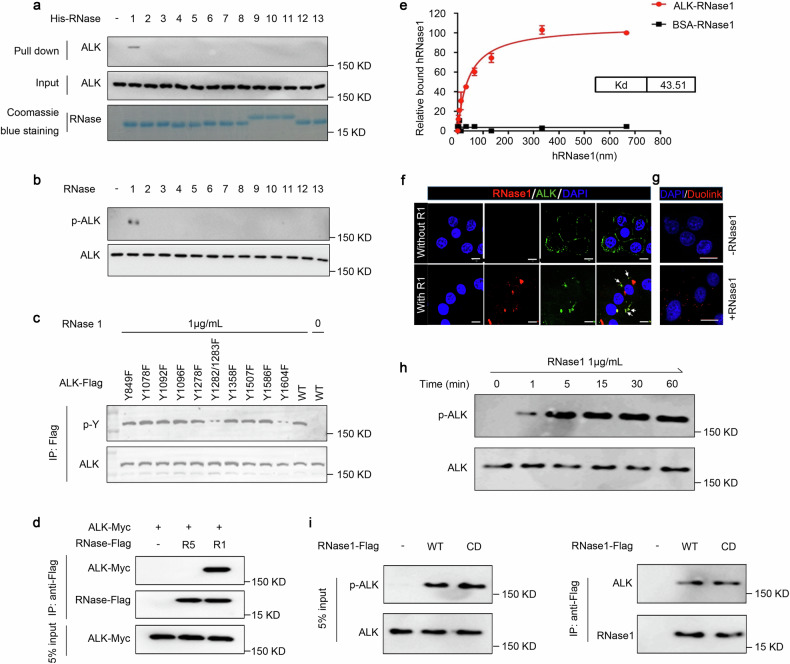
RNase1 binds to and activates ALK as its ligand in lung cancer cells. **a** Each of the 13 recombinantly purified 6´ N-terminal His-tagged RNases (10 µg) was incubated with H1299 lysate followed by Ni-His beads pull down and Western blot analysis with the indicated antibodies. **b** Each of the 13 RNases was added into the H1299 culture medium at a final concentration of 1 mg/ml. After 30 min incubation, cells were lysed and subjected to Western blotting with the indicated antibodies. A phospho-ALK antibody was used to detect ALK Y1604 phosphorylation. **c** Western blot analysis of ALK phosphorylation sites in HEK293 cells. Flag-tagged wild type or Tyr mutant ALK plasmids were transfected into HEK293 cells and then IP with Flag-beads. A pan-phosphorylated Tyr antibody (4G10) was used to detect ALK phosphorylation. **d** Plasmids expressing Myc-tagged ALK and C-terminal Flag-tagged RNase1 (R1) were transfected into HeLa cells. Cell lysates were harvested and subjected to co-immunoprecipitation (co-IP) assay. RNase1 was detected using the Flag antibody. RNase5 (R5) was used as a negative control. **e** In vitro binding affinity assay of ALK and RNase1. Kd, dissociation constant. BSA was used as a negative control. **f** Immunofluorescence microscopy of H1299 cells with or without RNase1 expression. Yellow dots and white arrows both indicate co-localization of ALK and RNase1. Scale bar, 20 mm. **g** H1299 cells with or without RNase1 expression were subjected to Duo Link assay. Red dots indicate binding between ALK and RNase1. Scale bar, 20 mm. **h** Time course analysis of ALK activation in H1299 cells by RNase1 (1 mg/ml). ALK phos-Y1604 was used as an indicator of ALK activation. **i** Co-IP of ALK and RNAse1 from HEK293T cells transfected with wild-type (WT) or catalytic-deficient (CD) RNase1-expressing plasmid followed by Western blotting with the indicated antibodies

To further investigate the relationship between ribonucleases and RTKs, we initially cloned and purified all 13 human secretory RNases from bacteria,^28^ and tested their interaction with multiple RTKs, including wild-type ALK. Interestingly, among the 13 ribonucleases, only RNase1 (R1), but not the others, bound to endogenous ALK in H1299 human lung cancer cells expressing WT ALK (Fig. 1a). Consistently, only RNase1, but not the others, induced ALK phosphorylation at Y1604 (Fig. 1b), an indicator of ALK activation.^32^ This is consistent to recent report indicating that ALK can be activated by RNase1.^20^ We then examined RNase1 expression and ALK activation in 13 lung cancer cell lines and an immortalized human bronchial cell line HBE4-E6E7. Among those, only four lung cancer cell lines exhibited RNase1 and ALK co-expression with ALK phosphorylation (Supplementary Fig. 1a), suggesting that RNase1 is required for ALK activation in lung cancer cells. RNase1 induced ALK phosphorylation at Y1604 and Y1282/1283 (Fig. 1c). The interaction between RNase1 and ALK was validated in HeLa cells co-expressing exogenous ALK and RNase1 (Fig. 1d). In addition, in vitro binding affinity assay revealed a dissociation constant (Kd) of 43 nM between RNase1 and ALK (Fig. 1e) comparable to ALKAL2/ALK (40 nM)^29^ and stronger than RNase1/EphA4 (92.4 nM)^26^, which is close to the actual concentration of RNase1 in the human serum^26^ (see later in Fig. 4a) and similar to that of other ligand-RTK binding.^33,34^ We then carried out an immunofluorescence microscopy showing ALK co-localized with RNase 1 after RNase1 treatment (Fig. 1f). The results from Duo link assay demonstrated the RNase1-ALK binding in vivo in cells co-expressing RNase1 and ALK (Fig. 1g). In a time-course experiment, RNase1 rapidly activated ALK (within 1–5 min; Fig. 1h) similar to those observed during classical ligand-receptor activation.^33,34^ ALK activation by RNase1 also occurred in a dose-dependent manner (Supplementary Fig. 1b). To determine whether the catalytic activity of RNase 1 is required for ALK activation, we evaluated the interaction between a catalytic-deficient (via mutation) RNase1^35^ and ALK. Both WT and mutant RNase1 bound to and activated ALK in H1299 cells (Fig. 1i). These findings suggested that RNase1-mediated ALK activation occurs independently of its ribonuclease activity, consistent to the previous report in liver cancer cells. Our previous study found that RNase1 is a ligand of EPHA4 in breast cancer cells. In both EPHA4 wild-type or EPHA4 knockout lung cancer cells, RNase1 could bind to ALK (Supplementary Fig. 1c) and ALK did not bind to EPHA4 (Supplementary Fig. 1d), which suggested that the RNase1-ALK interaction was independent of EPHA4 in lung cancer cells.

### RDAA is an oncogenic driver in vitro and in vivo

**Fig. 2:**
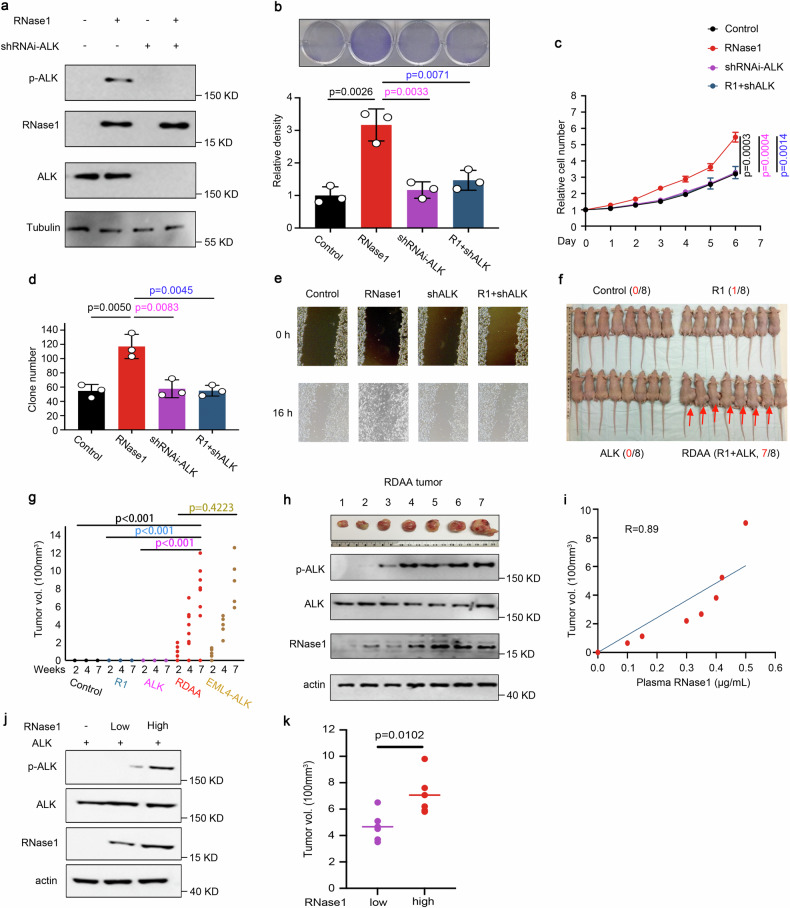
RDAA is an oncogenic driver in vitro and in vivo. **a** Western blot analysis of H1299 stable cells expressing wild-type RNase1 and/or short hairpin RNA (shRNA) to knockdown ALK with the indicated antibodies. **b** The indicated H1299 cells were subjected to MTT assay and cell viability was quantified. **p < 0.01, Student’s *t*-test. **c** Cell counting of the indicated cells was performed in triplicate and normalized to control. Error bars, mean ± SD. **p < 0.01. **d** Colony formation assay of the indicated cells. The relative number of colonies formed was measured in triplicate. **p < 0.01. **e** Wound healing assay of the indicated cells. Representative images shown. Scale bar, 500 mm. Quantification shown in Supplementary Fig. 2d. **f** Nude mice were injected with NIH3T3 cells or those expressing ALK and/or RNase 1. Tumor size was measured every 3 days. Red arrows pointing to tumors developed. **g** Quantification of tumors in (**f**). N = 8, control, R1, ALK and R1-ALK co-expression group. N = 5, ALK mutant group. **p < 0.01, Student’s *t*-test. NS, not significant. **h** Western blo*t* analysis of ALK-RNase1 tumors (N = 7). Actin was used as control. **i** Correlation between serum RNase1 concentration and tumor size. *R* = 0.89, Pearson’s Chi-Square test. **j** Western blot analysis of NIH3T3 cells with ALK and RNase1^high^ or RNase1^low^ expression. **k** Quantification of tumor size from mice injected with RNase1^high^ or RNase1^low^ NIH3T3 cells. N = 5. *p < 0.05, Student’s *t* test

The above findings prompted us to investigate the biological significance of the RNase1-ALK ligand-receptor pair in lung cancer cells. To this end, we first validated the expression of RNase1, ALK, and phospho-ALK in RNase1-expressing or ALK-knockdown H1299 cells by Western blotting (Fig. 2a). Ectopic expression of RNase1 in H1299 stable cells exhibited higher cell proliferation (Fig. 2b), cell growth (Fig. 2c), colony formation (Fig. 2d and Supplementary Fig. 2a), and cell mobility (Fig. 2e and Supplementary Fig. 2b) compared with control cells. In contrast, knocking down ALK attenuated those effects, suggesting the requirement of ALK for the RNase1-induced cell growth and mobility. Similar results were observed in H322 and H1355 lung cancer cell lines (Supplementary Fig. 2c, d). To further validate whether RNase1 is also required for the ALK activity, we knocked down RNase 1 in PC-9 lung cancer cells co-expressing endogenous ALK and RNase1 by two different small hairpin RNAs (shRNAs) targeting RNase1 (Supplementary Fig. 2e; lanes 2 and 3 vs. 1) and examined the effects on phospho-ALK, cell mobility and cell proliferation. Knocking down RNase1 attenuated the levels of phospho-ALK (lanes 4 and 5 vs. 1 and 2; Fig. S2e), and decreased cell proliferation (Supplementary Fig. 2f) and cell mobility (Supplementary Fig. 2g), all of which were restored by the addition of purified RNase1 proteins (described as in Fig. 1a). Together, these results suggested that the RNase1-driven-ALK-activation (RDAA) in lung cancer cells enhances cell proliferation, mobility and cellular transformation in vitro.

To examine the effects of the RDAA per se on tumorigenesis, we generated NIH-3T3 cells stably expressing ALK and/or RNase1. Compared with the ALK or RNase1 expression alone, cells co-expressing ALK and RNase1 (herein after referred to as RDAA cells) exhibited enhanced colony formation (Supplementary Fig. 2h). Interestingly, subcutaneous tumor generation was observed in nude mice (7/8) injected with RDAA NIH3T3 cells but not those expressing ALK (0/8) or RNase1 (0/8) alone and NIH3T3 control (0/8) (Fig. 2f). A comparison between mice injected with NIH3T3 cells which expressed EML4-ALK, (a constitutively activated ALK rearranged mutant, labeled as ALK mut; positive control^20^) and RDAA NIH3T3 cells revealed similar tumor growth (Fig. 2g). These results suggested that the RDAA, similar to the ALK mutant is an oncogenic driver. For tumors harvested from RDAA mice (N = 7) (Fig. 2f), increased expression of RNase1 was associated with increased p-ALK and a tendency toward larger tumors (Fig. 2h). Moreover, there was a strong positive correlation between mice serum RNase1 concentration and tumor size (*R* = 0.89; Fig. 2i), raising an interesting notion that RNase1 concentration and phospho-ALK may serve as biomarkers to identify the RDAA positive tumors. To further validate the relationship between RNase1 expression level and tumor growth, two different NIH3T3 cell lines with WT ALK and high or low level RNase1 (RNase1^High^ or RNase1^Low^) were subcutaneously injected into nude mice (Fig. 2j). Mice injected with RNase1^High^ NIH3T3 cells developed larger tumors than those with RNase1^Low^ (Fig. 2k). Together, these results indicated that the RDAA is an oncogenic driver.

### RDAA tumors are sensitive to ALK inhibitor in preclinical therapeutic models

**Fig. 3:**
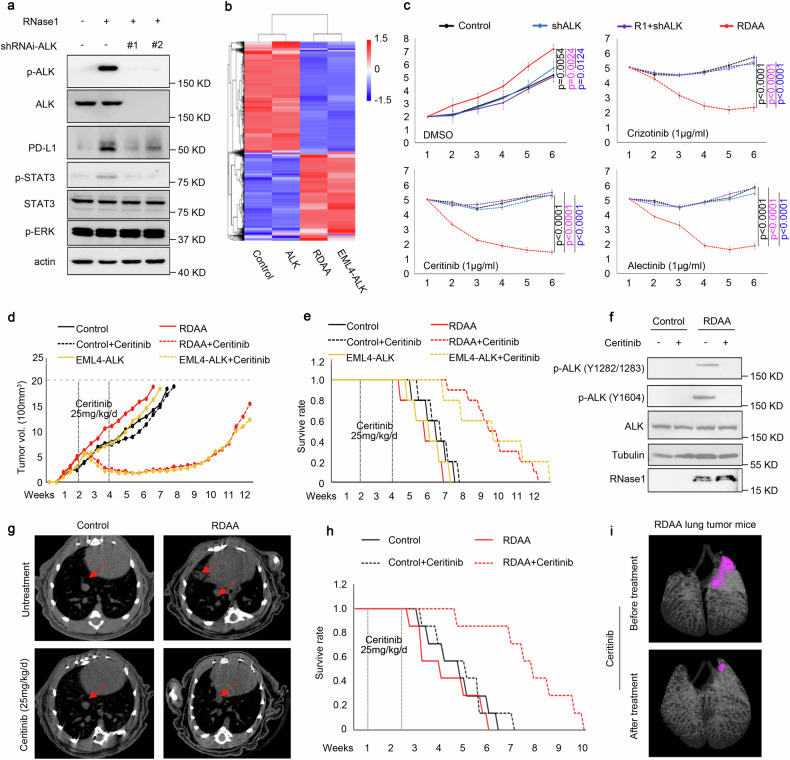
RDAA tumors are sensitive to ALK inhibitor in both subcutaneous and orthotopic mouse models. **a** Western blot analysis of ALK downstream signaling of RNase1-driven ALK-positive (RDAA) H1299 cells with the indicated antibody. Phospho-ERK1/2 T202/Y204 and phospho-STAT3 (Y705) specific antibodies were used to detect ERK1/2 and STAT3 phosphorylation, respectively. **b** RNA-seq analysis of control, RDAA and *ALK*-rearranged (EML4-ALK) H2228 lung cancer cells. **c** Cell counting of the indicated cells treated with or without (DMSO; control) the indicated ALK inhibitors. Experiments were performed in triplicate. **d** Mice received the indicate H1299 cells by subcutaneous injection, and when tumors reached 500 mm^3^, ALK inhibitor Ceritinib (25 mg/kg/d) was administered beginning day 14 for 2 weeks. Tumor size was measured every 3 days. N = 10. **e** Overall survival curve of mice in (**d**) starting the day of cell injection. **f** Western blot analysis of ALK phosphorylation with or without Ceritinib treatment. The tumor samples were collected from the mice in (**e**). **g** Representative Micro-CT scan images of mice who received an orthotopic injection of the indicated cells treated with or without Ceritinib (25 mg/kg/d). N = 7. Red arrows pointing to tumors. **h** Overall survival curve of mice in (**e**) starting the day of tumor transplantation. **p < 0.01, Log-rank (Mantel-Cox) test. **i** Representative 3D images of mice with orthotopic RDAA lung tumor before or after Ceritinib treatment. Area in pink indicate tumor size and location

*ALK* rearrangement in lung cancer has been reported to induce STAT3 signaling and upregulate immune checkpoint protein programmed cell death-ligand 1 (PD-L1).^36,37^ Because our results above indicated RDAA lung cancer cells harbor similar function and downstream signaling to those with ALK rearrangement, we asked whether RNase1-induced ALK activation also enhances STAT3 signaling and PD-L1 expression. As shown in Fig. 3a, RNase1 indeed upregulated p-STAT-3 and PD-L1, and knocking down ALK attenuated phospho-ALK, phospho-STAT3, and PD-L1 expression levels in the presence of RNase1 treatment. Simultaneously, flow cytometry results also demonstrated that activation of RDAA in the H1299 cell line significantly upregulated PD-L1 expression which were consistent with the high PD-L1 expression in the ALK rearrangement H3122cell line (Supplementary Fig. 3a). We also carried out phospho-kinase antibody array and showed that the AMPK, EGFR, S6K, c-Jun, and STAT3 signaling pathways were upregulated in both RDAA and ALK rearranged (ALK mut) H2228 cells (Supplementary Fig. 3b). RNA-seq analysis also indicated similar transcriptome changes between RDAA and ALK rearranged cells (Fig. 3b). ALK inhibitors are highly effective in patients with *ALK*-rearranged NSCLC.^17,18,21,22^ Thus, we also examined the effects of ALK inhibitors on RDAA lung cancer cells in vitro and in vivo. First, we treated H1299 cells that expressed RDAA or expressed either RNase1 or ALK alone with three U.S. Food and Drug Administration-approved ALK inhibitors, crizotinib, ceritinib, and alectinib, and showed that RDAA cells were more sensitive to all three ALK inhibitors compared with those expressing RNase1 or ALK alone (Fig. 3c). Similar results were obtained in H1355 and H322 lung cancer cells (Supplementary Fig. 3c, d). Next, mice subcutaneously injected with H1299 control (without RNase1 expression), RDAA (ALK and RNase1 co-expression), or ALK mut lung cancer cells (EML4-ALK expression) were treated with ceritinib for two weeks (25 mg/kg/day). Ceritinib treatment slowed tumor growth (Fig. 3d) and improved survival (Fig. 3e) in mice injected with RDAA and *ALK*-rearranged (EML4-ALK) H1299 cells compared with control mice. Similar results were observed in RDAA xenograft subcutaneous tumor models treated with crizotinib (Supplementary Fig. 3e, f). To mimic organ-specific microenvironment of tumor growth and drug treatment, we established orthotopic lung cancer mouse models^38^ and evaluated the efficacy of ceritinib. Compared with the control group, mice bearing RDAA orthotopic tumors were more sensitive to ceritinib treatment (Fig. 3f–i). ALK phosphorylation at Y1604 and Y1282/1283 were inhibited under ceritinib treatment (Fig. 3f). RDAA mice with ceritinib treatment had smaller tumors (Fig. 3g) and better overall survival (Fig. 3h). Three-dimensional images from micro-CT scan revealed a substantial reduction in RDAA lung tumor after ceritinib treatment (Fig. 3i). The results from in vitro and in vivo studies demonstrated that ALK inhibitors are also effective in RDAA lung cancers, suggesting that the RDAA is not only an oncogenic driver but also an effective therapeutic target. This was further supported by the evidence that RNase1/ALK co-expression is significantly correlated with worse survival in patients with lung cancer from the TCGA database (Supplementary Fig. 4a, b).

### NSCLC patients with RDAA positive demonstrate response to ALK inhibitor

**Fig. 4:**
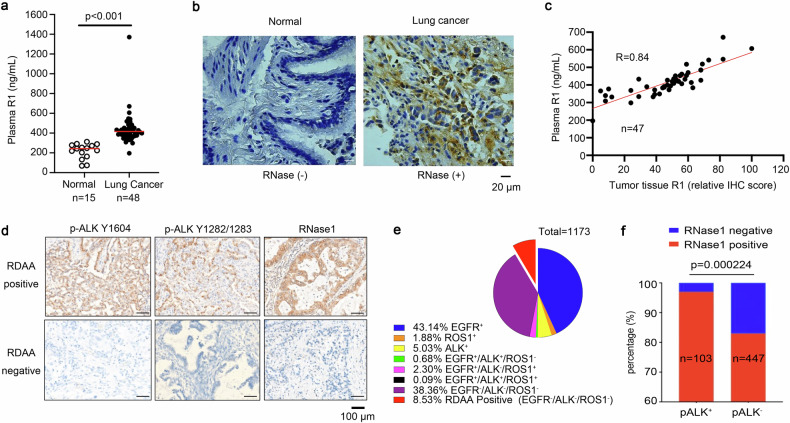
Identification of patients with RDAA NSCLC. **a** Quantification of plasma RNase1 in NSCLC patients (N = 48) and normal individuals (N = 15). RNase1 concentration was measured by ELISA as described in Methods. *p* < 0.01, Student’s *t*-test. **b** IHC staining of RNase1 in human lung tumors (N = 48) and normal lung (N = 10) tissues. RNase1 expression level was calculated based on the intensity and percentage of stained cells as described in Methods. Representative images shown. **c** Correlation analysis between plasma RNase1 concentration and RNase1 expression level in paired tumor tissues (N = 47). *R* = 0.84, Pearson’s Chi-Square test. **d** IHC staining of RNase1 expression and ALK phosphorylation levels in human NSCLC tissues. ALK p-Y1604, ALK p-Y1282/1283 and RNase1 specific antibodies were used for IHC staining. Representative images shown. **e** Diagnosis of RDAA NSCLC patients in 1173 NSCLC tissues. ALK p-Y1604, ALK p-Y1282/1283 and RNase1 were used as biomarkers to identify RDAA positive samples. **f** Correlation analysis between RNase1 expression and ALK phosphorylation in NSCLC tissues which used from (**e**). ALK p-Y1604 and 1282/1283 double positive means ALK phosphorylation positive (pALK + ), otherwise means ALK phosphorylation negative (pALK-)

The above in vitro and in vivo results prompted us to investigate the clinical potential of the RDAA status as a therapeutic target in patients. We obtained tumor tissues and plasma samples from 48 patients with NSCLC without ALK rearrangement and 15 normal individuals. The average plasma RNase1 concentrations in NSCLC patients were significantly higher relative to the normal individuals (436.74 ± 159.2 vs. 218.15 ± 76.63 ng/ml; Fig. 4a). Immunohistochemistry (IHC) staining also indicated higher levels of RNase1 level in the tumor tissues than normal lung tissues (Fig. 4b). There was a strong positive correlation between tumor RNase1 level and paired plasma RNase1 concentration (*R* = 0.84; Fig. 4c), suggesting that plasma RNase1 have mainly originated from the patient’s tumor tissue. Subsequently, we used three monoclonal antibodies of RNase1, p-ALK Y1604 and p-ALK Y1282/1283 to identify RDAA positive tumors by tissue IHC. These antibodies were generated from mice and identified through western blotting, both p-ALK Y1604 and p-ALK Y1282/1283 antibodies could detect RNase1 induced phosphorylation of wild type ALK but not for mutant ALK in which the tyrosine (Y) sites were mutated into non-phosphorylatable phenylalanine (F) sites (Supplementary Fig. 4c). Based on previous experimental results, we defined the tumor as RDAA positive only when the IHC staining results of the above three antibodies were triple-positive (Fig. 4d, the antibodies of RDAA, the protocol for immunohistochemical staining and positive determination of RDAA were supported by Betta Pharmaceuticals Co., Ltd, more details see Methods).

We analyzed the RDAA positive rate from two independent cohorts of NSCLC samples. In a small cohort, five patients (10.4%, 5/48) who had positive p-ALK Y1604, p-ALK Y1282/1283 and RNase1 staining were categorized as the RDAA positive group. Interestingly, the average plasma RNase1 level was significantly higher in patients with RDAA than non-RDAA NSCLC group (426.654 ± 164.52 vs. 523.53 ± 56.57 ng/ml; Supplementary Fig. 4d). In another large cohort, 100 of 1173 (8.53%) NSCLC patients’ tumor were identified as RDAA positive that does not have known and targetable EGFR/ALK/ROS1 mutation or rearrangement (Fig. 4e, Supplementary Tables 3–6). This means about 8–10% of NSCLC patients were RDAA positive and could benefit from ALK inhibitor treatment. Also, there was a good correlation between RNase1 and ALK phosphorylation through the tumor tissue IHC (Fig. 4f). Patient-derived xenograft mouse model showed, RDAA positive tumor were sensitive to ALK inhibitor crizotinib treatment (Supplementary Fig. 4e).

**Fig. 5:**
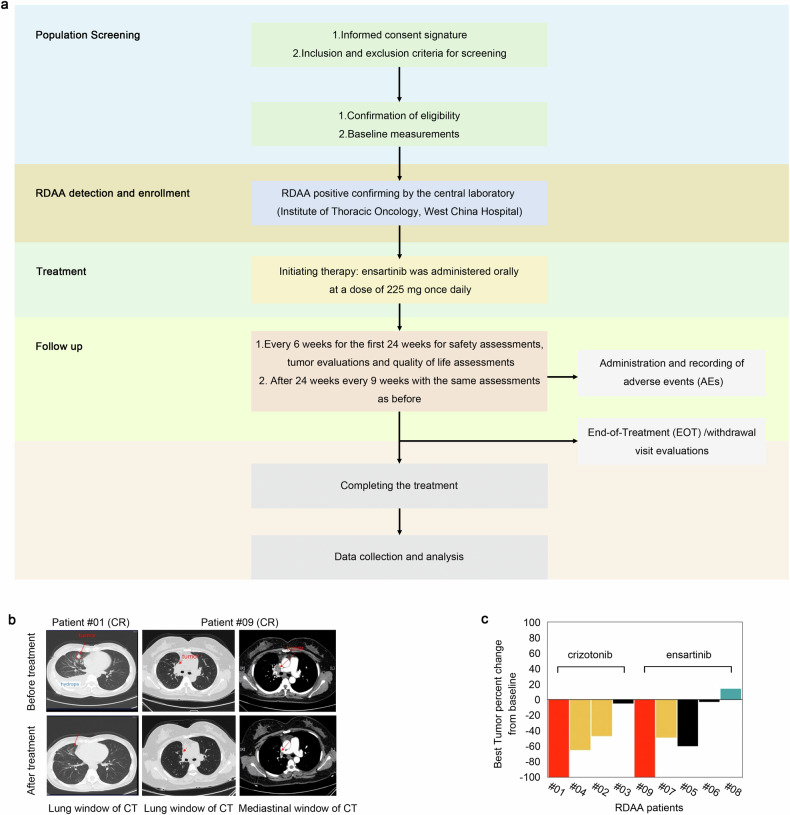
Patients with RDAA NSCLC benefit from ALK inhibitor in clinical study. **a** The flowchart of participant selecting process. **b** Clinical CT scan images of RDAA NSCLC patients before and after ALK inhibition treatment. Red arrows pointing to tumors. Patient #01 and Patient #09 demonstrated complete response (CR) based on Response Evaluation Criteria in Solid Tumors (RECIST). Representative images shown. **c** Best tumor percent change from baseline after ALK inhibition treatment of 9 RDAA NSCLC patients. SD, stable disease, black column represented; PR, partial response, yellow column represented; CR complete response, red column represented; PD progression disease, bottle green column represented. Total response rate is 88.9% (RECIST)

Subsequently, we registered and conducted a clinical therapy study targeting RDAA positive tumor (the study had been approved by the Biomedical Ethics Review Committee of West China Hospital of Sichuan University Ethics (Approval No.2021-1669) and registered in the Chinese Clinical Trial Registry (Registration number: ChiCTR2100054794). After eliminating known and targetable oncogenic genetic alterations (e.g., EGFR mutation(s), ALK rearrangement, ROS1 rearrangement, c-Met mutation(s), and c-Met amplification, Table S1) by tumors genetic testing and confirming no ALK rearrangement/fusion by ALK (D5F3) VENTANA immunohistochemical test (Supplementary Fig. 5b and Supplementary Table 1), lung adenocarcinoma patients having failed first-line therapy with RDAA-positive and PD-L1 < 20% were enrolled and started the ALK inhibition treatment. Details regarding the screening, recruitment, and clinical treatment procedures for RDAA-positive patients were described in Fig. 5a. Details of inclusion and exclusion criteria were described in Materials and Methods part and study protocol sections of the supplementary materials. At present, four eligible lung adenocarcinoma patients with RDAA-positive volunteered to received ensartinib. (ALK-TKI, 225 mg /day for 4 weeks, Fig. 5b and Supplementary Fig. 5a). One patients achieved partial response (PR, patient#07,according to Response Evaluation Criteria in Solid Tumors [RECIST]^39^) and two of them had tumor control with a response of stable disease(SD, patient #05 and patient #06). One patient did not respond to therapy with tumor progression after treatment (PD, patient #08). Particularly, another eligible RDAA^+^ patient who also received ensartinib treatment but was not enrolled in clinical trial for travel reason and achieved complete response (CR, #09, Fig. 5b). In addition, four lung adenocarcinoma RDAA^+^ patients received crizotinib therapy (another ALK inhibitor, 250–500 mg /day for 4 weeks, Fig. 5b and Supplementary Fig. 5a), three out of four patients achieved objective response: one complete response (CR, patient #01, Fig. 5b) and two partial response (PR, patients #02 and #04). One patient had stable disease (SD, patient #03). Totally, the objective response rate (ORR) and disease control rate (DCR) of our clinical study were 55.6% and 88.9% (Fig. 5c), respectively, suggesting that RDAA was a promising therapeutic target for anti-tumor therapy.

It was worth mentioning that 4 patients who received crizotinib treatment had currently survived till the last follow-up visit data, and their overall survival after receiving ALK inhibitors has exceeded 35 months (Supplementary Table 2). One of the patients (#02) had developed brain metastases at 10 months after receiving crizotinib, and then this patient accepted the third generation ALK inhibitor lorlatinib treatment, partially responded, and continued survival more than 2 years (Supplementary Table 2). These data highlight the significance of the effective targeted therapy for patients with RDAA^+^ NSCLC.

## Discussion

Approximately 35–50% of NSCLC patients without tyrosine kinase mutations or rearrangements (non-mutated) fail to benefit from current targeted therapies. In current study, we focus on the oncogenic mechanism in non-small cell lung cancer patients without typical gene mutations or rearrangements/fusion and report a non-canonical role of RNase1, a human ribonuclease superfamily protein, as a ligand of wild-type ALK. RNase1 binds to and activates ALK and its downstream signaling pathway. To distinguish ALK rearrangement and other known oncogenic drivers from RNase1-mediated ALK activation, we named this phenomenon RNase1-driven ALK-activation (RDAA). NIH 3T3 cells with RDAA expression generates subcutaneous tumors in nude mice, indicating that the RDAA is an oncogenic driver. RDAA-positive NSCLC cells exhibited higher sensitivity to U.S. Food and Drug Administration (FDA)-approved ALK inhibitors compared to cells expressing RNase1 or ALK alone. In addition, tumor growth suppression and survival rates were improved substantially in both orthotopic RDAA NSCLC cell line-derived and patient-derived xenograft tumor models following ALK inhibitor (crizotinib or ceritinib) treatment. Clinical data analysis further revealed that co-expression of RNase1 and ALK strongly correlated with poorer survival outcomes in lung cancer patients.

Moreover, we developed a method to identify RDAA in human NSCLC tissue specimens using phospho-ALK and RNase1 immunohistochemical staining, identifying RDAA in 8.5% (100/1173) of patients. A clinical therapy study targeting RDAA positive tumor was then conducted and 9 patients received crizotinib or ensartinib treatment, achieving an objective response rate of 56%, including two complete responses. Notably, all patients treated with crizotinib showed prolonged survival, with some exceeding 35 months. Based on these findings, we postulated that a significant portion of patients with NSCLC (~8.5%) whose tumors are associated with novel oncogenic driver RDAA, should respond to the FDA-approved ALK inhibitors. Our findings provide not only the scientific basis but also the clinical response to encourage larger clinical studies to promote targeting RDAA as a first-line therapy option in NSCLC.

Recent clinical studies have demonstrated that NSCLC patients harboring tumor driver genes such as EGFR mutations^40^ or ALK rearrangement/fusion^41^ are more prone to developing metastases beyond the primary tumor site. Given the similarities in tumor-driving mechanisms between RDAA and EML4-ALK rearrangement/fusion reveal by this study, we hypothesize that RDAA-positive tumors may also exhibit a strong metastatic potential. However, due to the ongoing of our clinical studies, we currently lack sufficient clinical trial data to confirm the influence of RDAA on tumor metastasis. We aim to collect comprehensive clinical data from a larger patient cohort and perform mechanistic studies to further validate the role of RDAA in promoting tumor metastasis.

Previous studies have shown that WT ALK was predominantly expressed in most neuroblastomas and rare in normal cells or non-ALK rearrangement/fusion tumors.^42^ Consistent with these findings, our research revealed that both mRNA transcription and protein expression level of ALK in non-ALK-rearranged tumors is significantly rare, detectable only in a few tumor cell lines (e.g., H1299, H322, and H1355), and markedly lower when compared to ALK rearrangement/fusion cell lines(see Supplementary Figs. 1a, 6a, b). Despite this low expression, our results establish that even minimal ALK levels are sufficient to trigger RDAA and drive tumor progression. Notably, ALK rearrangement/fusion-driven tumors produce abundant ALK fusion protein, enabling detection by ALK VENTANA IHC tests. In contrast, RDAA-driven tumors cannot be identified by IHC due to their low ALK expression (see Supplementary Fig. 6c), which highlights a critical limitation in the current diagnostic approach for non-ALK-rearranged tumors: low ALK expression is often overlooked by standard clinical tests, such as IHC and FISH, as recommended by NCCN guidelines. Consequently, patients with RDAA-positive tumors may not receive appropriate targeted therapy. To overcome this challenge, we propose a novel RDAA-positive diagnostic standard, employing three specific antibodies (RNase1, p-ALK Y1604, and p-ALK Y1282/1283) to screen for lung cancer patients with low ALK expression but functional activation by RNase1.Our rigorous protocol ensures a high level of diagnostic accuracy and reliability, which could partially eliminate similar family proteins’ phosphorylation. Furthermore, through subsequent analysis of data from the ongoing clinical trials, we aim to further refine and simplify the criteria and standards for identification of RDAA positivity, which would facilitate the broader application of RDAA-targeted therapies and offering new treatment options to non-mutated NSCLC patients.

It had been reported that ALKAL1/2 were the ligands of ALK and LTK.^43–45^ We found that RNase1 was the ligand of ALK but not LTK (data not shown). ALKAL1/2 and other ligands of ALK may also contribute to induce ALK activation depending on cell types. However, we have not yet obtained direct evidence supporting ALKAL as a ligand capable of activating ALK. Moreover, our preliminary data revealed no significant difference in plasma ALKAL1 levels between lung cancer patients and healthy individuals. Therefore, whether ALK activation mediated by ALKAL1/2 contributes to tumor progression still needed to be confirmed by further studies.

In addition, RDAA positive was identification in 19 of 100 tumor tissues of lung squamous cell carcinoma (19%, Supplementary Fig. 7) which was consistent with the RDAA positive proportion in lung adenocarcinoma. Given that squamous cell lung cancer was not an indication for targeted therapy because of its low rate of known oncogenetic driver mutation and unsatisfactory response to targeted therapy,^46,47^ we supposed that RDAA positive could be a potential target for targeted therapy in lung squamous cell carcinoma after verifying by further clinical treatment studies.

Furthermore, we also found that a few pancreatic ductal carcinoma (PDAC) samples were RDAA positive (Supplementary Fig. 8, down row) which suggested that RDAA not only played as an oncogenic driver in lung cancer but may also have a promoting effect on other tumors.

It was known that the coexistence of oncogenic mutation driven by genomic RTKs alterations was mutually exclusive in NSCLC,^48,49^ but the coexist of RDAA with other RTKs alterations was unclear. Remarkably, we found a certain proportion of RDAA-positive cases from NSCLC patients with EGFR mutation (Supplementary Fig. 8, up row) suggesting that RDAA could coexist with other mutation-driven tumorigenic factors. The coexistence of RDAA may potentially alter the outcomes of TKI treatments, suggesting that screening for RDAA in patients with mutated NSCLC could be warranted.

In summary, our results revealed a non-canonical role of RNase1 as a ligand for ALK activation. We established a clinically applicable protocol for identification of RDAA-positive patients: RNase1 and phospho-ALK staining were used as the molecular diagnostic markers. To date, there are no suitable targeted therapies for non-mutated NSCLC patients. Our findings suggested that ALK inhibitors could be repurposed to treat RDAA-positive NSCLC patients, providing a new therapeutic avenue for a subset of patients who previously had limited treatment options.

## Materials and methods

### Cell culture

NSCLC cell lines, HBE4-E6E7 cells, HEK293T cells and NIH3T3 cells were purchased from the ATCC. HBE4-E6E7 cells were maintained in keratinocyte serum free medium (K-SFM; Cat.17005-042, GIBCO) with 10% fetal bovine serum, 0.05 mg/ml bovine pituitary extract (BPE), 5 ng/ml epidermal growth factor (EGF) and 10 ng/ml cholera toxin. Other cells were maintained in Dulbecco’s modified Eagle’s medium/F-12 medium with 10% fetal bovine serum and antibiotics. All cell lines were validated by short tandem repeat (STR) DNA fingerprinting at MD Anderson Cancer Center (Houston, TX) and negative for mycoplasma infection.

### RNases purification and activity assay

Each RNase cDNA was cloned into a prokaryotic expression vector PSJ3 with N-terminal 6His tag. Plasmids expressing His-tagged RNase were used to transform E. coli BL21 cells. A single transformant was selected and cultured in 5-ml LB medium at 37 °C overnight. When OD reached >1.2, cells were transferred to a 250-ml LB medium and incubated at 37 °C with shaking for about 2 h. The cells were induced with 0.5 mM IPTG for 20–24 h at 16 °C with shaking to express RNase when OD increased to between 0.5 and 0.6. After IPTG induction, cells were collected, lysed in lysis buffer (50 mM Tris-HCl, pH 7.5, 150 mM NaCl), sonicated, supernatant loaded onto the HisTrap column (GE) slowly. The column was washed with wash buffer containing 50 mM Tris-HCl, pH 7.5, 150 mM NaCl, 10 mM imidazole, and RNases eluted by elution buffer containing 50 mM Tris-HCl, pH 7.5, 150 mM NaCl, 300 mM imidazole. The RNase activity was detected by an Ambion RNase Alert Lab Test Kit (Life Technologies AM1964) following the manufacturer’s protocol.

#### Stable cell lines

For ALK, RNase1, and EML4-ALK stable expression cell lines, the cDNA of each of the above genes was cloned into a modified pCDH-CMV-MCS-EF1-Puro vector (CD510B-1; System Biosciences) or pCDH-CMV-MCS-EF1-Neo (CD514B-1; System Biosciences) vector. Site-specific mutant of RNase1 was generated by a Site-Directed Mutagenesis Kit (NEB E0554S). The lentiviral shRNAs were used to knock down ALK and RNase1 (Sigma). HEK293T cells were transfected with the indicated plasmids and packaging plasmids (VSV-G and dvpr) to generate viruses. H1299, H322, H1355 and NIH3T3 cells were infected with above viruses for 6–8 h with polybrene (10 g/ml) and then subjected to puromycin (2 g/ml) or G418 (2000 g/ml) selection to generate stable transfectants. Lipofectamine 2000 (Life Technologies) was used for HEK293T transfection.

### Antibodies and reagents

Antibodies against RNase1 (Sigma HPA001140), ALK (Cell Signaling 3633), phosphorylated ALK (Tyr1604; Cell Signaling 3341S), phosphorylated ERK1/2 (Thr202/Tyr204; Cell Signaling 9101), ERK1/2 (Cell Signaling 9102), STAT3 (Cell Signaling 9132), phosphorylated STAT 3 (Cell Signaling 9145S), PD-L1 (Cell Signaling 13684 s), and tubulin (Sigma B-5-1-2) were used for immunoblotting, immunohistochemistry, immunofluorescence, and Duolink assays. PE anti-mouse CD274 (B7-H1, PD-L1) Antibody (Biolegend 124307) was used for flow cytometry. For ALK inhibitor crizotinib (PF-02341066), ceritinib (S7083) and alectinib (CH5424802) were purchased from Selleckchem. The ELISA Kit for human RNase1 (SEA297Hu) and FAM150 (MBS9328956) were used to measure their plasma concentration. Plasma (5 µl) from patients or mice were added to 195 µl phosphate-buffered saline (PBS; 1:40 dilution) and then subjected to ELISA assay based on the manufacturer’s protocol.

### Animal experiments

All animal experiments were performed under the guidelines and protocols approved by the MD Anderson Institutional Animal Care and Use Committee (IACUC). The nude male mice were purchased from Jackson Laboratory. In in vivo tumorigenesis assay, 1 × 10^6^ indicated stable NIH-3T3 cells were subcutaneously injected into 6-week-old male nude mice (for NIH3T3 control, ALK, RNase1 and RDAA group, n = 8/group; for EML4-ALK group, n = 5/group). Tumor volumes were monitored using external calipers and calculated by (length × width^2^)/2. In the subcutaneous model, 6 groups of 48 nude mice were subcutaneously injected with 2 × 10^5^ stable H1299-control, H1299-RDAA or H1299-EML4-ALK cells. Tumor-bearing mice in both groups were further placed in two groups (n = 8/group) for treatment with ceritinib (25 mg/kg/day), crizotinib (50 mg/kg/day), or vehicle control. Drugs were administrated via oral every day for 2 weeks. Tumor volume was measured every 3 days. In the orthotopic model, subcutaneous H1299-Control and H1299-RDAA tumors were cut into cubes (about 1 mm^3^) and then were transplanted into the lung tissue of nude mice under isoflurane anesthetized. Mice were given ceritinib (25 mg/kg/day) or matched vehicle control at 7 days after inoculation, and continuous for two weeks. The orthotopic tumors were evaluated by micro-CT scanner (Fig. 3f). At the endpoint of experiments, mice were killed by CO2 exposure and cervical dislocation according to the guidelines.

### Clinical samples study

Paired plasma samples from the 48 patients with NSCLC were analyzed by ELISA to measure RNase1 plasma concentration (SEA297Hu). For immunohistochemistry, paraffin-embedded archived lung cancer tissue sections were submerged in xylene to remove the paraffin, and rehydrated with an ethanol gradient. H2O2 (3%) was used to block endogenous peroxidase activity, and antigen retrieval was carried out by incubating sections in sodium citrate with microwave heating. Slides were cooled to room temperature for 1 h. The sections were blocked with 5% goat serum (Beyotime Biotechnology) and then incubated overnight with ALK (1:50, CST) or p-ALK (1:100, CST) antibody at 4 °C. After washing with PBS, sections were incubated with HRP-conjugated secondary antibody (Abcam) at room temperature for 1 h. Sections were developed for visualization using diaminobenzene. Study of tissue staining and plasma samples from the patients was approved by the Ethics Committee of Cancer Hospital Affiliated to Harbin Medical University (IRB number: KY2019-18).

### Clinical study for RDAA+ patients

The clinical therapy study for RDAA+ patients received approval from the Biomedical Ethics Review Committee of West China Hospital of Sichuan University (Ethics Approval No. 2021-1669) and was duly registered with the Chinese Clinical Trial Registry (Registration No: ChiCTR2100054794, accessible at https://www.chictr.org.cn/showproj.html?proj=144966, Statistical Analysis Plan (SAP) and Study Protocol of the clinical study was shown in Supplementary Materials). Prior to undergoing treatment, patients were provided comprehensive information and subsequently signed an informed consent form, adhering strictly to the principles outlined in the Declaration of Helsinki.

Flowchart of the participant selecting process is shown in Fig. 5, and the eligibility criteria for patient inclusion were meticulously defined as follows:

a) Age requirement of ≥18 years, encompassing both genders. (b) Confirmed histological or cytological diagnosis of locally advanced or metastatic non-small cell lung cancer (NSCLC). (c) Molecular testing (Next-Generation Sequencing, NGS) ensuring the absence of known EGFR, KRAS, BRAF sensitive mutations, NTRK, ALK, ROS1, RET rearrangements, or MET gene alterations (amplification and exon 14 jump mutations) in tumor samples. (d) Expression of PD-L1 below 20%, validated through the VENTANA PD-L1(SP263) assay. (e) RDAA-positive confirmation of biopsy tissue specimens through laboratory testing conducted at West China Hospital. (f) Documented disease progression post-standard treatment (according to RECIST 1.1 criteria) or demonstrated reluctance or intolerance to first-line therapy. (g) Eastern Cooperative Oncology Group (ECOG) performance status score of 0−2. (h) Anticipated survival of ≥3 months. (i) Satisfactory physiological parameters and organ function. (J) Asymptomatic central nervous system (CNS) involvement not necessitating steroid or anticonvulsant therapy for metastasis. (k) Presence of a measurable lesion as per RECIST 1.1 criteria. (l) Resolution of drug-related toxicity reactions, excluding alopecia, to grade 2 or lower (according to CTCAE 4.03 criteria). (m) Demonstrated willingness and capacity to adhere to trial protocols and follow-up procedures, coupled with a comprehensive understanding of the trial’s nature, as evidenced by voluntary signature on the written informed consent form.

The patient exclusion criteria were rigorously outlined as follows:

(a) Confirmation of known oncogenic driver mutations such as EGFR, KRAS, BRAF, NTRK, ALK, ROS1, RET rearrangement, or MET gene alterations (amplification and exon 14 jump mutations), or PD-L1 expression ≥20%. (b) Pathological findings suggestive of mixed tumors. (c) Concurrent administration of other systemic anti-tumor therapies. (d) History of malignancy other than lung cancer within the past 3 years (excluding cured cutaneous basal cell tumors, endoscopically resected early gastrointestinal tumors, and cervical carcinoma in situ). (e) Participation in another investigational drug clinical trial within 4 weeks or undergoing major surgery (including stem cell transplantation or organ transplantation, immunotherapy, or radiation therapy) prior to the initial drug dose. (f) Presence of severe cardiovascular disease or cardiovascular abnormalities within 6 months preceding the first dose. (g) Significant medical conditions affecting drug absorption, distribution, metabolism, and excretion (e.g., swallowing dysfunction, active gastrointestinal disorders, etc.). (h) Positive serology for active hepatitis B, hepatitis C viruses, HIV, and syphilis. (i) History of prior interstitial lung disease, drug-induced interstitial lung disease, radiation pneumonitis requiring steroid therapy, or evidence of clinically active interstitial lung disease. (j) Women of childbearing potential or breastfeeding females with a positive serum pregnancy test within 7 days before treatment initiation, or both males and females not using effective contraception or planning to conceive during treatment and up to 3 months post-treatment completion. (k) Known history of anaphylactic reaction to ensartinib or any of its excipients. (l) Use of medications within 14 days prior to the first dose or requiring a combination of medications associated with a risk of QTc prolongation and/or torsades de pointes, or a strong CYP3A inhibitor or inducer during treatment. (m) Anticoagulant therapy with warfarin or any other coumarin derivative. (n) Presence of severe acute or chronic medical conditions, deemed by the investigator to increase the risk associated with study participation or interfere with the interpretation of study results.

### Clinical trial procedure

Before undergoing screening assessment, patients must receive full information and sign an informed consent form. The screening phase must be completed within 28 days prior to dosing. Enrollment in the study was contingent upon the investigator confirming adherence to inclusion criteria and absence of exclusion criteria. Subsequently, patients were provided with the study drug and a dosing diary card. RDAA-positive screening was uniformly conducted by the central laboratory (Institute of Thoracic Oncology, West China Hospital).

Upon initiating therapy, ensartinib was administered orally at a dose of 225 mg once daily until disease progression, development of intolerable toxicity, decision by the investigator or patient to withdraw, loss to follow-up, initiation of other antineoplastic therapy, or death. Patients were assessed every 6 weeks for the first 24 weeks of the trial for safety assessments (including physical examination, vital signs, ECOG performance status score, routine blood tests, blood biochemistry, coagulation studies, routine urine analysis, routine stool examination with occult blood test, 12-lead electrocardiogram, and hormone panel), tumor evaluations (thoracic/abdominal/pelvic contrast-enhanced CT scans, cranial MRI), and quality of life assessments. After 24 weeks, visits occured every 9 weeks with the same assessments as before. Patients experiencing their first remission or disease control (complete response, partial response, or stable disease according to RECIST 1.1 criteria) at a visit will undergo a review 4 weeks later to confirm the evaluation results.

For patients completing the trial or withdrawing informed consent, all adverse events (AEs) and drug combinations must be documented up to 30 days after the last trial dose, and any new AEs occurring within 30 days of the last trial dose must be reported. All AEs must be monitored until resolved or stabilized unless deemed unrelated to the trial medication and attributable to the primary disease. Non-severe AEs, determined by the investigator to be unrelated to the trial medication, will not be recorded for patients who initiate other antineoplastic therapy.

End-of-Treatment (EOT) visit evaluations were conducted promptly after the patient discontinues the trial drug. Any patient who terminated treatment or withdrew from treatment for reasons other than disease progression should undergo a safety assessment promptly, while continuing to undergo tumor assessments at the same frequency as during the treatment period until disease progression occurs or other antineoplastic therapy was initiated. However, patients whose treatment termination was due to disease progression should only undergo safety assessment, and no further tumor assessments should be conducted. If a patient discontinues treatment at the final visit due to toxicity or other reasons and did not continue to take the test drug thereafter, the visit was considered an end-of-treatment/withdrawal visit.

Survival Follow-up and Efficacy Assessment:

Patients who experienced disease progression or initiated other anti-tumor therapies were no longer subject to safety and tumor assessments but continued to be followed up via telephone every 12 weeks. During these follow-up calls, information regarding overall survival and subsequent treatment was collected until death or loss to follow-up. The outcome of this study was the reduction of tumor size after combination therapy, and the efficacy of the treatment regimen was determined according to the RECIST 1.1 criteria, which were as follows: complete remission (CR): disappearance of all target foci, and the short diameter of all pathological lymph nodes (both target and non-target) must be reduced to <10 mm; partial remission (PR): reduction of at least 30% in the sum diameter of the target foci compared to baseline; progression of disease (PD): relative increase in the sum of diameters of target lesions of at least 20%, referenced to the smallest of the sums of the diameters of all target lesions measured throughout the experimental study (or to baseline if baseline measurements are smallest), and in addition, an increase in the sum of the diameters of the diameters in absolute terms of at least 5 mm must be met (the presence of one or more new lesions is considered to be disease progression); stable disease (SD): a decrease in target lesion size that did not reach PR and an increase in target lesion size that did not reach PD, and in between, with reference to the smallest of the sums of the diameters of the target lesions studied.

Patients #05-#08 in this study received ensartinib treatment(225 mg /day). Simultaneously, patient #09 also met the inclusion criteria and voluntarily signed the consent form to receive ensartinib treatment. However, patient #09 resided in Zhejiang Province, which was 1853.3 km away from our study center located in Chengdu. Due to the unwillingness to travel such a long distance for treatment and follow-up, the patient was not enrolled in the clinical study. Instead, the patient was treated as an observational case and received the same ensartinib treatment and follow-up regimen at the local hospital and voluntarily signed informed consent to share the clinical data collected during the treatment period.

Additionally, during the period shortly before the start of this clinical study, we collaborated with the Department of Thoracic Surgery at Harbin Medical University Cancer Hospital to screen and identify 5 cases of RDAA-positive NSCLC patients. Four of them (Patient #01-#04) voluntarily signed the consent forms and began receiving crizotinib treatment (250–500 mg/day). The follow-up procedures for these 4 patients after treatment were consistent with those of patients #05-#08 and maintained for 3 years.

### RDAA detection assay

The antibodies of RDAA, the protocol for immunohistochemical staining and positive determination of RDAA were supported by Betta Pharmaceuticals Co., Ltd and Hangzhou Repugene Technology Co., Ltd.

Immunohistochemical staining procedures by using RDAA antibodies:

The primary antibodies used in this study during the immunohistochemical staining were all murine monoclonal antibodies. After deparaffinizing and rehydrating, the paraffin sections were repaired with high-temperature microwave (EDTA antigen repair solution: pH 8.0 (Beijing Zhong Shan -Golden Bridge Biological Technology Co., Ltd., cat: ZLI-9067, China), heated for 8 min each, two times), blocked with 3%H2O2 (20–30 min) and 10% goat serum (30 min), respectively. Then 100ul diluted p-ALK Y1604 (dilution ratio 1:500, antibody concentration 0.5 mg/ml), p-ALK Y1282/1283 (1:250, Antibody stock concentration 0.5 mg/ml),RNase1 (dilution ratio 1:500, antibody stock concentration 0.5 mg/ml) were incubated to cover the surface of the tissue sections overnight at 4 °C(The production steps of RDAA-related antibodies have been described in the previous article and will not be repeated in this article). Subsequent overnight sections were incubated with mouse secondary antibody diluted in PBS (dilution ratio: 1:250, cat: 115-035-003, Jacson, United States) at room temperature for 40 min and then stained with SignalStain® DAB Substrate Kit (Cat. 8059S, CST, USA) (color reaction time: p-ALK Y1604: 40 s, p-ALK Y1282/1283:20 s, RNase1:40 s). After DAB color development, the slices were nucleated with hematoxylin, separated with hydrochloric acid alcohol, returned to blue with ammonia water, dehydrated with absolute ethanol, and then sealed.

Immunohistochemical staining scoring rules of RDAA antibodies:

The positive expression intensity of RDAA was evaluated in tissue sections with tumor area greater than 70% in the field of high-power microscope (10x eyepiece + 40x objective). Whether a lung cancer tumor section was judged to be RDAA positive or not was comprehensively determined according to IHC intensity scores of the three antibodies: p-ALK Y1604, p-ALK Y1282/1283 and RNase1.The score formula was as follows: score of the proportion of positive staining cells of tumor cells * Staining intensity score.

- The score definition and interval of the proportion of positive staining of tumor cells were follows: 0 point: negative expression; 1 point: the proportion of positive cells was <15%; 2 points: 15–50% positive cells; 3 points: positive cells ≥50%.
- The definition and interval of cell staining intensity were as follows: 0: no positive; 1point: light yellow; 2 points: pale brown; 3 points: brown.

The definition of RDAA positive in tumor tissue samples from patients was as followed: two experienced pathologists independently selected three different fields of view for each section to score RDAA immunohistochemical intensity and took the average value. The tissue samples from lung cancer patient was determined to be RDAA positive when the three IHC intensity scores of p-ALK Y1604, p-ALK Y1282/1283 and RNase1 of the tumor were all greater than 3 points by two doctors. If the scores of two pathologists were inconsistent, the discussion and evaluation were conducted by a third authoritative pathology.

### Duolink and immunofluorescence assay

Duolink assay was performed using the Duolink In Situ Red Starter Kit (Sigma DUO92101). Cells were fixed with 4% paraformaldehyde for 20 min, treated with 0.5% Triton X-100 for 20 min, and then blocked with 5% BSA for 1 h. After blocking, cells were incubated with ALK (1:100) or anti-RNase1 (1:200) antibody at 4 °C overnight and then subjected to Duolink assay according to the manufacturer’s protocol. The red spots were detected by fluorescence inverted microscope, and each red spot represents a cluster of protein-protein interaction. Nuclei were stained with DAPI (Invitrogen). For the immunofluorescence experiments, cells were fixed, blocked, and incubated with the primary antibodies under the same conditions of the Duolink assay. Cells were further incubated with a secondary antibody tagged with red or green fluorescein (Life Technologies) for 1 h at room temperature. All the fluorescence signals were observed under the Zeiss LSM 710 laser microscope.

### Cell viability assay

H1299, H1355, H322 and PC-9 Cells were cultured at 5 × 10^5^ cells per well in 6-well plates in 2 ml of medium and then treated with ALK inhibitors. At every experimental time point, 2 ml of 3-(4,5-Dimethylthiazol-2-yl)-2,5-diphenyltetrazolium bromide (MTT, 5 mg/ml) was added into each well, and then incubated at 37 °C for 2 h. After the removal of MTT solution, DMSO was added to each well to dissolve the water-insoluble purple precipitate. Absorbance at 595 nm was measured for each well.

### Colony formation assay

Indicated cells were cultured in soft agar medium and seeded at 5 × 10^2^ cells per well into 6-well plates. The cells were incubated for 2 weeks, and then treated with 4% paraformaldehyde for 20 min. After staining by 0.05% crystal violet for 15 min, the size and number of colonies were measured.

### Binding affinity assay

H1299 cells with wild-type ALK expression were used for binding affinity assay. ALK antibody (3 μg/ml) or normal mouse IgG (negative control) were diluted into 0.2 M sodium phosphate buffer (pH 6.5) and captured in ELISA 96-well (100 μl/well) plates overnight at 4 °C. Each well was washed with PBST buffer (PBS with 0.05% Tween 20), blocked with 200 μl 1% BSA solution (containing 0.05% Tween 20) for 3 h, and then rinsed with PBST buffer 3 times. H1299 cell lysate was added to each well (100 μl/well), incubated overnight at 4 °C, and then rinsed 3 times with PBST buffer. Recombinant RNase1 protein was diluted to different concentrations in lysis buffer and added to each well. After incubation overnight at 4 °C, each well was washed with 400 μl of 1 M sucrose three times, and then incubated with 100 μl of streptavidin-conjugated HRP (1:2000 in blocking buffer) at room temperature for 2 h. Each well was washed again with 400 μl 1 M sucrose three times, and then incubated with 100 μl TMB as a peroxidase substrate for 30 min at room temperature. A stop solution (50 μl) was added to each well to end the reactions. The OD was measured at 450 nm using a BioTek Synergy™ Neo multi-mode reader. The dissociation constant (Kd) was calculated by the above binding data using the GraphPad Prism software (Prism Software Inc.).

### Statistical analysis

All error bars are presented as mean standard deviation (SD). Statistical P values were calculated using a two-tailed, independent Student’s t-test, and P values less than 0.05 were considered significant. Significance of mean comparison is represented on the graphs as follow: *p < 0.05; **p < 0.01; NS, not significant. Kaplan-Meier survival analysis was used to compare patient survival using a log-rank test. The Pearson’s correlation coefficient (R) was used to evaluate a linear association between two variables of experimental data. An R value > 0.8 was considered as strong correlation.

## Supplementary information

Study Protocol

Supplementary Materials

Raw data-Main figure

Raw data-Supplementary

## Data Availability

A portion of data are available in the main text or the supplementary materials. However, additional data supporting the conclusions of the study should be obtained from the corresponding author upon reasonable request.
