# Supplementary material for: RNase1-driven ALK-activation is an oncogenic driver and therapeutic target in non-small cell lung cancer
Source: Signal Transduct Target Ther. 2025 Apr 18;10:124. doi: 10.1038/s41392-025-02206-x (PMC12006399; doi:10.1038/s41392-025-02206-x)
Supplement: Supplementary file 1 — Study Protocol [file 41392_2025_2206_MOESM1_ESM.pdf]

Statistical Analysis Plan (SAP) and Study Protocol

A phase II, single-arm, multicenter study of evaluating the efficacy and safety of ensartinib in patients with RDAA-positive non-small cell lung cancer

Principal Investigator	Lunxu Liu, MD, PhD<br>Professor of West China Hospital, Sichuan University<br>37 Guoxue Alley, Chengdu, Sichuan, China<br>E-mail: lunxu_liu@aliyun.com

Registry	Chinese Clinical Trial Registry
Registration number	ChiCTR2100054794

Author	Zhengyu Zha, MD, PhD<br>Professor of West China Hospital, Sichuan University<br>37 Guoxue Alley, Chengdu, Sichuan, China<br>E-mail: zyzha0924@wchscu.cn

Date of Registration	2021-12-27
Version	Draft 1.2

Abbreviations

AEs	Adverse events
ALT	Alanine aminotransferase
APTT	Activated partial thromboplastin time
AST	Aspartate aminotransferase
BUN	Blood urea nitrogen

CI	Confidence interval
CR	Complete remission
DCR	Disease Control Rate
DCR	Disease Control Rate
ECG	Electrocardiogram
EOT	End of Treatment
FAS	Full Analysis Set
GCP	Good Clinical Practice
GGT	Gamma-glutamyl transferase
HRQoL	Health-Related Quality of Life
ICF	Informed Consent Form
INR	International normalized ratio
iPFS	Intracranial Progression-Free Survival
ITT	Intention-to-treat
LCSS	Lung Cancer Symptom Scale
LDH	Lactate dehydrogenase
NMPA	National Medical Products Administration
NSCLC	Non-small cell lung cancer
ORR	Objective response rate
OS	Overall Survival
PD	Progression of disease
PFS	Progression-Free Survival
PR	Partial remission
PT	Prothrombin time
QoL	Quality of Life Assessment
RDAA	RNase1driven ALK activation
SAEs	Serious adverse events
SAP	Statistical Analysis Plan
SD	Stable disease

SOPs	Standard operating procedures
TTD	Time to Deterioration
TTP	Time to Progression
TTR	Time to Response

Table of contents

Introduction 6

Study Design 6

Overview of the study design 6

Flowchart of participant selecting process 7

Calculation of enrolled patients samples 7

Inclusion criteria 8

Exclusion criteria 9

Aims and Objectives 10

Primary objective 10

Secondary objective 10

Trial procedure 10

RDAA Detection Assay 14

Informed consent of patients 14

Outcomes 15

Primary outcome 15

Secondary outcomes 16

The Standard of Tumor Response to Therapy 16

Efficacy evaluation 17

Safety evaluation 17

Analyses 18

Statistical Analysis Datasets 18

1) Full Analysis Set 18

2) Per Protocol Set ... 18

3) Safety Set ... 18

4) Use of Analysis Set ... 18

Basic Statistical Methods ... 18

Quality Assurance ... 19

Quality Control and Assurance ... 19

Monitoring ... 19

Audits by the Sponsor and Inspections by Regulatory Authorities ... 19

Introduction

About 35-50% of the oncogenic drivers remain unknown or untargetable in patients with non-small cell lung cancer (NSCLC). Ensartinib, as a novel, potent, and highly selective second-generation ALK small molecule inhibitor, has been widely used in patients with ALK-rearranged positive NSCLC. Current study had identified RDAA as an oncogenic driver and a potentially effective therapeutic target in NSCLC. The aim of this study is to evaluate the efficacy and safety of ensartinib in patients with RDAA-positive NSCLC.

This statistical analysis plan (SAP) will give more detailed descriptions of the endpoints in the study and the corresponding analyses.

Study Design

Overview of the study design

This study is a single-arm, multicenter Phase II clinical trial, planning to enroll 40 patients with RDAA-positive NSCLC who have progressed after first-line treatment (including some patients who are unwilling or unable to tolerate standard first-line treatment). Among these, no fewer than 30 patients should be those receiving second-line treatment. Enrolled patients will take 225mg of ensartinib hydrochloride capsules once daily, either on an empty stomach or with food, until disease progression, intolerable toxicity, withdrawal decision by the investigator or subject, loss to follow-up, initiation of other anti-tumor treatments, or death. The primary endpoint is the objective response rate (ORR).

The clinical therapy study for RDAA+ patients received approval from the Biomedical Ethics Review Committee of West China Hospital of Sichuan University (Ethics Approval No. 2021-1669) and was duly registered with the Chinese Clinical Trial Registry (Registration No: ChiCTR2100054794, accessible at https://www.chictr.org.cn/showproj.html?proj=144966). Prior to undergoing treatment, patients were provided comprehensive information and subsequently signed an informed consent form, adhering strictly to the principles outlined in the Declaration of Helsinki.

Flowchart of participant selecting process

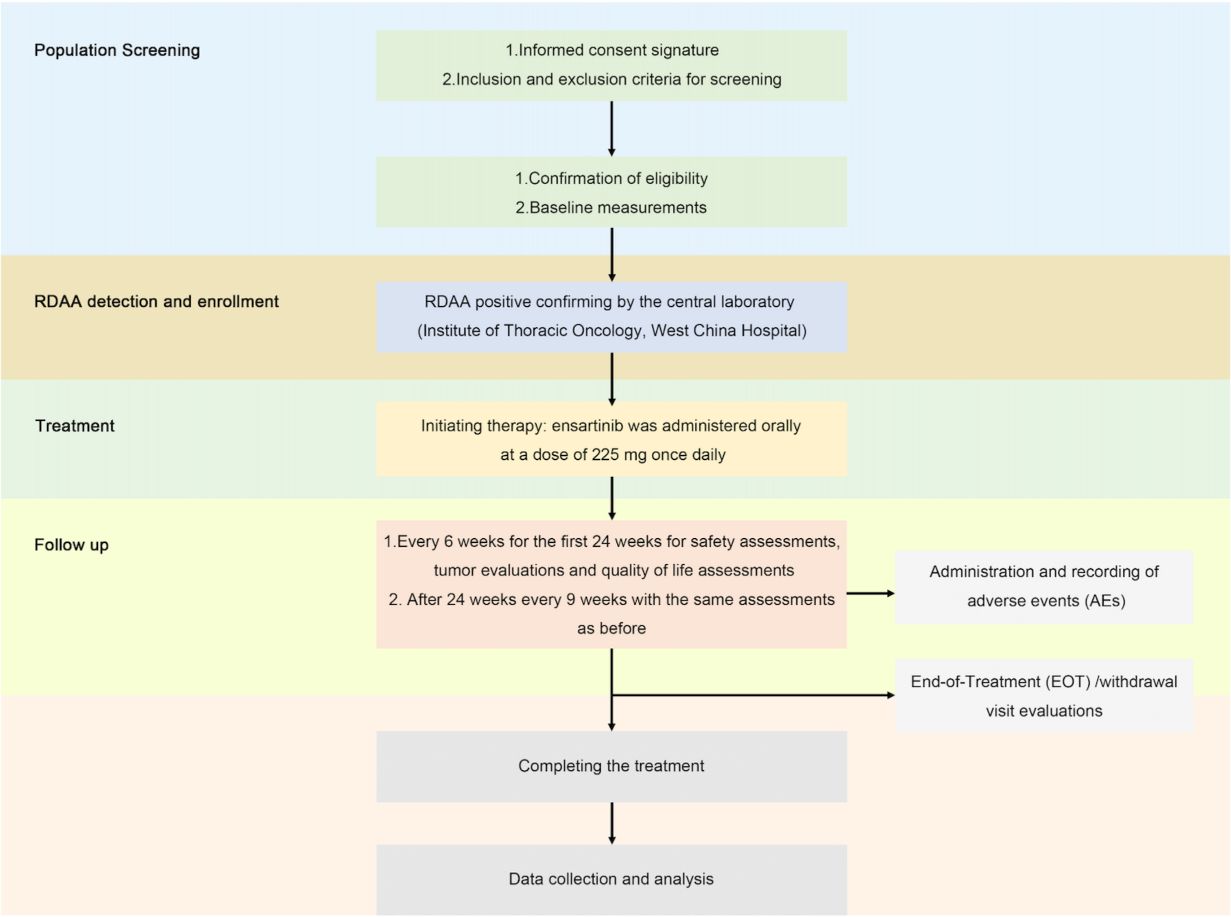

Calculation of enrolled patients samples

This study employed a single-arm, single-stage Phase II trial design to investigate the efficacy and safety of ensartinib as first-line treatment in RDAA-positive NSCLC patients in China, with objective response rate (ORR) as the primary endpoint. The sample size was calculated using a single-group target approach to determine the number of patients needed to achieve the minimum target ORR for ensartinib in RDAA-positive NSCLC patients who had progressed on or were intolerant to first-line treatment. At the time, there were no standard second-line treatments for RDAA-positive NSCLC internationally. In the PROFILE 1007 trial comparing crizotinib to chemotherapy as second-line treatment for ALK-positive NSCLC, the ORR was 20%(James E.et.al. Drugs, 2013). In the ASCEND-5 trial comparing ceritinib to chemotherapy (docetaxel or pemetrexed) as second-line treatment for ALK-positive NSCLC, investigators

reported an ORR of 6% (IRC 6.9%). Several domestic studies on second-line chemotherapy for NSCLC in China had shown ORRs ranging from 5.0% to 21.0%(Wei Hu et.al. Med Oncol 2015; Zhefeng Liu et.al. Med Oncol 2015; Rencui Quan et.al. Tumor Biol. 2016; Ning Tang et.al. OncoTargets and Therapy 2016). Based on these references, the minimum target ORR for the primary endpoint of this study was set at 30%. With a two-sided α of 0.05 and a power of 90%, the study required at least 32 patients. Considering a dropout rate of at least 20%, the study planned to enroll 40 patients. This study plans to enroll 40 participants (including up to 20% dropout), requiring at least 32 evaluable subjects to reach an assessable endpoint. If the number of subjects achieving an evaluable objective response rate (ORR) is less than 32, additional subjects will need to be recruited to ensure that the study maintains a power of 90%。

Inclusion criteria

1. Patients with locally advanced or metastatic non-small cell lung cancer confirmed by histology or cytology;
2. Previous genetic testing indicates that there is no mutation target that can be treated by targeted drugs;
3. Patients who have progressed after standard chemotherapy (including anti-angiogenesis therapy) or immunotherapy, or who cannot tolerate/refuse first-line chemotherapy;
4. Aged 18 to 70 years;
5. The Eastern Cooperative Oncology Group (ECOG) physical status score is 0-2;
6. Expected survival period >= 3 months;
7. Good organ function;
8. Patients with biopsy specimens confirmed to be RDAA positive;
9. Asymptomatic CNS metastases that do not require steroids or anticonvulsant treatment; symptomatic CNS metastases can be treated with radiotherapy first, and no progression after reexamination for >= 28 days can be included in the group; meningeal disease cannot be included in the group;
10. Patients must have RECIST1.1 standard measurable target lesions;
11. Drug-related toxic reactions other than alopecia should be alleviated to grade 2 or below (CTCAE 5.0 criteria);

12. Willing and able to comply with trial and follow-up procedures;
13. Be able to understand the nature of the trial and voluntarily sign a written informed consent.

Exclusion criteria

1. Patients who have used any ALK inhibitor in the past;
2. Patients currently receiving other anti-tumor systemic therapy;
3. Malignant tumors other than lung cancer have occurred in the past 3 years (excluding cured basal cell tumor of the skin, early gastrointestinal [GI] tumor resected under endoscopy, and carcinoma in situ of the cervix);
4. Patients who participated in clinical trials of other investigational drugs within 4 weeks before the first administration of ensartinib;
5. Major surgery or immunotherapy within 4 weeks before the first dose; patients who received radiation therapy within 2 weeks before the first dose;
6. Have received stem cell transplantation or organ transplantation;
7. Have serious cardiovascular disease;
8. Suffering from swallowing dysfunction, active gastrointestinal disease or other diseases that significantly affect the absorption, distribution, metabolism and excretion of ensartinib.
9. Active hepatitis B (serum HBV DNA >= 104 copies/ml [ie 20000IU/ml]), positive for hepatitis C virus antibody, positive for HIV antibody, and positive for Treponema pallidum antibody;
10. Past history of interstitial lung disease, history of drug-induced interstitial lung disease, history of radiation pneumonitis requiring steroid therapy, or any evidence of clinically active interstitial lung disease;
11. Females of childbearing age, pregnant or lactating females with positive serum pregnancy test 7 days before starting treatment, or male and female subjects who did not take effective contraceptive measures or plan to give birth during the entire treatment period and 3 months after the end of treatment;
12. Patients who are known to have allergic reactions to ensartinib or any excipients of ensartinib (microcrystalline cellulose, stearic acid, hydroxypropyl methylcellulose);

13. Patients who have used the following drugs within 14 days before the first dose or need to be combined with the following drugs during treatment: drugs that cause QTc prolongation and/or torsades de pointes risk; strong inhibitors or strong inducers of CYP3A;
14. Patients who are receiving warfarin or any other coumarin derivative anticoagulant;
15. Other severe, acute or chronic medical conditions, including uncontrolled diabetes or medical or mental illness or laboratory abnormalities, that may increase the risk associated with participating in the study, or may interfere with the interpretation of the study results, according to the investigator's opinion;
16. Other conditions deemed inappropriate by the investigator to participate in this trial.

Aims and Objectives

Primary objective

The primary objective is to evaluate the efficacy and safety of ensartinib in patients with RDAA-positive NSCLC.

Secondary objective

The secondary objective is to analyze the correlation between RNase1 detection in peripheral blood and RDAA detection results in tissue samples, as well as its relationship with clinical efficacy and safety; Explore the impact of other biomarkers or related factors that may exist on clinical efficacy and safety.

Trial procedure

1) Screening Period

Before the screening evaluation, subjects should be fully informed and sign an informed consent form. Screening examinations should be conducted within 28 days prior to administration. Subjects can only be enrolled to receive the study drug and a dosing diary card after the investigator confirms that they meet the inclusion criteria and do not meet the exclusion criteria. RDAA-positive screening will be uniformly completed by the central laboratory (Thoracic Oncology Research Institute, West China Hospital).

The screening items during the Screening Period were as follows:

Within 28 Days Prior to Drug Administration (-27 to 0 Days)

1. Informed Consent Form

2. Demographic Information

Collect basic demographic data including date of birth, age, gender, height, weight, and race/ethnicity.

3. Smoking History

Collect smoking history including duration and amount of smoking.

4. Medical History and Treatment History

Obtain a confirmed diagnosis based on cytology or tumor tissue pathology, history of biomarker testing, treatment history, other past medical history, and allergy history.

5. Genetic Testing

Patients must be confirmed through NGS testing with a written report as having no specific targetable mutations for currently marketed targeted therapies (including EGFR sensitizing mutations, KRAS G12C, BRAF, NTRK sensitizing mutations, or ALK, ROS1, RET rearrangements, or MET gene amplification and exon 14 skipping mutations). If this evidence is not available, NGS genetic testing must be performed on tumor tissue specimens obtained at the time of diagnosis or later to confirm the genetic status before the first administration of the drug. Patients are encouraged to obtain biopsy specimens before enrollment; biopsy tissue specimens will be tested by the West China Hospital Thoracic Oncology Laboratory Center and determined to be RDAA positive.

6. Tumor Evaluation

Perform imaging tumor evaluation (according to RECIST1.1 standards) within 28 days prior to the first administration, including enhanced CT of the chest, abdomen, and pelvis (plain CT can be used for subjects allergic to contrast agents), enhanced MRI of the brain (plain MRI can be used for subjects allergic to contrast agents), and bone scans (note: after the screening period, bone scans are only performed in patients with clinical symptoms; if bone scans show new abnormal locations, the disease location must be confirmed by X-ray or CT, MRI). These can be used for subject screening and as baseline values. To reduce harm to the subjects, imaging examinations that meet the above requirements and are performed before the subjects sign the informed consent form will not be repeated, and their results will be used directly for screening

and as baseline values.

7. Recording Adverse Events and Concomitant Medications

Within 7 Days Prior to Drug Administration (-6 to 0 Days), the Examination Results During This Period Will Be Used as Baseline Values

1. Quality of Life (QoL) Assessment

2. Physical Examination

Including skin and mucous membranes, lymph nodes, head and neck, chest, abdomen, musculoskeletal system, nervous system, and other parts.

3. Vital Signs

Including respiratory rate, axillary temperature (hospitals that do not routinely use axillary temperature can use other temperature measurement methods, but each center can only use one fixed method), heart rate, and seated blood pressure.

4. ECOG Performance Status Score

5. Complete Blood Count

Including white blood cell count, lymphocyte count, monocyte count, neutrophil count, eosinophil count, basophil count, red blood cell count, hemoglobin, hematocrit, platelet count.

6. Blood Biochemistry

Including alanine aminotransferase (ALT), aspartate aminotransferase (AST), total bilirubin, direct bilirubin, total protein, albumin, alkaline phosphatase, gamma-glutamyl transferase (GGT), blood urea nitrogen (BUN), creatinine, uric acid, total cholesterol, triglycerides, blood glucose, creatine kinase, lactate dehydrogenase (LDH), calcium, phosphorus, potassium, sodium, chloride, amylase.

7. Coagulation Function

Including prothrombin time (PT), activated partial thromboplastin time (APTT), international normalized ratio (INR).

8. Urinalysis

Including urine bilirubin, urobilinogen, ketones, urine protein, urine nitrite, urine glucose, urine pH, urine specific gravity, white blood cells, red blood cells.

9. Stool Examination + Occult Blood (Color, Occult Blood)

10. Infectious Disease Screening

Including hepatitis B surface antigen (HBSAg; if positive, HBV-DNA should be tested), HCV antibodies, HIV antibodies, and syphilis treponema antibodies.

11. Serum Pregnancy Test for Women of Childbearing Age

12. 12-Lead Electrocardiogram (ECG)

13. Biomarker Sample Collection

Collect 10mL of whole blood.

14. Recording Adverse Events and Concomitant Medications

15. Determining Eligibility According to Inclusion/Exclusion Criteria

Patients who are judged by the investigator to meet the inclusion criteria and not meet the exclusion criteria will be given the investigational drug and the subject medication diary card.

1) Treatment Period

The dosage of ensartinib is 225mg, taken once daily, either on an empty stomach or with food, until disease progression, intolerable toxicity, withdrawal decision by the investigator or subject, loss to follow-up, initiation of other anti-tumor treatments, or death. During the first 24 weeks of the trial, subjects will have a follow-up visit every 6 weeks. The visit will include safety assessments (physical examination, vital signs, ECOG performance status, complete blood count, blood biochemistry, coagulation function, urinalysis, fecal occult blood test, 12-lead ECG, hormone profile), tumor evaluation (contrast-enhanced CT of the chest/abdomen/pelvis, brain MRI), and quality of life assessment. After 24 weeks, subjects will have a follow-up visit every 9 weeks with the same assessments. When subjects first show disease response (CR and PR according to RECIST 1.1 criteria) during a visit, the evaluation result must be confirmed after 4 weeks.

2) End of Treatment/Withdrawal

An End of Treatment (EOT) visit should be conducted as soon as possible after the subject discontinues the study drug. For subjects who terminate or withdraw from treatment for reasons other than disease progression, a safety assessment should be performed as soon as possible, and tumor assessments should continue at the same frequency as during the treatment period until disease progression or initiation of other anti-tumor treatments. However, for subjects who

terminate treatment due to disease progression, only a safety assessment is required, and no further tumor assessments are necessary. If a subject discontinues treatment due to toxicity or other reasons at the last visit and does not continue to take the study drug thereafter, that visit will be considered the End of Treatment/Withdrawal visit.

3) Post-Treatment Follow-Up

For subjects who complete the trial or withdraw consent, all AEs and concomitant medications must be recorded up to 30 days after the last dose of the study drug. Any new AEs occurring within 30 days after the last dose must also be reported. All AEs must be followed up until resolution or stabilization, unless the investigator determines that the AE is due to the underlying disease and is unlikely to improve. For subjects who start other anti-tumor treatments, non-serious AEs deemed unrelated to the study drug by the investigator are no longer recorded.

4) Survival Follow-Up

For subjects who experience disease progression or start other anti-tumor treatments, no further safety or tumor assessments are performed. However, telephone follow-up every 12 weeks will continue to collect overall survival and subsequent treatment information until the subject's death or loss to follow-up.

RDAA Detection Assay

The antibodies of RDAA, the protocol for immunohistochemical staining and positive determination of RDAA were supported by Betta Pharmaceuticals Co., Ltd and Hangzhou Repugene Technology Co., Ltd.

The definition of RDAA positive in tumor tissue samples from patients was as followed: two experienced pathologists independently selected three different fields of view for each section to score RDAA immunohistochemical intensity and took the average value. The tissue samples from lung cancer patient was determined to be RDAA positive when the three IHC intensity scores of p-ALK Y1604, p-ALK Y1282/1283 and RNase1 of the tumor were all greater than 3 points by two doctors. If the scores of two pathologists were inconsistent, the discussion and evaluation were conducted by a third authoritative pathology.

Informed consent of patients

Information about this trial and instructions for subjects will be provided in the form of an

Informed Consent Form (ICF). The ICF used for this trial, as well as any amendments or additions to the ICF made during the trial, must be approved by both the sponsor and the ethics committee before being utilized. It is the investigator's responsibility to fully and comprehensively explain the nature, objectives, methods of the study, effects of the drug, reasonably expected benefits, potential side effects, and risks, as well as the rights of the subjects, and the risks and benefits they may bear. Subjects should be informed that their participation is completely voluntary and that they can withdraw from the trial at any time without any conditions and without incurring any penalties. The investigator must ensure that each subject fully understands the nature and objectives of the trial and the potential risks involved. The investigator or authorized personnel must obtain a signed and dated ICF from each subject before performing any trial-specific activities. The investigator will retain the original signed ICF from each subject as part of the original records. The investigator or authorized personnel must document the process of obtaining the ICF, the version of the ICF, and the date of signing in the subject's original records. Subjects should be promptly informed of any new information regarding the investigational drug.

Subjects will receive written notification and provide consent indicating that representatives from the sponsor, the ethics committee, or regulatory authorities may review their medical records to verify the information collected during the trial. All personal information involved in the review will be strictly confidential and comply with local data protection laws. The investigator and sponsor have the right, according to relevant regulations, to collect, review, retain, and statistically process the trial data of the subjects. All parties involved in the trial will ensure the protection of the personal data of the subjects: In any documents submitted to the sponsor, subjects will only be identified by their trial subject number and initials, and no identifying information such as names will be included in sponsor forms, reports, publications, or other types of disclosure documents. During the data transmission process, all parties involved in the trial will adhere to confidentiality standards to protect the personal data of the subjects.

Outcomes

Primary outcome

- Objective Response Rate (ORR) assessed by the Independent Review Committee (IRC) according to RECIST v1.1 criteria.

Secondary outcomes

- Objective Response Rate (ORR) assessed by the investigator.
- Disease Control Rate (DCR), Progression-Free Survival (PFS), Time to Response (TTR), Time to Progression (TTP), Duration of Response, Intracranial Objective Response Rate (according to RECIST v1.1 criteria), Intracranial Progression-Free Survival (according to RECIST v1.1 criteria), Time to Intracranial Progression (according to RECIST v1.1 criteria), Extracranial Objective Response Rate (according to RECIST v1.1 criteria), Extracranial Progression-Free Survival (according to RECIST v1.1 criteria), and Time to Extracranial Progression (according to RECIST v1.1 criteria), all assessed by both the IRC and the investigator.
- Overall Survival (OS).
- Time to Deterioration (TTD) measured by the EORTC C30/LC13 QoL questionnaire and the Lung Cancer Symptom Scale (LCSS) as reported by patients.
- Health-Related Quality of Life (HRQoL) changes from baseline as reported by patients using the EORTC C30/LC13 QoL questionnaire and the Lung Cancer Symptom Scale at each visit.

The Standard of Tumor Response to Therapy

The outcome of this study was the reduction of tumor after combination therapy, and the efficacy of the treatment regimen was determined according to the RECIST 1.1 criteria, which were as follows: complete remission (CR): disappearance of all target foci, and the short diameter of all pathological lymph nodes (both target and non-target) must be reduced to <10 mm; partial remission (PR): reduction of at least 30% in the sum diameter of the target foci compared to baseline; progression of disease (PD): relative increase in the sum of diameters of target lesions of at least 20%, referenced to the smallest of the sums of the diameters of all target lesions measured throughout the experimental study (or to baseline if baseline measurements are smallest), and in addition, an increase in the sum of the diameters of the diameters in absolute terms of at least 5 mm must be met (the presence of one or more new lesions is considered to be disease progression); stable disease (SD): a decrease in target lesion size that did not reach PR and an increase in target lesion size that did not reach PD, and in between, with reference to the smallest of the sums of the diameters of the target lesions studied.

Efficacy evaluation

According to the investigator's assessment and following the RECIST 1.1 efficacy evaluation guidelines, the determination of Complete Response (CR), Partial Response (PR), Stable Disease (SD), and Progressive Disease (PD) will be made. Evaluation times are every 6 weeks within the first 24 weeks of the trial, and every 9 weeks thereafter. When subjects first exhibit a response (CR and PR according to RECIST 1.1 criteria), the evaluation result must be confirmed after 4 weeks. Evaluation items include contrast-enhanced CT of the chest, abdomen, and pelvis (plain CT can be used for patients allergic to contrast agents), and contrast-enhanced MRI of the brain (plain MRI can be used for patients allergic to contrast agents). Other radiological evaluations (such as bone scans) will be conducted based on clinical indications. Evaluation of Efficacy Endpoints were as follows:a) Objective Response Rate (ORR) and Intracranial Objective Response Rate: Calculate the number and percentage of cases with Complete Response (CR) and Partial Response (PR), along with the 95% confidence interval (CI) for the percentage.b) Disease Control Rate (DCR) and Intracranial Disease Control Rate: Calculate the number and percentage of subjects with Complete Response (CR), Partial Response (PR), and Stable Disease (SD), along with the 95% confidence interval (CI) for the percentage.c) Time to Progression (TTP), Duration of Response (DOR), Progression-Free Survival (PFS), Intracranial Progression-Free Survival (iPFS), and Overall Survival (OS): Utilize the Kaplan-Meier method to estimate the median survival, first quartile (Q1), and third quartile (Q3), along with their 95% confidence intervals (CIs). Additionally, estimate the progression-free rate, progression-free survival rate, and overall survival rate, along with their respective 95% confidence intervals.

Safety evaluation

All subjects who receive the study drug will undergo safety evaluations. Safety assessments include vital signs (temperature, heart rate, respiration, blood pressure), physical examinations (general condition, skin, neck including thyroid, eyes, ears, nose, throat, chest, abdomen, back, lymph nodes, limbs, and neurological system), laboratory tests (complete blood count, urinalysis, stool analysis, hormone profile, blood biochemistry, and coagulation function), 12-lead ECG, and reporting of adverse events (AEs) and serious adverse events (SAEs). Safety follow-up will continue until 30 days after the subject discontinues the study drug.

Analyses

All outcomes will be presented using descriptive statistics; continuous variables will be presented using mean and standard deviation (SD) and categorical variables will be presented using counts and percentages. R (4.3.1, R Development Core Team, Austria) will be used for all statistical analysis. Statistical P values were calculated using a two-tailed, independent Student's t-test, and P values less than 0.05 were considered significant.

Statistical Analysis Datasets

1) Full Analysis Set

The Full Analysis Set (FAS), based on the intention-to-treat (ITT) principle, includes all enrolled subjects who meet important entry criteria without major protocol violations and have received at least one dose of investigational treatment. Subjects excluded from this analysis set need to be detailed with reasons provided.

2) Per Protocol Set

The Per Protocol Set (PPS) is a subset of the FAS that excludes subjects with major protocol violations, ensures drug compliance ⩾70%, and excludes those using prohibited medications. Subjects experiencing disease progression due to lack of efficacy of the investigational drug during the trial may be included in the PP dataset.

3) Safety Set

The Safety Set (SS) includes all enrolled subjects who have received at least one dose of investigational treatment and have safety assessment data after drug administration.

4) Use of Analysis Set

Baseline characteristics analysis utilizes the Full Analysis Set; efficacy analysis uses both the Full Analysis Set and the Per Protocol Set, with primary results based on the Full Analysis Set; safety analysis uses the Safety Set.

Basic Statistical Methods

Following trial protocol approval, statistical professionals collaborate with principal investigators to develop a statistical analysis plan. SAS statistical software version 9.4 or higher

is used for statistical analyses. Descriptive statistics for continuous data include counts, means, standard deviations, medians, maximums, and minimums; categorical or ordinal data are described using frequencies and percentages. Data used in general statistical tables will be presented in tabular form. All statistical tests employ two-sided tests with a significance level of $\alpha=0.05$. Missing data are not imputed.

Quality Assurance

Quality Control and Assurance

Before the initiation of the trial, investigators and research staff will undergo training on the trial protocol, Good Clinical Practice (GCP), and specific standard operating procedures (SOPs) related to this trial. During the conduct of the trial, investigators and research staff should strictly adhere to GCP, relevant guidelines issued by the NMPA (National Medical Products Administration), international norms, and the quality control and assurance policies of the clinical trial site. To ensure the protection of the rights of the subjects and adherence to the protocol and GCP, the sponsor will appoint monitors who will conduct regular monitoring activities.

Monitoring

Monitors appointed by the sponsor should conduct monitoring visits to the research site in accordance with GCP requirements and provide monitoring reports. During the trial or after its completion, monitors should regularly review the collected data against the original records to confirm their completeness, accuracy, authenticity, and reliability. Research staff should provide the necessary materials and cooperate with the monitors for their review. Monitors will verify with the investigators that the trial is being conducted according to the protocol and work with the investigators to resolve any issues arising during the trial.

Audits by the Sponsor and Inspections by Regulatory Authorities

To evaluate the quality of trial implementation, adherence to the standards of GCP, and applicable regulatory requirements, investigators, research institutions, and relevant units should allow audits by representatives appointed by the sponsor and inspections by regulatory authorities. Upon receiving notification of an inspection by regulatory authorities, the

investigator or research institution should promptly inform the sponsor. Investigators and research institutions should agree to allow auditors/inspectors direct access to the original research records. These personnel are bound by professional confidentiality and must not disclose personal identity or medical information. Investigators should make every effort to assist auditors and inspectors by providing all the necessary equipment, data, and documentation they require. Investigators should communicate the results and relevant information of the regulatory inspection to the sponsor in a timely manner. Investigators are required to take appropriate measures to address any issues identified during the audit or inspection as requested by the sponsor.
