# Supplementary material for: RNase1-driven ALK-activation is an oncogenic driver and therapeutic target in non-small cell lung cancer
Source: Signal Transduct Target Ther. 2025 Apr 18;10:124. doi: 10.1038/s41392-025-02206-x (PMC12006399; doi:10.1038/s41392-025-02206-x)
Supplement: Supplementary file 2 — Supplementary Materials [file 41392_2025_2206_MOESM2_ESM.pdf]

Supplementary Materials for

RNase1-driven ALK-activation is an oncogenic driver and therapeutic target in non-small cell lung cancer

Zhengyu Zha[1†], Chunxiao Liu[2†], Meisi Yan[3†], Cong Chen[1†], Cheng Yu[1], Yaohui Chen[1], Chenhao Zhou[4,5],Lu Li[6,7], Yi-Chuan Li[4], Hiro Yamaguchi[4], Leiguang Ye[2], Tong Liu[2], Ying-Nai Wang[4], Heng-Huan Lee[4], Wen-Hao Yang[4], Li-Chuan Chan[4], Baozhen Ke[4], Jennifer L. Hsu[4], Lieming Ding[8], Dong Ji[8], Peng Pan[8], Yiran Meng[9], Yue Pu[9], Lunxu Liu[1*] and Mien-Chie Hung[4*].

Correspondence to:

Lunxu Liu, Department of Thoracic Surgery and Institute of Thoracic Oncology, West China Hospital, University of Sichuan, No.37, Guoxue Valley, Wuhou District, Chengdu, Sichuan, 610041, China. E-mail: lunxu_liu@aliyun.com or liulunxu @wchscu.cn.

Mien-Chie Hung, Department of Molecular and Cellular Oncology, The University of Texas MD Anderson Cancer Center, Houston, TX 77030, USA. Tel: 886 04-22053366. Fax: 886 04-22060248. E-mail: mhung77030@gmail.com.

†Authors contributed to this work equally: Zhengyu Zha, Chunxiao Liu, Meisi Yan, Cong Chen

This PDF file includes:

Materials and Methods
Figures. S1 to S8
Tables S1 to S6

Materials and Methods

Cell culture.

NSCLC cell lines, HBE4-E6E7 cells, HEK293T cells and NIH3T3 cells were purchased from the ATCC. HBE4-E6E7 cells were maintained in keratinocyte serum free medium (K-SFM; Cat.17005-042, GIBCO) with 10% fetal bovine serum, 0.05 mg/ml bovine pituitary extract (BPE), 5 ng/ml epidermal growth factor (EGF) and 10 ng/ml cholera toxin. Other cells were maintained in Dulbecco's modified Eagle's medium/F-12 medium with 10% fetal bovine serum and antibiotics. All cell lines were validated by short tandem repeat (STR) DNA fingerprinting at MD Anderson Cancer Center (Houston, TX) and negative for mycoplasma infection.

RNases purification and activity assay.

Each RNase cDNA was cloned into a prokaryotic expression vector PSJ3 with N-terminal 6His tag. Plasmids expressing His-tagged RNase were used to transform E. coli BL21 cells. A single transformant was selected and cultured in 5-ml LB medium at 37 °C overnight. When OD reached > 1.2, cells were transferred to a 250-ml LB medium and incubated at 37 °C with shaking for about 2 hours. The cells were induced with 0.5 mM IPTG for 20–24 hours at 16 °C with shaking to express RNase when OD increased to between 0.5 and 0.6. After IPTG induction, cells were collected, lysed in lysis buffer (50 mM Tris-HCl, pH 7.5, 150 mM NaCl), sonicated, supernatant loaded onto the HisTrap column (GE) slowly. The column was washed with wash buffer containing 50 mM Tris-HCl, pH 7.5, 150 mM NaCl, 10 mM imidazole, and RNases eluted by elution buffer containing 50 mM Tris-HCl, pH 7.5, 150 mM NaCl, 300 mM imidazole. The RNase activity was detected by an Ambion RNase Alert Lab Test Kit (Life Technologies AM1964) following the manufacturer's protocol.

Stable cell lines. For ALK, RNase1, and EML4-ALK stable expression cell lines, the cDNA of each of the above genes was cloned into a modified pCDH-CMV-MCS-EF1-Puro vector

(CD510B-1; System Biosciences) or pCDH-CMV-MCS-EF1-Neo (CD514B-1; System Biosciences) vector. Site-specific mutant of RNase1 was generated by a Site-Directed Mutagenesis Kit (NEB E0554S). The lentiviral shRNAs were used to knock down ALK and RNase1 (Sigma). HEK293T cells were transfected with the indicated plasmids and packaging plasmids (VSV-G and dvpr) to generate viruses. H1299, H322, H1355 and NIH3T3 cells were infected with above viruses for 6–8 hours with polybrene (10 g/ml) and then subjected to puromycin (2 g/ml) or G418 (2000 g/ml) selection to generate stable transfectants. Lipofectamine 2000 (Life Technologies) was used for HEK293T transfection.

Antibodies and reagents.

Antibodies against RNase1 (Sigma HPA001140), ALK (Cell Signaling 3633), phosphorylated ALK (Tyr1604; Cell Signaling 3341S), phosphorylated ERK1/2 (Thr202/Tyr204; Cell Signaling 9101), ERK1/2 (Cell Signaling 9102), STAT3 (Cell Signaling 9132), phosphorylated STAT 3 (Cell Signaling 9145S), PD-L1 (Cell Signaling 13684s), and tubulin (Sigma B-5-1-2) were used for immunoblotting, immunohistochemistry, immunofluorescence, and Duolink assays. PE anti-mouse CD274 (B7-H1, PD-L1) Antibody (Biolegend 124307) was used for flow cytometry. For ALK inhibitor crizotinib (PF-02341066), ceritinib (S7083) and alectinib (CH5424802) were purchased from Selleckchem. The ELISA Kit for human RNase1 (SEA297Hu) and FAM150 (MBS9328956) were used to measure their plasma concentration. Plasma (5 μl) from patients or mice were added to 195 μl phosphate-buffered saline (PBS; 1:40 dilution) and then subjected to ELISA assay based on the manufacturer's protocol.

Animal experiments.

All animal experiments were performed under the guidelines and protocols approved by the MD Anderson Institutional Animal Care and Use Committee (IACUC). The nude male mice were

purchased from Jackson Laboratory. In in vivo tumorigenesis assay, 1×10^6 indicated stable NIH-3T3 cells were subcutaneously injected into 6-week-old male nude mice (for NIH3T3 control, ALK, RNase1 and RDAA group, n = 8/group; for EML4-ALK group, n = 5/group). Tumor volumes were monitored using external calipers and calculated by (length × width^2)/2. In the subcutaneous model, 6 groups of 48 nude mice were subcutaneously injected with 2×10^5 stable H1299-control, H1299-RDAA or H1299-EML4-ALK cells. Tumor-bearing mice in both groups were further placed in two groups (n = 8/group) for treatment with ceritinib (25 mg/kg/day), crizotinib (50 mg/kg/day), or vehicle control. Drugs were administrated via oral every day for 2 weeks. Tumor volume was measured every 3 days. In the orthotopic model, subcutaneous H1299-Control and H1299-RDAA tumors were cut into cubes (about 1 mm3) and then were transplanted into the lung tissue of nude mice under isoflurane anesthetized. Mice were given ceritinib (25 mg/kg/day) or matched vehicle control at 7 days after inoculation, and continuous for two weeks. The orthotopic tumors were evaluated by micro-CT scanner (Fig. 3f). At the endpoint of experiments, mice were killed by CO2 exposure and cervical dislocation according to the guidelines.

Clinical samples study.

Paired plasma samples from the 48 patients with NSCLC were analyzed by ELISA to measure RNase1 plasma concentration (SEA297Hu). For immunohistochemistry, paraffin-embedded archived lung cancer tissue sections were submerged in xylene to remove the paraffin, and rehydrated with an ethanol gradient. H2O2 (3%) was used to block endogenous peroxidase activity, and antigen retrieval was carried out by incubating sections in sodium citrate with microwave heating. Slides were cooled to room temperature for 1 hour. The sections were blocked with 5% goat serum (Beyotime Biotechnology) and then incubated overnight with ALK (1:50, CST) or p-

ALK (1:100, CST) antibody at 4 °C. After washing with PBS, sections were incubated with HRP-conjugated secondary antibody (Abcam) at room temperature for 1 hour. Sections were developed for visualization using diaminobenzene. Study of tissue staining and plasma samples from the patients was approved by the Ethics Committee of Cancer Hospital Affiliated to Harbin Medical University (IRB number: KY2019-18).

Clinical study for RDAA+ patients.

The clinical therapy study for RDAA+ patients received approval from the Biomedical Ethics Review Committee of West China Hospital of Sichuan University (Ethics Approval No. 2021-1669) and was duly registered with the Chinese Clinical Trial Registry (Registration No: ChiCTR2100054794, accessible at https://www.chictr.org.cn/showproj.html?proj=144966, Statistical Analysis Plan (SAP) and Study Protocol of the clinical study was shown in Supplementary Materials). Prior to undergoing treatment, patients were provided comprehensive information and subsequently signed an informed consent form, adhering strictly to the principles outlined in the Declaration of Helsinki.

The eligibility criteria for patient inclusion were meticulously defined as follows:

a) Age requirement of ≥18 years, encompassing both genders. b) Confirmed histological or cytological diagnosis of locally advanced or metastatic non-small cell lung cancer (NSCLC). c) Molecular testing (Next-Generation Sequencing, NGS) ensuring the absence of known EGFR, KRAS, BRAF sensitive mutations, NTRK, ALK, ROS1, RET rearrangements, or MET gene alterations (amplification and exon 14 jump mutations) in tumor samples. d) Expression of PD-L1 below 20%, validated through the VENTANA PD-L1(SP263) assay. e) RDAA-positive confirmation of biopsy tissue specimens through laboratory testing conducted at West China Hospital. f) Documented disease progression post-standard treatment (according to RECIST 1.1

criteria) or demonstrated reluctance or intolerance to first-line therapy. g) Eastern Cooperative Oncology Group (ECOG) performance status score of 0-2. h) Anticipated survival of ≥3 months. i) Satisfactory physiological parameters and organ function. J) Asymptomatic central nervous system (CNS) involvement not necessitating steroid or anticonvulsant therapy for metastasis. k) Presence of a measurable lesion as per RECIST 1.1 criteria. l) Resolution of drug-related toxicity reactions, excluding alopecia, to grade 2 or lower (according to CTCAE 4.03 criteria). m)Demonstrated willingness and capacity to adhere to trial protocols and follow-up procedures, coupled with a comprehensive understanding of the trial's nature, as evidenced by voluntary signature on the written informed consent form.

The patient exclusion criteria were rigorously outlined as follows:

a) Confirmation of known oncogenic driver mutations such as EGFR, KRAS, BRAF, NTRK, ALK, ROS1, RET rearrangement, or MET gene alterations (amplification and exon 14 jump mutations), or PD-L1 expression ≥20%. b) Pathological findings suggestive of mixed tumors. c) Concurrent administration of other systemic anti-tumor therapies. d) History of malignancy other than lung cancer within the past 3 years (excluding cured cutaneous basal cell tumors, endoscopically resected early gastrointestinal tumors, and cervical carcinoma in situ). e) Participation in another investigational drug clinical trial within 4 weeks or undergoing major surgery (including stem cell transplantation or organ transplantation, immunotherapy, or radiation therapy) prior to the initial drug dose. f) Presence of severe cardiovascular disease or cardiovascular abnormalities within 6 months preceding the first dose. g) Significant medical conditions affecting drug absorption, distribution, metabolism, and excretion (e.g., swallowing dysfunction, active gastrointestinal disorders, etc.). h) Positive serology for active hepatitis B, hepatitis C viruses, HIV, and syphilis. i) History of prior interstitial lung disease, drug-induced interstitial lung disease, radiation

pneumonitis requiring steroid therapy, or evidence of clinically active interstitial lung disease. j) Women of childbearing potential or breastfeeding females with a positive serum pregnancy test within 7 days before treatment initiation, or both males and females not using effective contraception or planning to conceive during treatment and up to 3 months post-treatment completion. k) Known history of anaphylactic reaction to ensartinib or any of its excipients. l) Use of medications within 14 days prior to the first dose or requiring a combination of medications associated with a risk of QTc prolongation and/or torsades de pointes, or a strong CYP3A inhibitor or inducer during treatment. m) Anticoagulant therapy with warfarin or any other coumarin derivative. n) Presence of severe acute or chronic medical conditions, deemed by the investigator to increase the risk associated with study participation or interfere with the interpretation of study results.

Clinical trial procedure

Before undergoing screening assessment, patients must receive full information and sign an informed consent form. The screening phase must be completed within 28 days prior to dosing. Enrollment in the study was contingent upon the investigator confirming adherence to inclusion criteria and absence of exclusion criteria. Subsequently, patients were provided with the study drug and a dosing diary card. RDAA-positive screening was uniformly conducted by the central laboratory (Institute of Thoracic Oncology, West China Hospital).

Upon initiating therapy, ensartinib was administered orally at a dose of 225 mg once daily until disease progression, development of intolerable toxicity, decision by the investigator or patient to withdraw, loss to follow-up, initiation of other antineoplastic therapy, or death. Patients were assessed every 6 weeks for the first 24 weeks of the trial for safety assessments (including physical examination, vital signs, ECOG performance status score, routine blood tests, blood biochemistry,

coagulation studies, routine urine analysis, routine stool examination with occult blood test, 12-lead electrocardiogram, and hormone panel), tumor evaluations (thoracic/abdominal/pelvic contrast-enhanced CT scans, cranial MRI), and quality of life assessments. After 24 weeks, visits occured every 9 weeks with the same assessments as before. Patients experiencing their first remission or disease control (complete response, partial response, or stable disease according to RECIST 1.1 criteria) at a visit will undergo a review 4 weeks later to confirm the evaluation results. For patients completing the trial or withdrawing informed consent, all adverse events (AEs) and drug combinations must be documented up to 30 days after the last trial dose, and any new AEs occurring within 30 days of the last trial dose must be reported. All AEs must be monitored until resolved or stabilized unless deemed unrelated to the trial medication and attributable to the primary disease. Non-severe AEs, determined by the investigator to be unrelated to the trial medication, will not be recorded for patients who initiate other antineoplastic therapy.

End-of-Treatment (EOT) visit evaluations were conducted promptly after the patient discontinues the trial drug. Any patient who terminated treatment or withdrew from treatment for reasons other than disease progression should undergo a safety assessment promptly, while continuing to undergo tumor assessments at the same frequency as during the treatment period until disease progression occurs or other antineoplastic therapy was initiated. However, patients whose treatment termination was due to disease progression should only undergo safety assessment, and no further tumor assessments should be conducted. If a patient discontinues treatment at the final visit due to toxicity or other reasons and did not continue to take the test drug thereafter, the visit was considered an end-of-treatment/withdrawal visit.

Survival Follow-up and Efficacy Assessment:

Patients who experienced disease progression or initiated other anti-tumor therapies were no longer subject to safety and tumor assessments but continued to be followed up via telephone every 12 weeks. During these follow-up calls, information regarding overall survival and subsequent treatment was collected until death or loss to follow-up. The outcome of this study was the reduction of tumor size after combination therapy, and the efficacy of the treatment regimen was determined according to the RECIST 1.1 criteria, which were as follows: complete remission (CR): disappearance of all target foci, and the short diameter of all pathological lymph nodes (both target and non-target) must be reduced to <10 mm; partial remission (PR): reduction of at least 30% in the sum diameter of the target foci compared to baseline; progression of disease (PD): relative increase in the sum of diameters of target lesions of at least 20%, referenced to the smallest of the sums of the diameters of all target lesions measured throughout the experimental study (or to baseline if baseline measurements are smallest), and in addition, an increase in the sum of the diameters of the diameters in absolute terms of at least 5 mm must be met (the presence of one or more new lesions is considered to be disease progression); stable disease (SD): a decrease in target lesion size that did not reach PR and an increase in target lesion size that did not reach PD, and in between, with reference to the smallest of the sums of the diameters of the target lesions studied.

Patients #05-#08 in this study received ensartinib treatment(225 mg /day). Simultaneously, patient #09 also met the inclusion criteria and voluntarily signed the consent form to receive ensartinib treatment. However, patient #09 resided in Zhejiang Province, which was 1853.3 kilometers away from our study center located in Chengdu. Due to the unwillingness to travel such a long distance for treatment and follow-up, the patient was not enrolled in the clinical study. Instead, the patient was treated as an observational case and received the same ensartinib treatment and follow-up

regimen at the local hospital and voluntarily signed informed consent to share the clinical data collected during the treatment period.

Additionally, during the period shortly before the start of this clinical study, we collaborated with the Department of Thoracic Surgery at Harbin Medical University Cancer Hospital to screen and identify 5 cases of RDAA-positive NSCLC patients. Four of them (Patient #01-#04) voluntarily signed the consent forms and began receiving crizotinib treatment (250–500 mg/day). The follow-up procedures for these 4 patients after treatment were consistent with those of patients #05-#08 and maintained for 3 years.

RDAA detection assay.

The antibodies of RDAA, the protocol for immunohistochemical staining and positive determination of RDAA were supported by Betta Pharmaceuticals Co., Ltd and Hangzhou Repugene Technology Co., Ltd.

Immunohistochemical staining procedures by using RDAA antibodies:

The primary antibodies used in this study during the immunohistochemical staining were all murine monoclonal antibodies. After deparaffinizing and rehydrating, the paraffin sections were repaired with high-temperature microwave (EDTA antigen repair solution: pH 8.0 (Beijing Zhong Shan -Golden Bridge Biological Technology Co., Ltd., cat: ZLI-9067, China), heated for 8 minutes each, two times), blocked with 3%H2O2 (20-30 minutes) and 10% goat serum (30 minutes), respectively. Then 100ul diluted p-ALK Y1604 (dilution ratio 1:500, antibody concentration 0.5mg/ml), p-ALK Y1282/1283 (1:250, Antibody stock concentration 0.5mg/ml),RNase1 (dilution ratio 1:500, antibody stock concentration 0.5mg/ml) were incubated to cover the surface of the tissue sections overnight at 4 ° C(The production steps of RDAA-related antibodies have been described in the previous article and will not be repeated in this article). Subsequent overnight

sections were incubated with mouse secondary antibody diluted in PBS (dilution ratio: 1:250, cat: 115-035-003, Jacson, United States) at room temperature for 40 minutes and then stained with SignalStain® DAB Substrate Kit (Cat. 8059S, CST, USA) (color reaction time: p-ALK Y1604: 40 seconds, p-ALK Y1282/1283:20 seconds, RNase1:40 seconds). After DAB color development, the slices were nucleated with hematoxylin, separated with hydrochloric acid alcohol, returned to blue with ammonia water, dehydrated with absolute ethanol, and then sealed.

Immunohistochemical staining scoring rules of RDAA antibodies:

The positive expression intensity of RDAA was evaluated in tissue sections with tumor area greater than 70% in the field of high-power microscope (10x eyepiece + 40x objective). Whether a lung cancer tumor section was judged to be RDAA positive or not was comprehensively determined according to IHC intensity scores of the three antibodies： p-ALK Y1604, p-ALK Y1282/1283 and RNase1.The score formula was as follows: score of the proportion of positive staining cells of tumor cells * Staining intensity score.

a) The score definition and interval of the proportion of positive staining of tumor cells were follows: 0 point: negative expression; 1 point: the proportion of positive cells was < 15%; 2 points: 15-50% positive cells; 3 points: positive cells ≥50%.

b) The definition and interval of cell staining intensity were as follows: 0: no positive; 1point: light yellow; 2 points: pale brown; 3 points: brown.

The definition of RDAA positive in tumor tissue samples from patients was as followed: two experienced pathologists independently selected three different fields of view for each section to score RDAA immunohistochemical intensity and took the average value. The tissue samples from lung cancer patient was determined to be RDAA positive when the three IHC intensity scores of p-ALK Y1604, p-ALK Y1282/1283 and RNase1 of the tumor were all greater than 3 points by

two doctors. If the scores of two pathologists were inconsistent, the discussion and evaluation were conducted by a third authoritative pathology.

Duolink and immunofluorescence assay.

Duolink assay was performed using the Duolink In Situ Red Starter Kit (Sigma DUO92101). Cells were fixed with 4% paraformaldehyde for 20 min, treated with 0.5% Triton X-100 for 20 min, and then blocked with 5% BSA for 1 hour. After blocking, cells were incubated with ALK (1:100) or anti-RNase1 (1:200) antibody at 4°C overnight and then subjected to Duolink assay according to the manufacturer's protocol. The red spots were detected by fluorescence inverted microscope, and each red spot represents a cluster of protein-protein interaction. Nuclei were stained with DAPI (Invitrogen). For the immunofluorescence experiments, cells were fixed, blocked, and incubated with the primary antibodies under the same conditions of the Duolink assay. Cells were further incubated with a secondary antibody tagged with red or green fluorescein (Life Technologies) for 1 hour at room temperature. All the fluorescence signals were observed under the Zeiss LSM 710 laser microscope.

Cell viability assay.

H1299, H1355, H322 and PC-9 Cells were cultured at 5×10^5 cells per well in 6-well plates in 2 ml of medium and then treated with ALK inhibitors. At every experimental time point, 2 ml of 3-(4,5-Dimethylthiazol-2-yl)-2,5-diphenyltetrazolium bromide (MTT, 5 mg/ml) was added into each well, and then incubated at 37 °C for 2 hours. After the removal of MTT solution, DMSO was added to each well to dissolve the water-insoluble purple precipitate. Absorbance at 595 nm was measured for each well.

Colony formation assay.

Indicated cells were cultured in soft agar medium and seeded at 5×10^2 cells per well into 6-well plates. The cells were incubated for 2 weeks, and then treated with 4% paraformaldehyde for 20 min. After staining by 0.05% crystal violet for 15 min, the size and number of colonies were measured.

Binding affinity assay.

H1299 cells with wild-type ALK expression were used for binding affinity assay. ALK antibody (3 µg/ml) or normal mouse IgG (negative control) were diluted into 0.2 M sodium phosphate buffer (pH 6.5) and captured in ELISA 96-well (100 µl/well) plates overnight at 4°C. Each well was washed with PBST buffer (PBS with 0.05% Tween 20), blocked with 200 µl 1% BSA solution (containing 0.05% Tween 20) for 3 hours, and then rinsed with PBST buffer 3 times. H1299 cell lysate was added to each well (100 µl/well), incubated overnight at 4°C, and then rinsed 3 times with PBST buffer. Recombinant RNase1 protein was diluted to different concentrations in lysis buffer and added to each well. After incubation overnight at 4°C, each well was washed with 400 µl of 1M sucrose three times, and then incubated with 100 µl of streptavidin-conjugated HRP (1:2,000 in blocking buffer) at room temperature for 2 hours. Each well was washed again with 400 µl 1M sucrose three times, and then incubated with 100 µl TMB as a peroxidase substrate for 30 min at room temperature. A stop solution (50 µl) was added to each well to end the reactions. The OD was measured at 450 nm using a BioTek Synergy™ Neo multi-mode reader. The dissociation constant (Kd) was calculated by the above binding data using the GraphPad Prism software (Prism Software Inc.).

Statistical analysis. All error bars are presented as mean standard deviation (SD). Statistical P values were calculated using a two-tailed, independent Student's t-test, and P values less than 0.05 were considered significant. Significance of mean comparison is represented on the graphs as

follow: *$p < 0.05$; **$p < 0.01$; NS, not significant. Kaplan-Meier survival analysis was used to compare patient survival using a log-rank test. The Pearson's correlation coefficient (R) was used to evaluate a linear association between two variables of experimental data. An R value > 0.8 was considered as strong correlation.

Figure. S1.

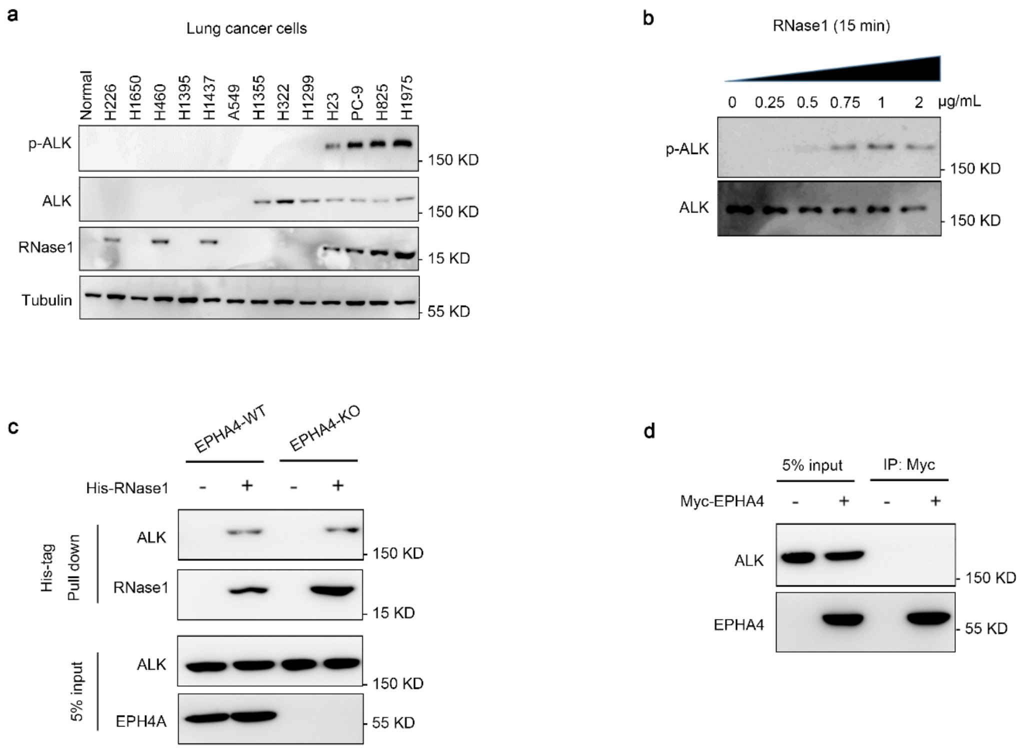

Figure.S1 (a) Western blotting analysis of ALK, RNase1, and ALK Y1604 phosphorylation level in 13 human lung cancer cell lines. An immortalized human bronchial cell line HBE4-E6E7 was used as normal cell control. (**b**) Dose-dependent assay of ALK activation by RNase1 stimulation. H1299 cells were treated by different concentrations of RNase1 for 15 min and then subjected to Western blotting. (**c**) His-tag pull down assay and Western blotting analysis of RNase1 and ALK interaction in EPHA4 wildtype (WT) or EPHA4 knockout (KO) cells. (**d**) co-IP of EPHA4 and ALK in H1299 cells. Plasmids expressed Myc-tagged EPHA4 were transfected into H1299 cells and the cell lysate were used for co-IP.

Figure. S2.

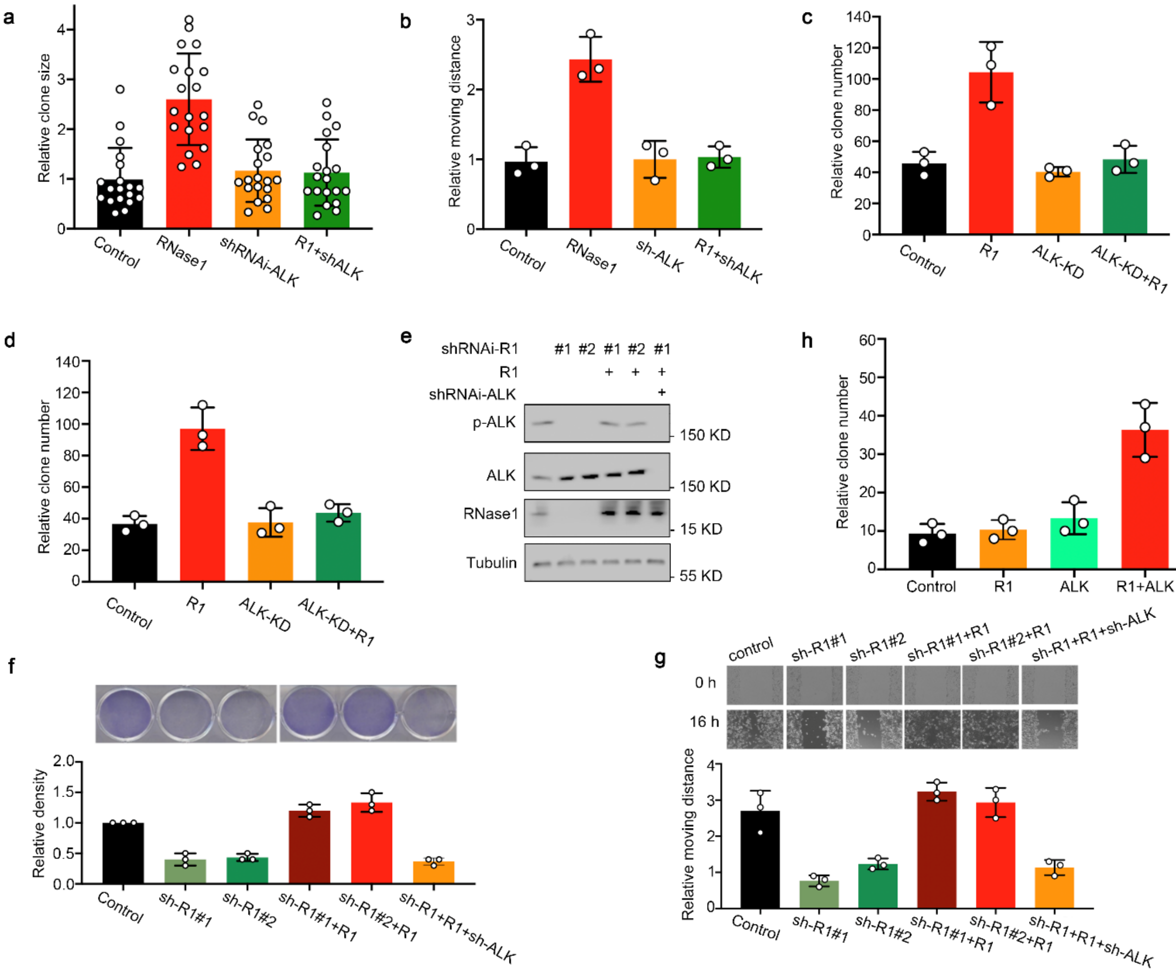

Figure.S2 (a) Quantification of cell clone size for H1299 cells with or without RNase1/ALK expression. The clone size was determined from three replicates. **(b)** Quantification of cell moving distance for experiments as describe in Figure 2e. **(c, d)** Quantification of cell clone number for H322 and H1355 cells with or without RNase1/ALK expression. The clone numbers were calculated from three replicates. **(e)** Western blotting for PC-9 cells with or without RNase1/ALK knockdown or RNase1 rescue. **(f)** MTT for cell proliferation in PC-9 cells as described in Figure S2e. Cells were stained by MTT and quantified. **(g)** Wound healing assay for PC-9 cells. Cell moving distance was measured and quantified. **(h)** Quantification of cell clone number for NIH3T3 cells with or without RNase1/ALK expression. The clone numbers were calculated from three replicates.

Figure. S3.

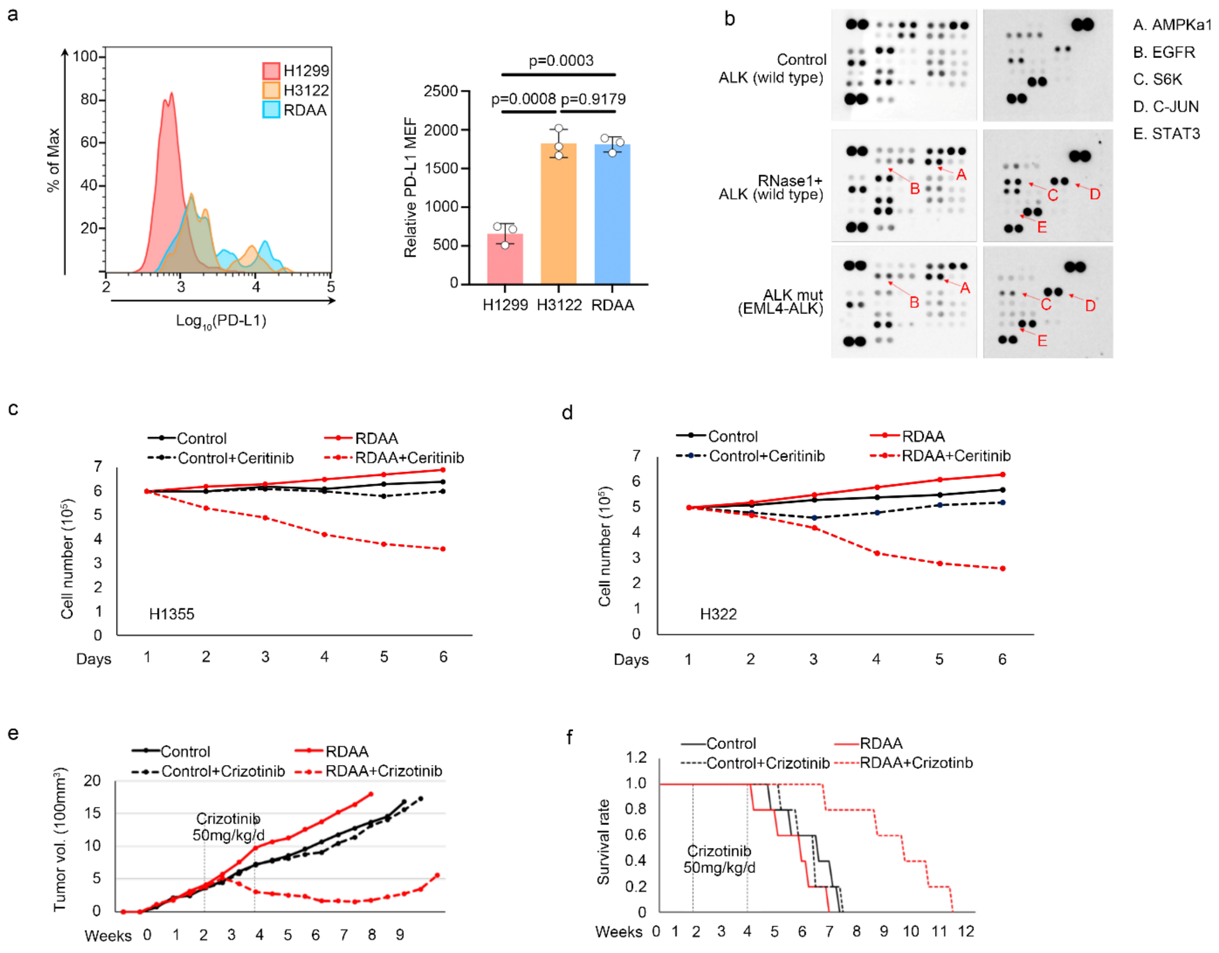

Figure.S3 (a) Flow cytometry detected tumor PD-L1 expression of H1299, H3122, and RDAA. H1299 cells were used as a negative control, while H3122 cell line served as a ALK rearrangement control. *****p* < 0.0001, Student's *t*-test. **(b)** Phosphorylation kinase antibody array for control, RDAA and *ALK*-rearranged cells (H2228). Red arrow indicates the upregulated signaling compared with control cells; Quantification of cell number for H1355 **(c)** and H322 **(d)** cells with (RDAA) or without (control) RNase1 expression under ceritinib treatment. The cell number was calculated from three replicates. **(e)** Mice received the indicate H1299 control cells or RDAA cells by subcutaneous injection, and when tumors reached about 500 mm^3, ALK inhibitor crizotinib (50 mg/kg/d) was administered beginning on day 14 for 2 weeks. Tumor size was measured every 3 days. N = 5. **(f)** Overall survival curve of mice in **(e)** starting from the day of cell injection.

Figure. S4.

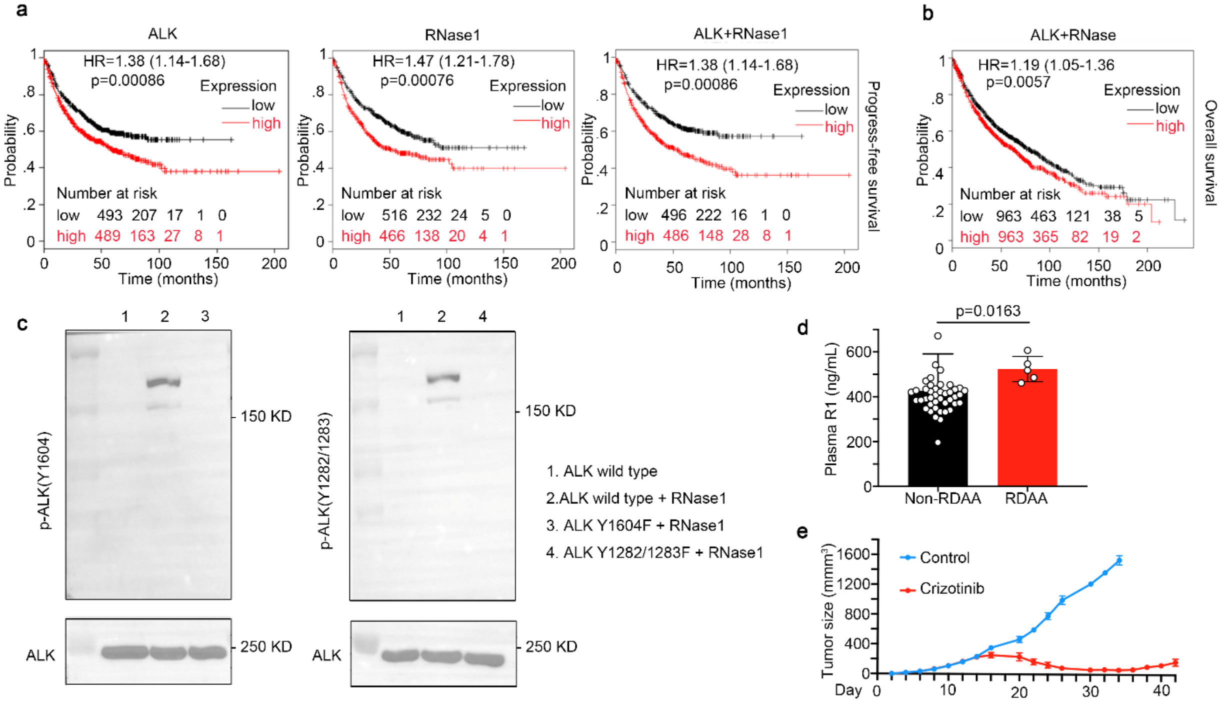

Figure.S4 (a, b) Progression-free survival (**a**) and overall survival (**b**) of lung cancer patients with high or low level RNase1/ALK. (TCGA database, kmplot.com). **(c)** Western blotting analysis of p-ALK Y1604 and p-ALK Y1282/1283 monoclonal antibodies in cell expression wild type or mutate ALK with/without RNase1 treatment. **(d)** Plasma RNase1 concentration in RDAA group and Non-RDAA lung cancer patients, *$p < 0.05$. **(e)** Tumor size of RDAA positive PDX mice with or without Crizotinib treatment. Crizotinib treatment started at day 12 and ended at day 26.

Figure. S5.

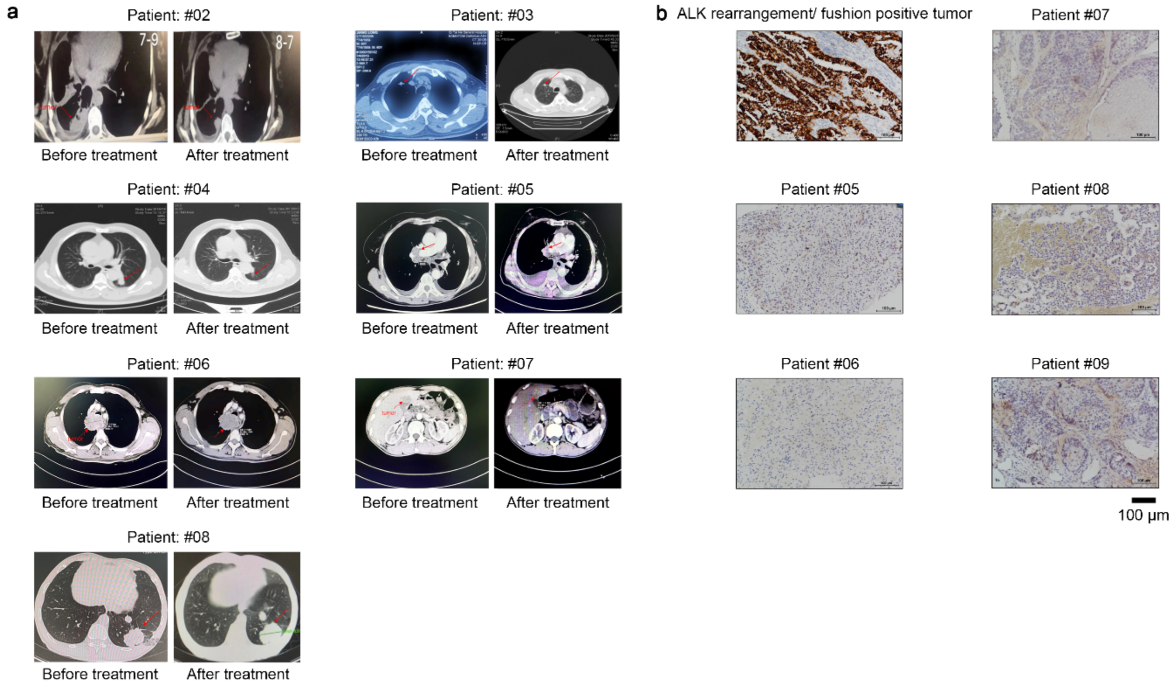

Figure.S5 (a) Representative clinical CT scan images of RDAA patients before and after crizotinib treatment. Red arrow points to tumor position. Patient #02: PR (partial response; response), #03: SD (stable disease), #04: PR (partial response; response), #05: SD (stable disease), #06: SD (stable disease;), #07: PR (partial response; response), #08: PD (progressive disease; no response) according to Response Evaluation Criteria in Solid Tumors [RECIST]. **(b)** ALK (D5F3) VENTANA immunohistochemical test confirmed no ALK rearrangement/fusion mutation among enrolled patients in our clinical therapy study.

Figure. S6.

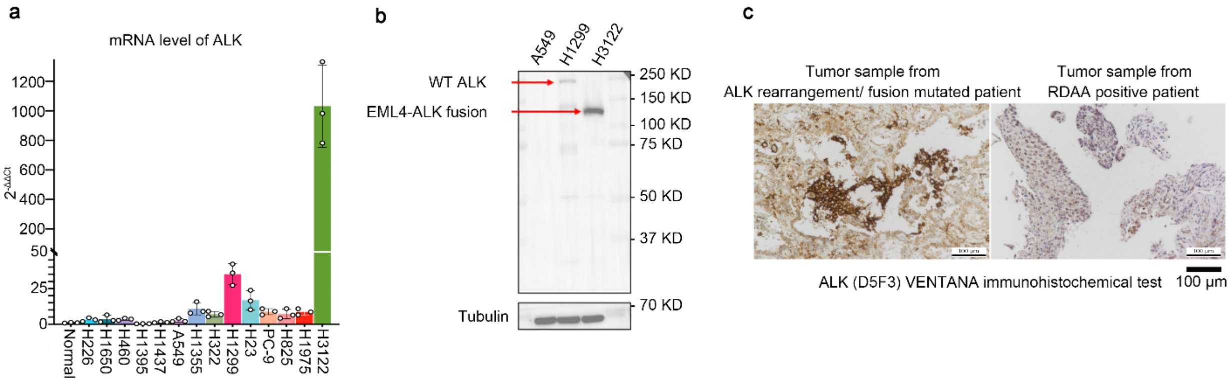

Figure.S6 (a) mRNA level analysis of ALK expression level in 14 human lung cancer cell lines. An immortalized human bronchial cell line HBE4-E6E7 was used as normal cell control. H3122 was used as ALK rearrangement control. **(b)**Western blot comparing ALK expression in H3122 and H1299 cells (A549 cells were used as an ALK expression-negative control).**(c)** Comparison of ALK expression in patient samples using ALK VENTANA IHC test.

Figure. S7.

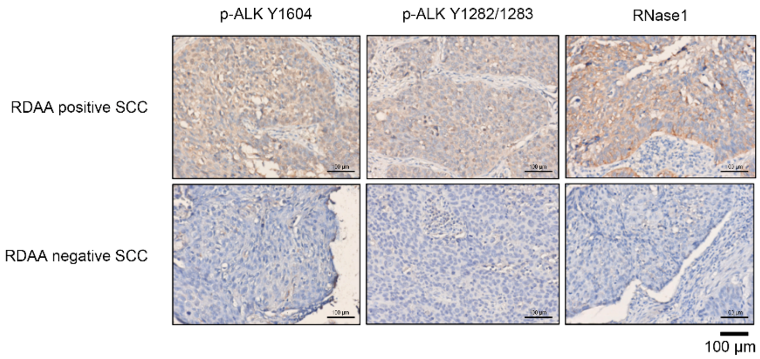

Figure.S7 IHC staining of RNase1 expression and ALK phosphorylation levels in human squamous cell carcinoma (SCC) tissues. ALK p-Y1604, ALK p-Y1282/1283 and RNase1 specific antibodies was used for IHC staining. Representative RDAA positive and negative images shown.

Figure. S8.

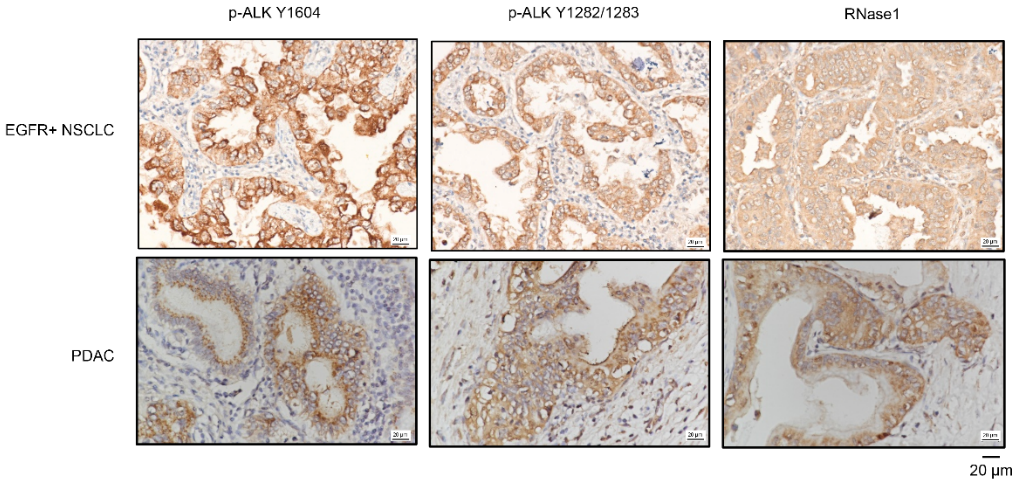

Figure.S8 IHC staining of RNase1 expression and ALK phosphorylation levels in human EGFR mutation positive NSCLC tissues and PDAC tissues. ALK p-Y1604, ALK p-Y1282/1283 and RNase1 specific antibodies was used for IHC staining. Representative RDAA positive images shown.

Table S1.

Table S1 Representative oncogenic genotype for RDAA patients. WT, wild type. +, positive. –, negative. These results were analyzed by next-generation sequencing (NGS) techniques.

	**Patient #01**	**Patient #02**	**Patient #03**	**Patient #04**	**Patient #05**	**Patient #06**	**Patient #07**	**Patient #08**	**Patient #09**
**RDAA(IHC)**	+	+	+	+	+	+	+	+	+
**EGFR E19 mutation**	WT	WT	WT	WT	WT	WT	WT	WT	WT
**EGFR E18 mutation**	WT	WT	WT	WT	WT	WT	WT	WT	WT
**EGFR E20 mutation**	WT	WT	WT	WT	WT	WT	WT	WT	WT
**EGFR E21 mutation**	WT	WT	WT	WT	WT	WT	WT	WT	WT
**MET E14 mutation**	WT	WT	WT	WT	WT	WT	WT	WT	WT
**EGFR E20 T790M**	WT	WT	WT	WT	WT	WT	WT	WT	WT
**KRAS E2 mutation**	WT	WT	WT	WT	WT	WT	WT	WT	WT
**KRAS E3 mutation**	WT	WT	WT	WT	WT	WT	WT	WT	WT
**KRAS E4 mutation**	WT	WT	WT	WT	WT	WT	WT	WT	WT
**BRAF E15 mutation**	WT	WT	WT	WT	WT	WT	WT	WT	WT
**PIK3CA E9 mutation**	WT	WT	WT	WT	WT	WT	WT	WT	WT
**PIK3CA E20 mutation**	WT	WT	WT	WT	WT	WT	WT	WT	WT
**RET re-arrangement**	–	–	–	–	–	–	–	–	–
**ALK re-arrangement**	–	–	–	–	–	–	–	–	–
**ROS1 re-arrangement**	–	–	–	–	–	–	–	–	–
**MET amplification**	–	–	–	–	–	–	–	–	–

Table S2.

Table S2 Brief information of 4 RDAA positive patients under crizotinib treatment**.**

Patient's No.	Age	Gender	First-line treatment	Response to First-line treatment	Second-line treatment	Response to second-line treatment	Disease progress-free survival	Overall survival	Other information
001	41	F	chemo	PD	crizotinib	CR	>39 months (2019.5-now)	>39 months (2019.5-now)	
002	55	F	chemo	PD	crizotinib	PR	10 months (2019.7-2020.5)	>37 months (2019.7-now)	Brain metastases (2020.5), then use Lorlatinib instead of crizotinib, PR
003	60	M	chemo	PD	crizotinib	SD	25 months (2019.7-2021.8)	>37 months (2019.7-now)	tumor progressed at 2021.8
004	61	M	chemo	PD	crizotinib	PR	35 months (2019.7-2022.6)	>37 months (2019.7-now)	tumor progressed at 2022.6

Table S3.

Table S3 Characteristics of 1173 NSCLC patients who's tissue were used for RDAA IHC staining.

Characteristics of patients		
gender（male/female）		536/637
age	Total	59.69±9.963
	male	60.11±9.758
	female	59.33±10.13
stage (7th edition TNM staging)		
	0	2
	I	724
	II	134
	III	271
	IV	42
Smoking history		
	Yes	360
	No	793
	N/A	20
ECOG		
	0	198
	1	811
	2	23
	3	5
	4	4
	5	107
	N/A	25

Table S4.

Table S4 Characteristics of EGFR/ALK/ROS1 mutation and RDAA positive rate in1173 NSCLC patients whom were mentioned in extended Table S3.

Types of gene mutation	Number of patients	Rate (%)
EGFR+	506	43.137
ROS-1+	22	1.876
ALK+	59	5.030
EGFR+/ALK+/ROS-1-	8	0.682
EGFR+/ALK-/ROS-1+	27	2.302
EGFR-/ALK+/ROS-1+	0	0.000
EGFR+/ALK+/ROS-1+	1	0.085
EGFR-/ALK-/ROS-1-	550	46.888

RDAA positive rate in patients with different genotypes

Types of gene mutation	RDAA Positive Rate（%）
EGFR+	14.625
ROS-1+	4.545
ALK+	5.085
EGFR+/ALK+/ROS-1-	0
EGFR+/ALK-/ROS-1+	0
EGFR-/ALK+/ROS-1+	0
EGFR+/ALK+/ROS-1+	0
EGFR-/ALK-/ROS-1-	18.182

Table S5.

Table S5 Detection of p-ALK Y1604, p-ALK Y1282/1283 and RNase1 antibodies in patients with EGFR-/ALK-/ROS1- genotype.

	Number of patients	Rate (%)
p-ALK（Y1604）(positive/negative)	171/379	31.090/68.909
p-ALK（Y1282/1283）(positive/negative)	194/356	35.273/64.727
RNase1(positive/negative)	471/79	85.636/14.364
patients with p-ALK（Y1604） and p-ALK（Y1282/1283） co-positive	103	18.727
patients with p-ALK（Y1604）and RNase1co-positive	161	29.273
patients with p-ALK（Y1282/1283）and RNase1 co-positive	184	33.455
patients with p-ALK（Y1604）、p-ALK（Y1282/1283）、RNase1 co-positive	100	0.182
Antibody RNase1 in patients with p-ALK（Y1604） and p-ALK（Y1282/1283） co-positive(positive/negative)	100/3	97.087/2.913
Antibody RNase1 in patients with p-ALK（Y1604）、p-ALK（Y1282/1283） single positive and p-ALK（Y1604）、p-ALK（Y1282/1283）co-negative(positive/negative)	371/76	82.998/17.002

Table S6.

Table S6 Correlation analysis of p-ALK (Y1604、Y1282/1283) and RNase1 antibodies IHC staining results.

	Pearson correlation coefficient	P value	Spearman correlation coefficient	P value	Kendall's correlation coefficient	P value
Correlation between p-ALK（Y1604） and p-ALK（Y1282/1283） expression	0.534	5.9723E-42	0.51	1.09E-37	0.351	2.0048E-16
Correlation between p-ALK （Y1282/1283） 、 p-ALK （Y1282/1283） co-expression and RNase1 expression					0.157	0.000224
