# Supplementary material for: RNase1-driven ALK-activation is an oncogenic driver and therapeutic target in non-small cell lung cancer
Source: Signal Transduct Target Ther. 2025 Apr 18;10:124. doi: 10.1038/s41392-025-02206-x (PMC12006399; doi:10.1038/s41392-025-02206-x)

Fig.1 a

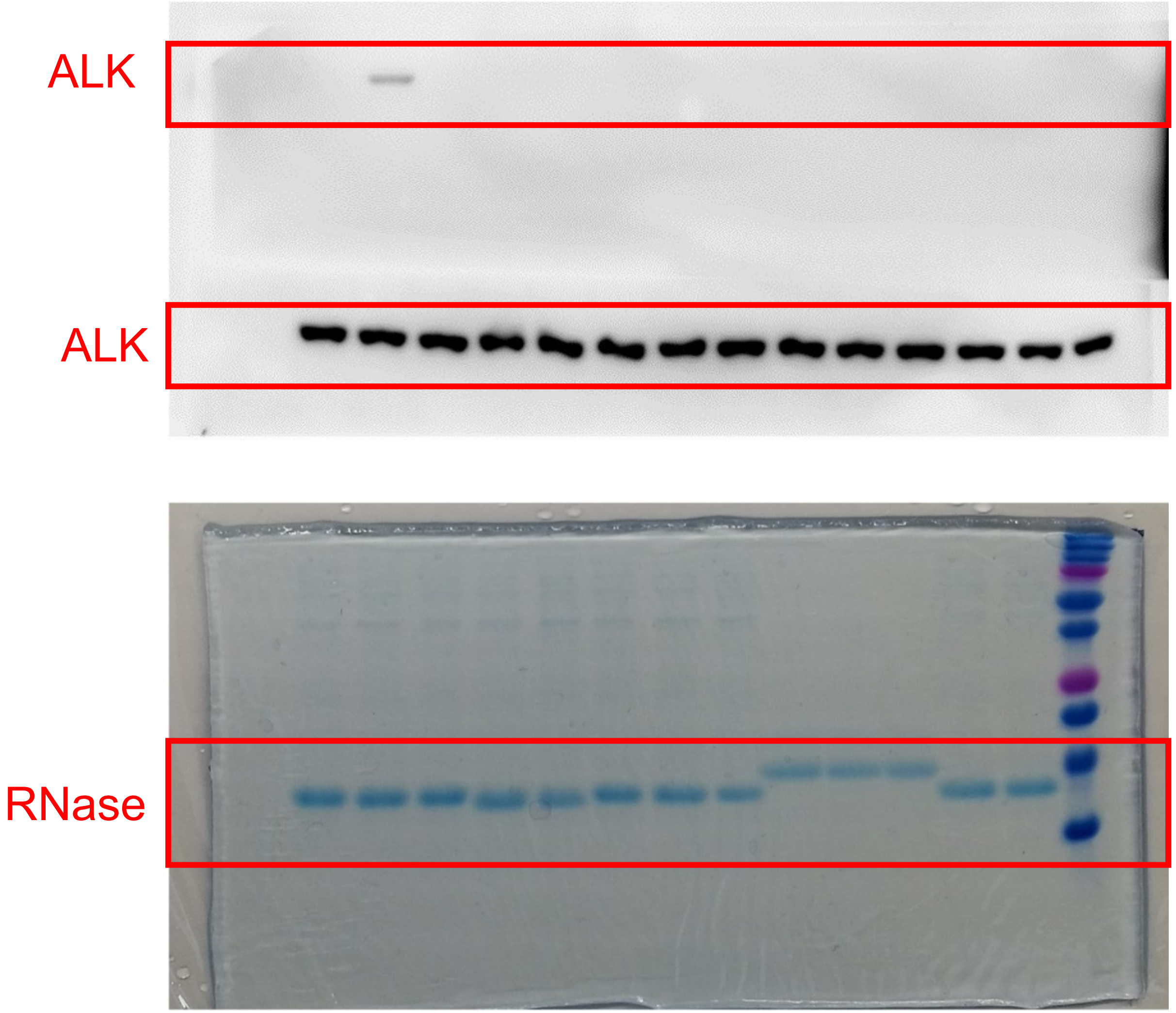

Fig.1 b

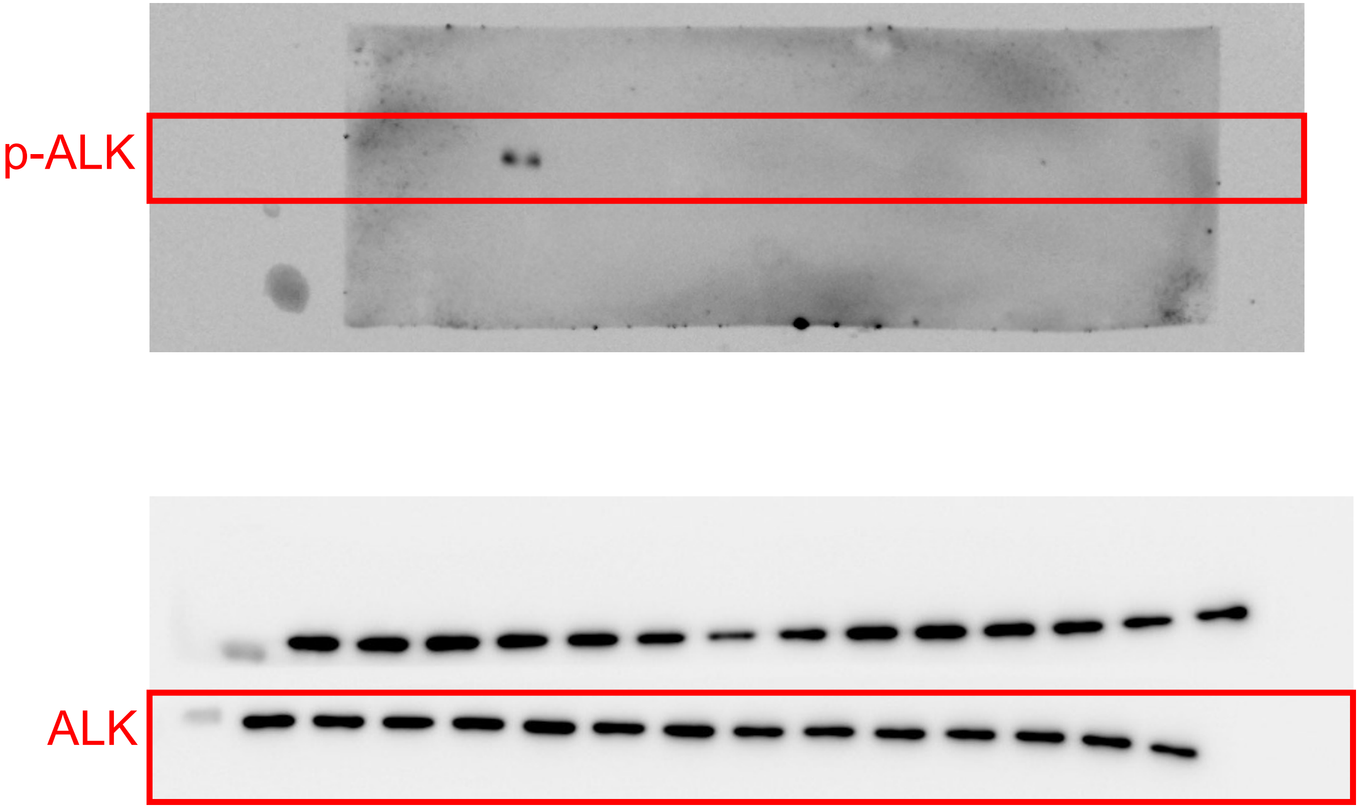

Fig.1 c

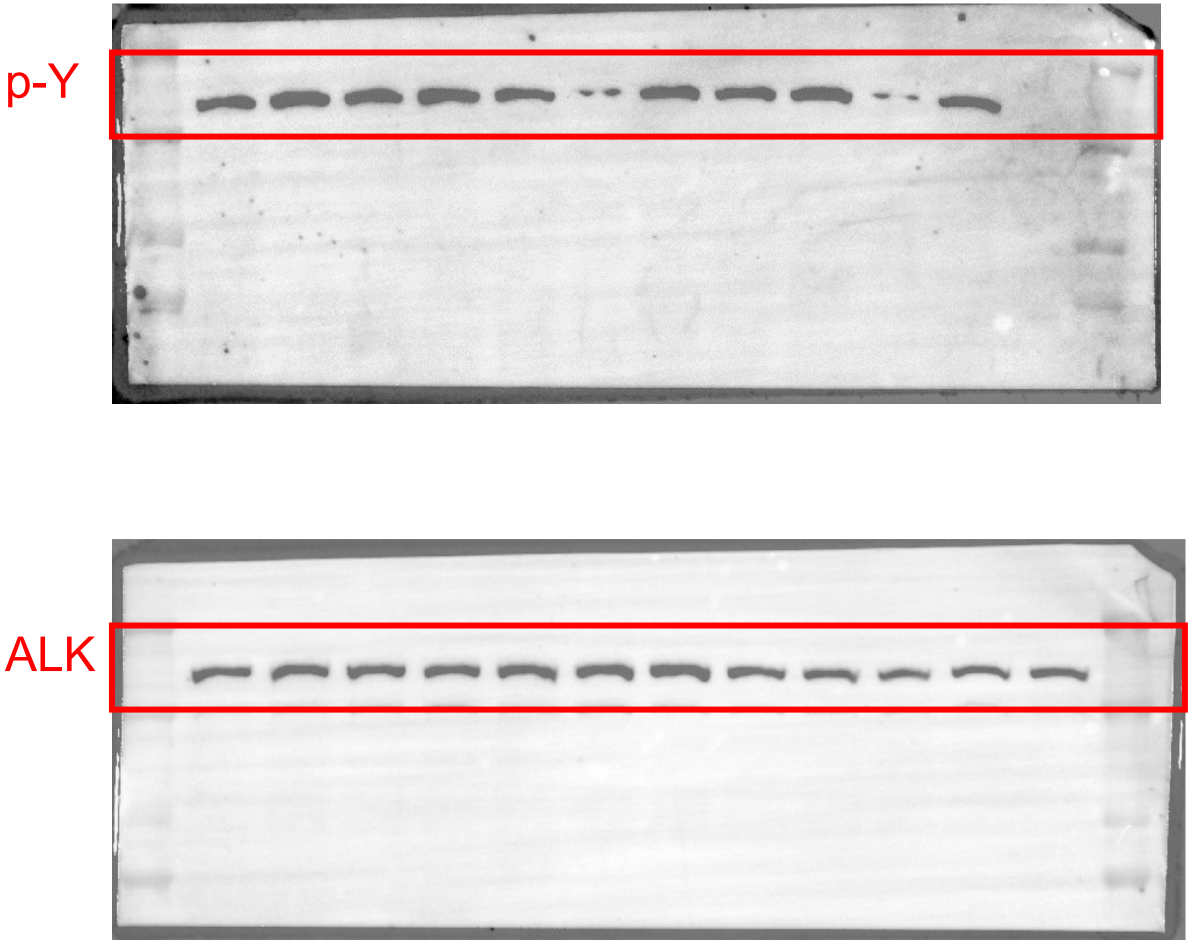

Fig.1 d

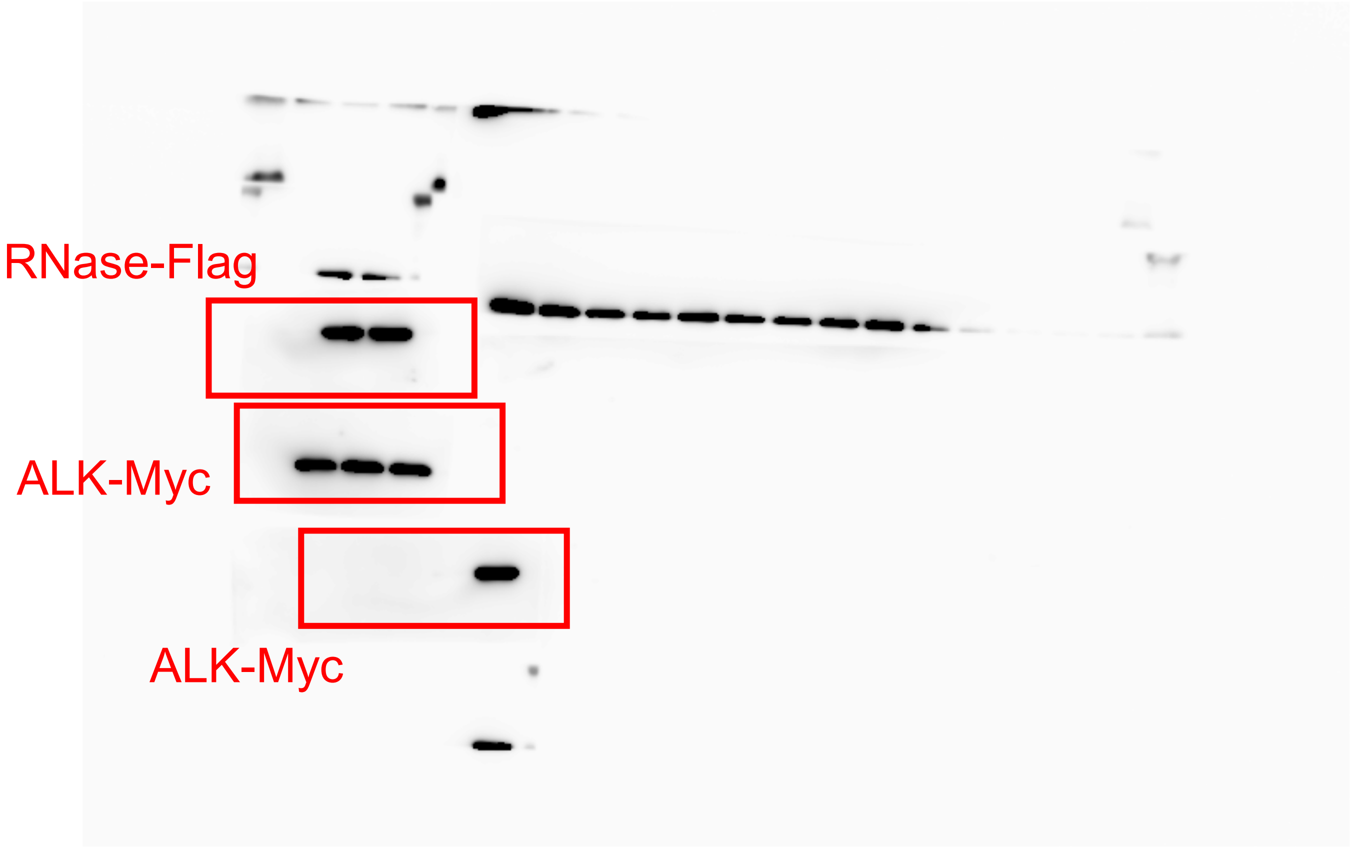

Fig.1 h

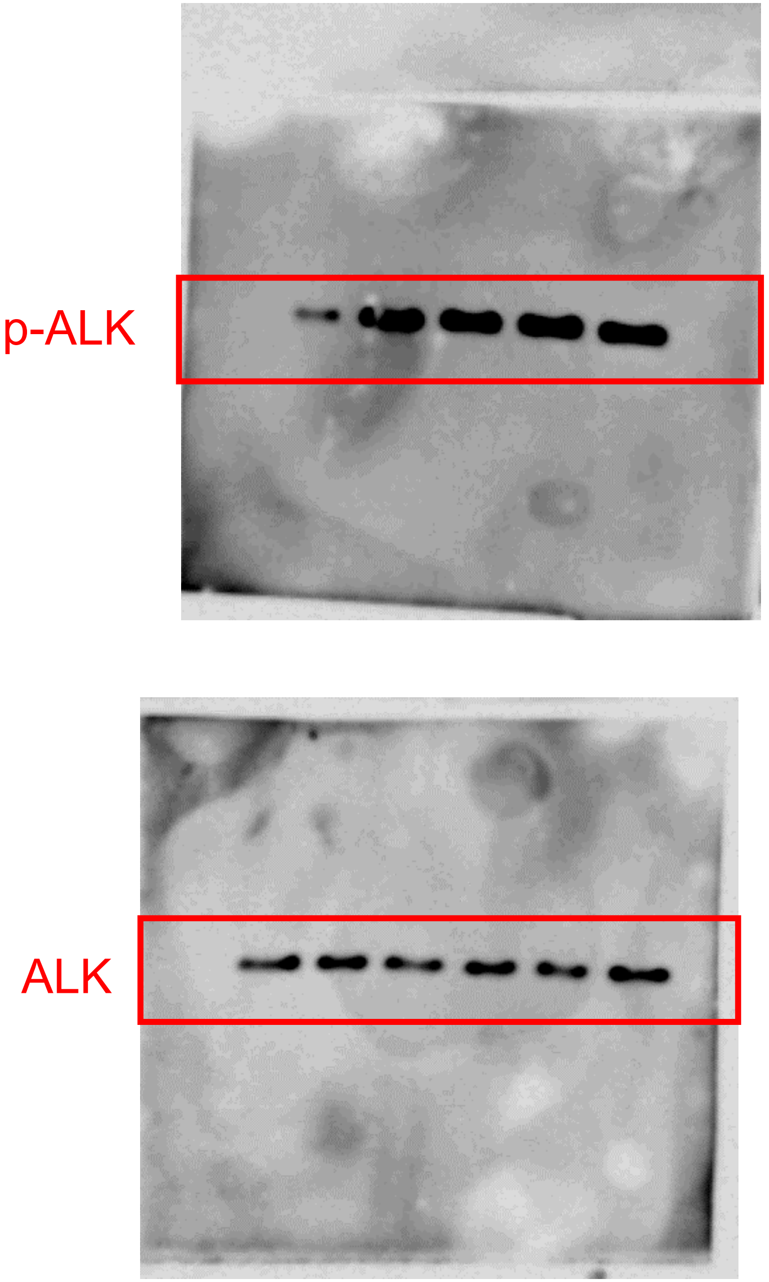

Fig.1 i

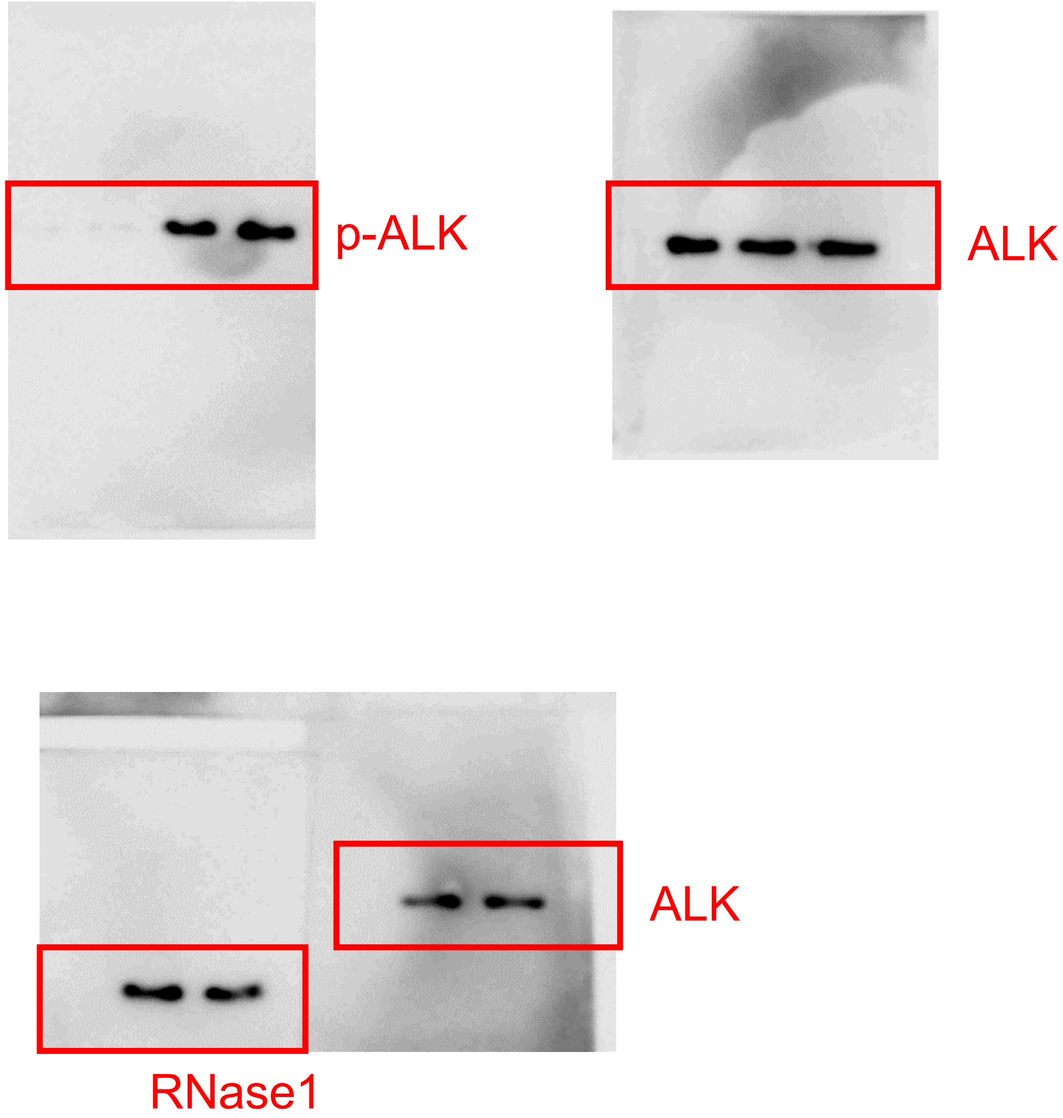

Fig.2 a

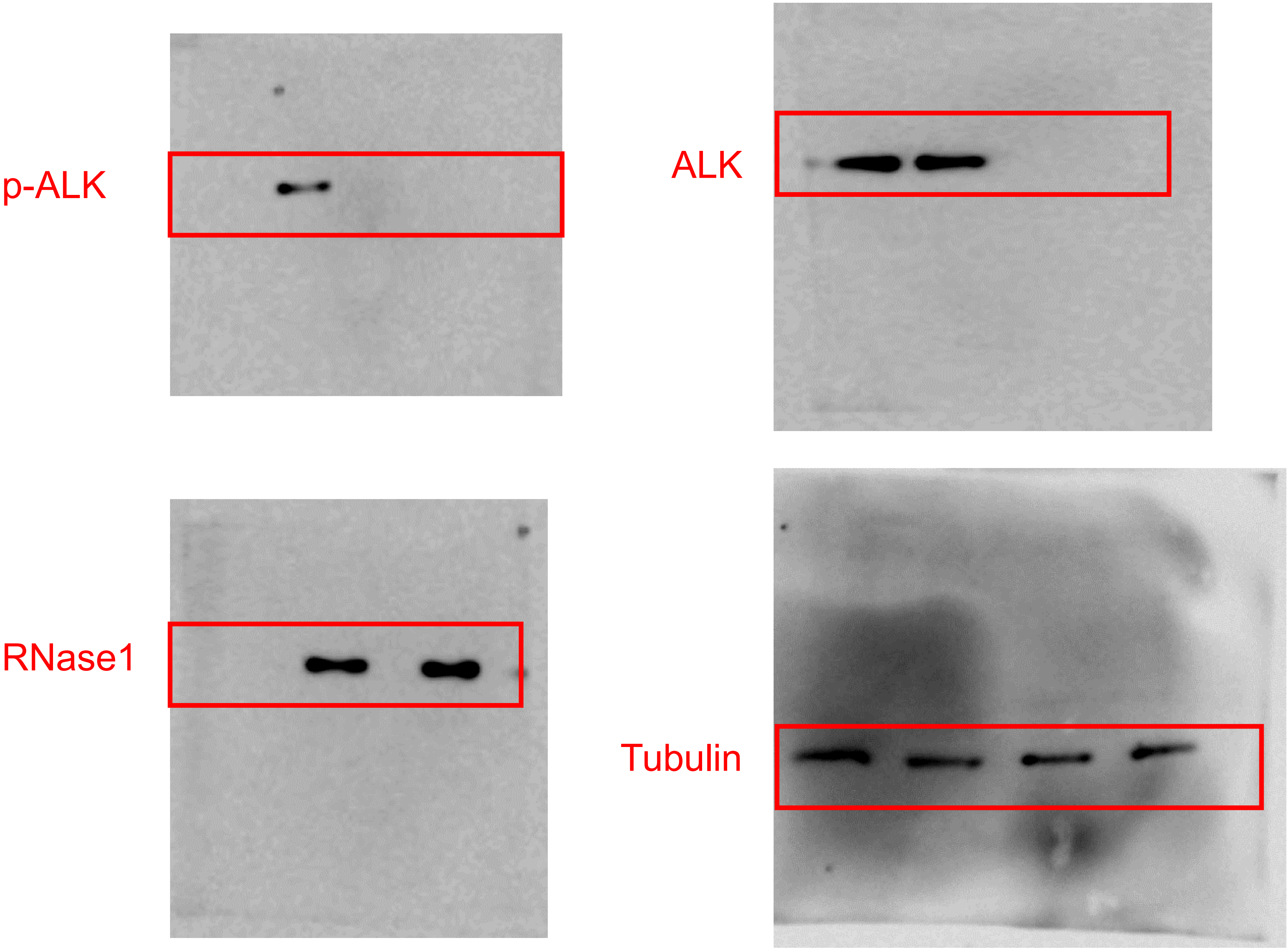

Fig.2 h

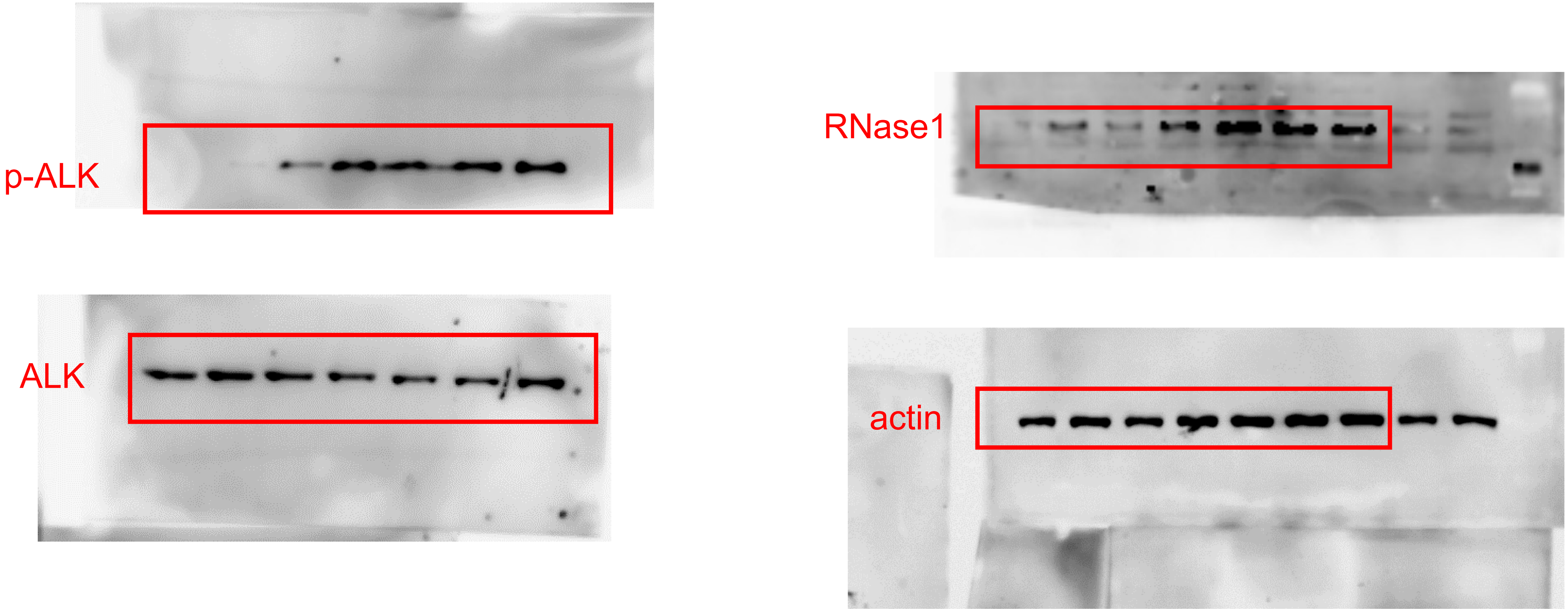

Fig.2 j

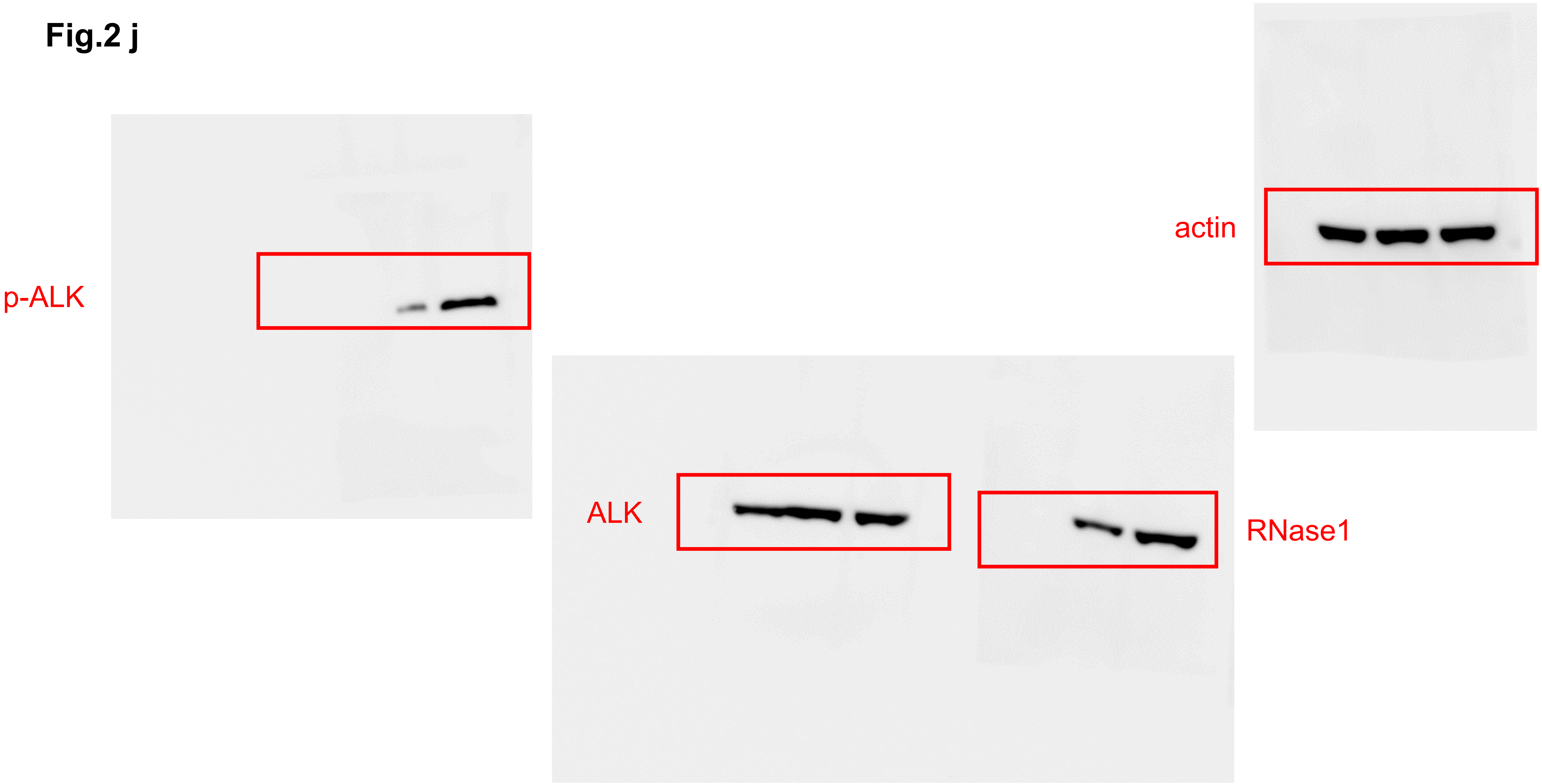

Fig.3 a

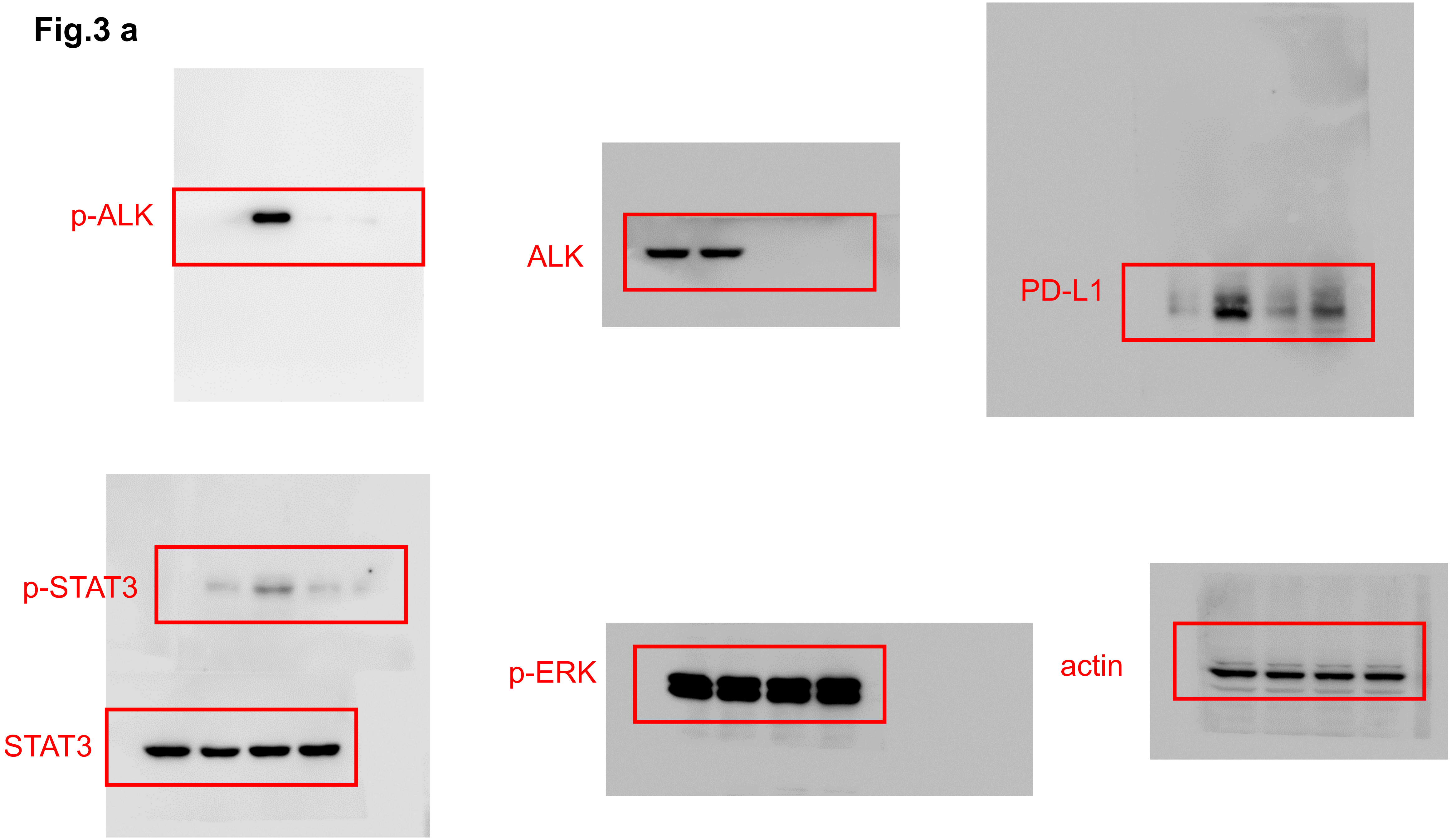

Fig.3 f

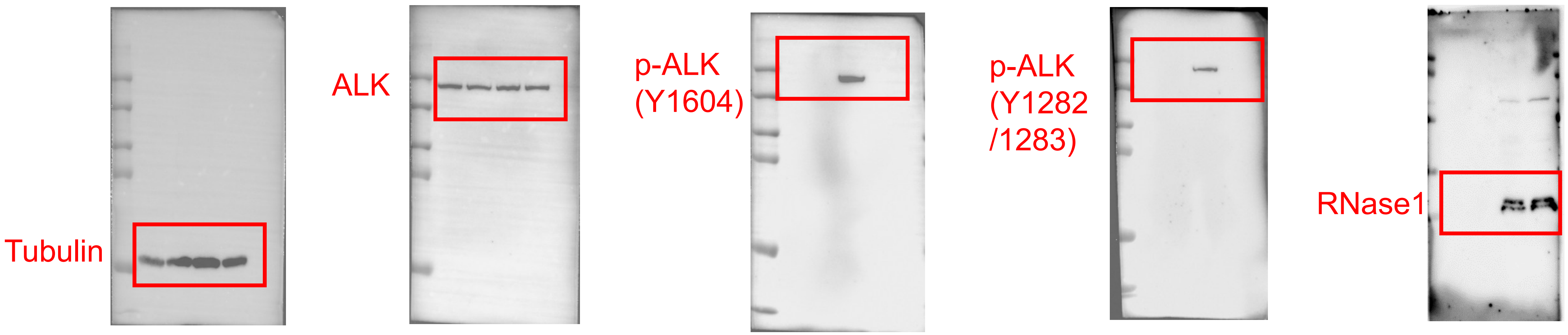

Supplement: Supplementary file 3 — Raw data-Main figure [file 41392_2025_2206_MOESM3_ESM.pdf]
