# Supplementary figures and images for: RNase1-driven ALK-activation is an oncogenic driver and therapeutic target in non-small cell lung cancer
Source: Signal Transduct Target Ther. 2025 Apr 18;10:124. doi: 10.1038/s41392-025-02206-x (PMC12006399; doi:10.1038/s41392-025-02206-x)

Fig.S1 a

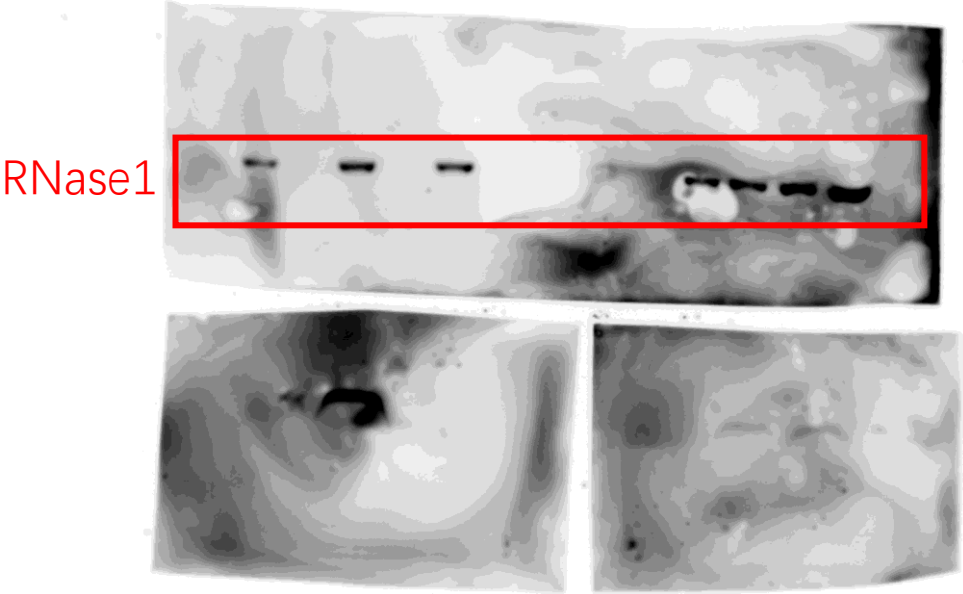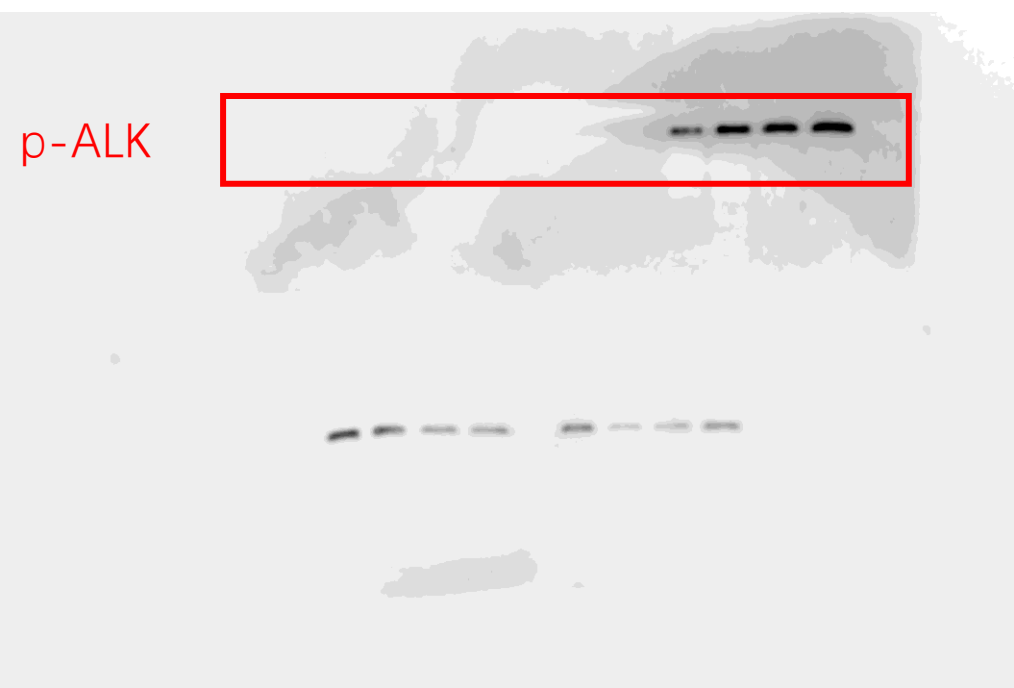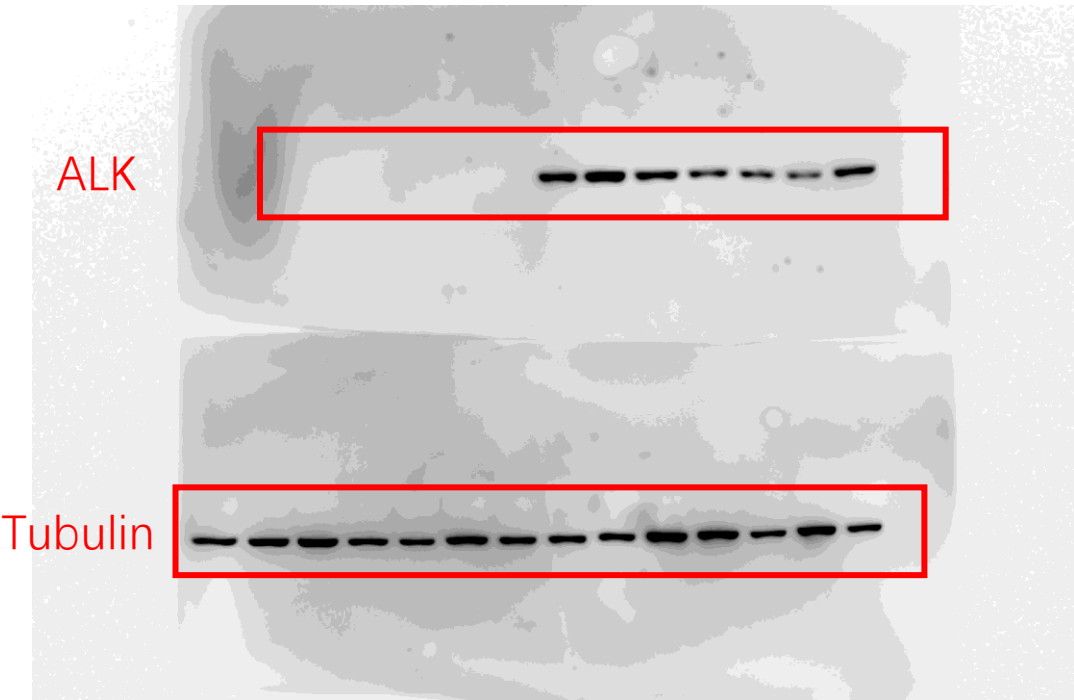

**Fig.S1 b**

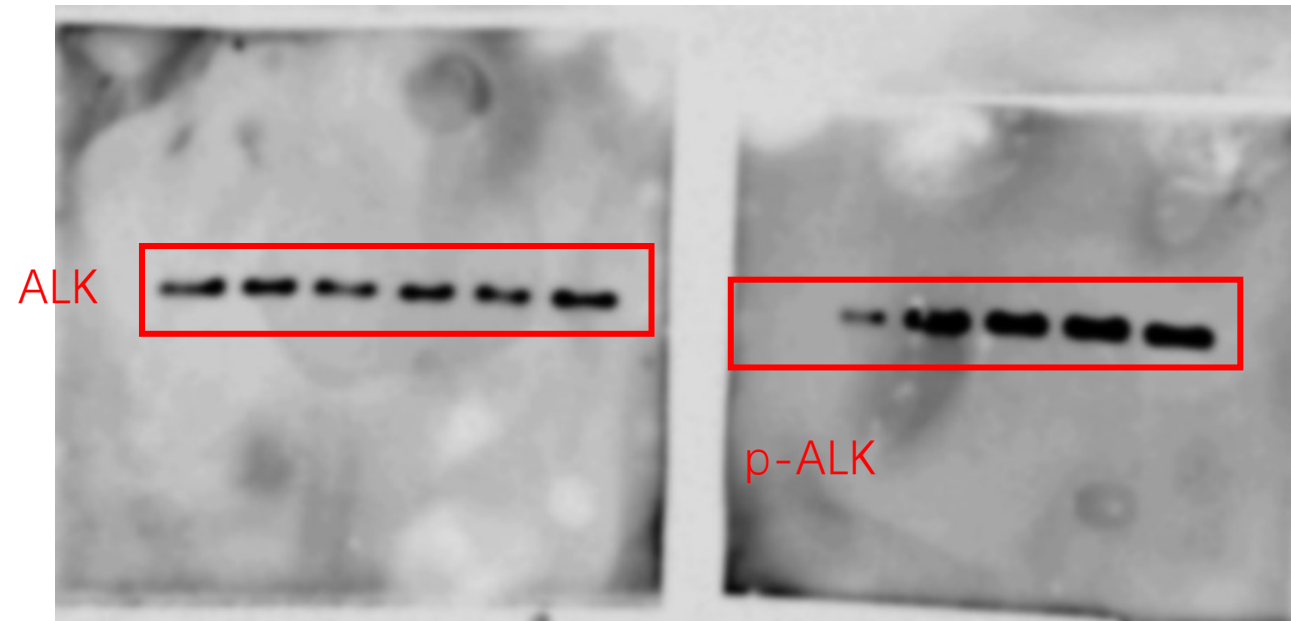

Fig.S1 c

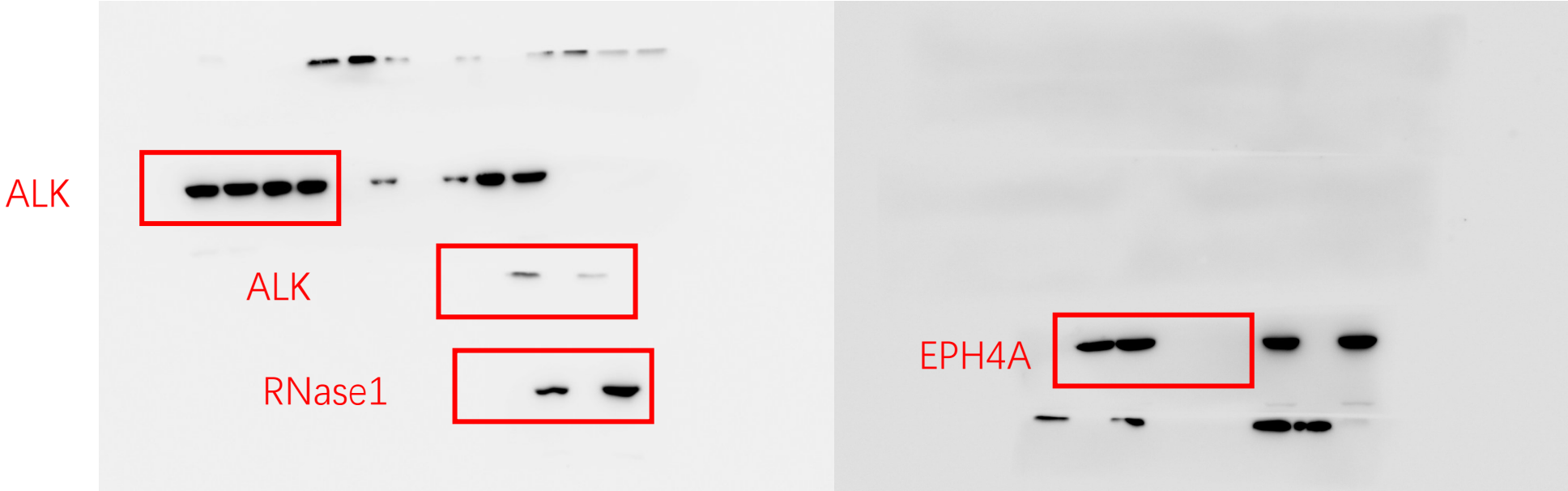

Fig.S1 d

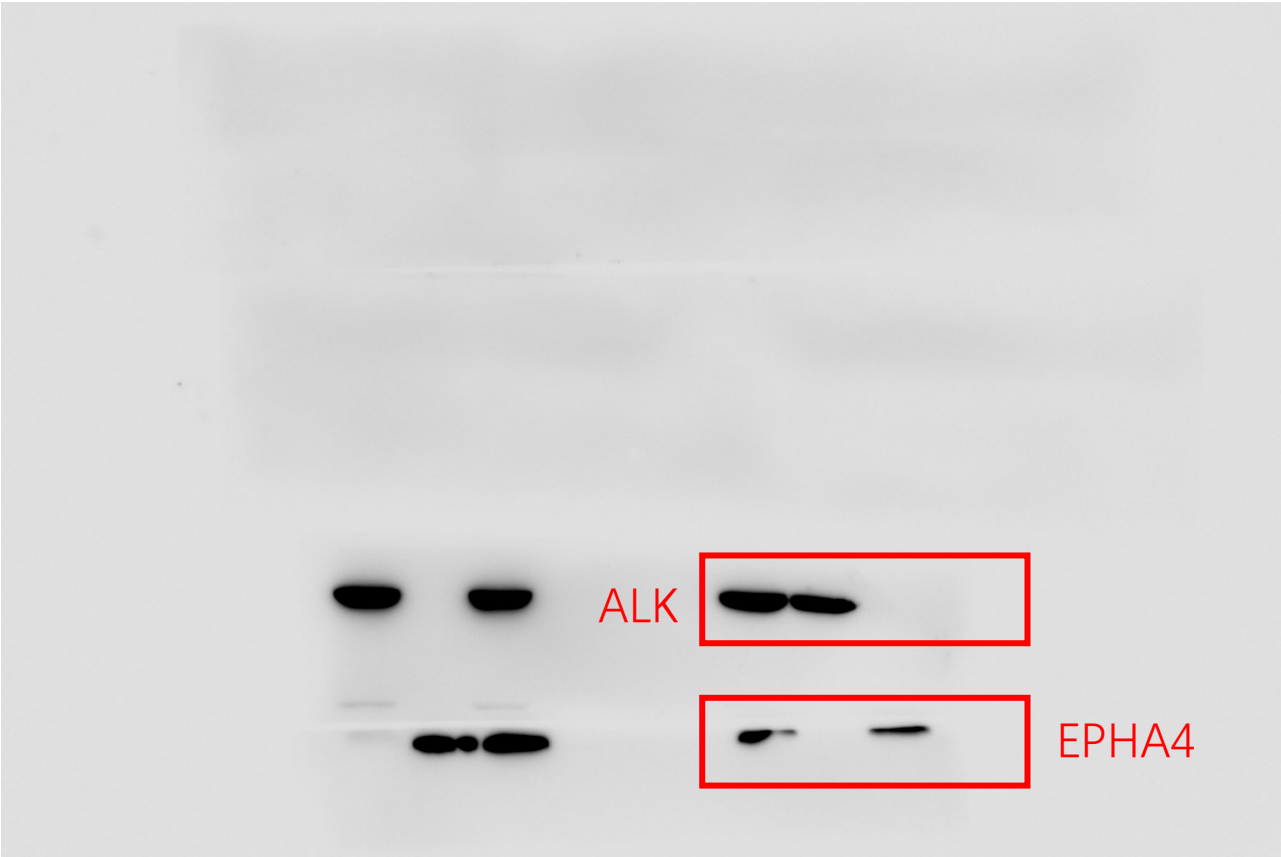

Fig.S2 e

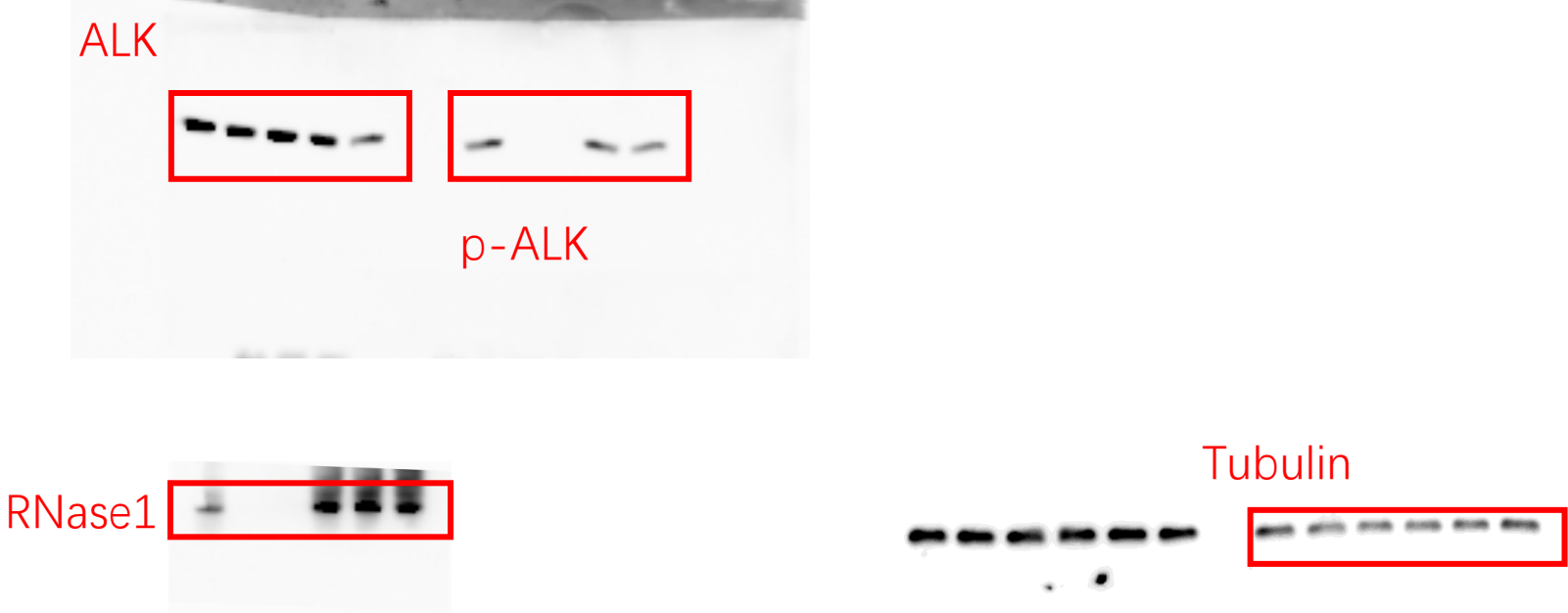

Fig.S3 b

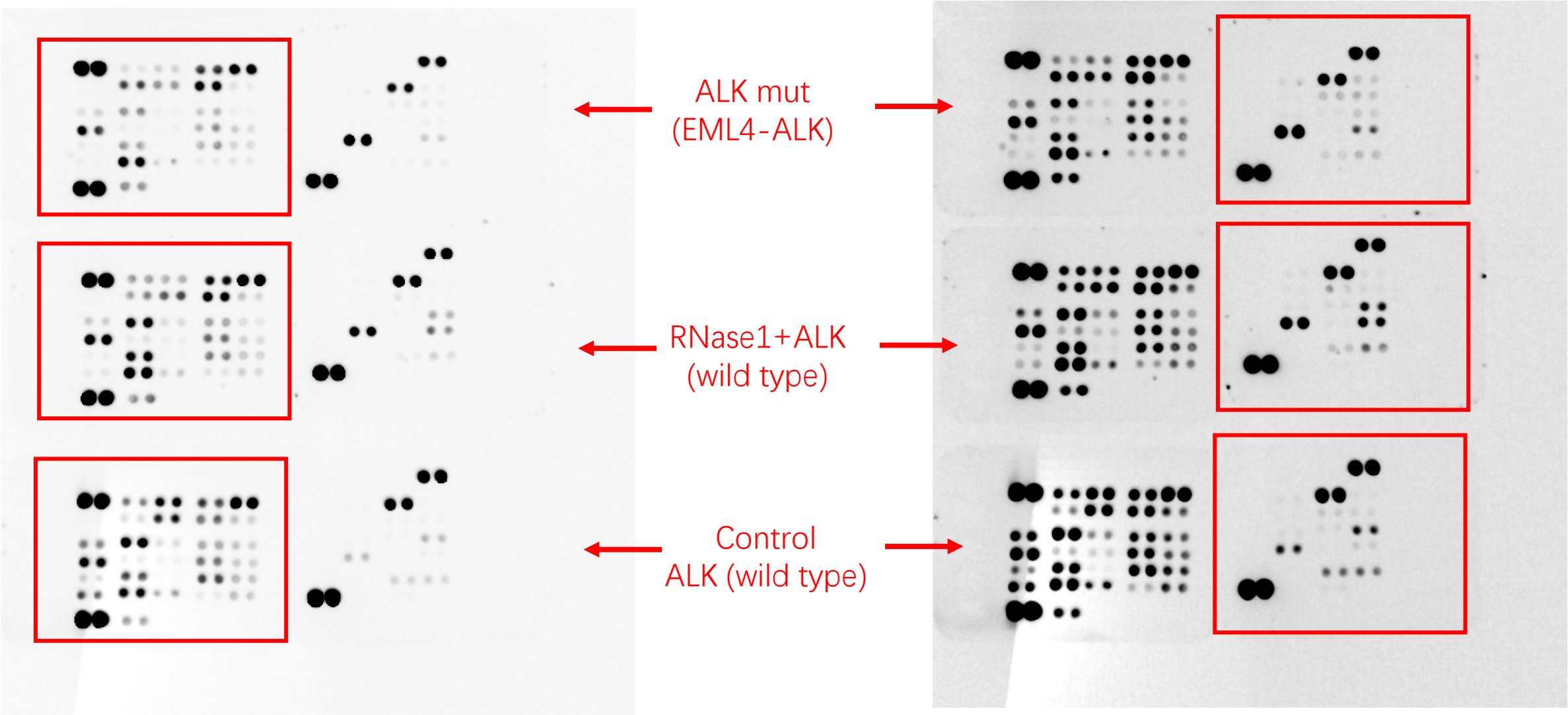

Fig.S4 c

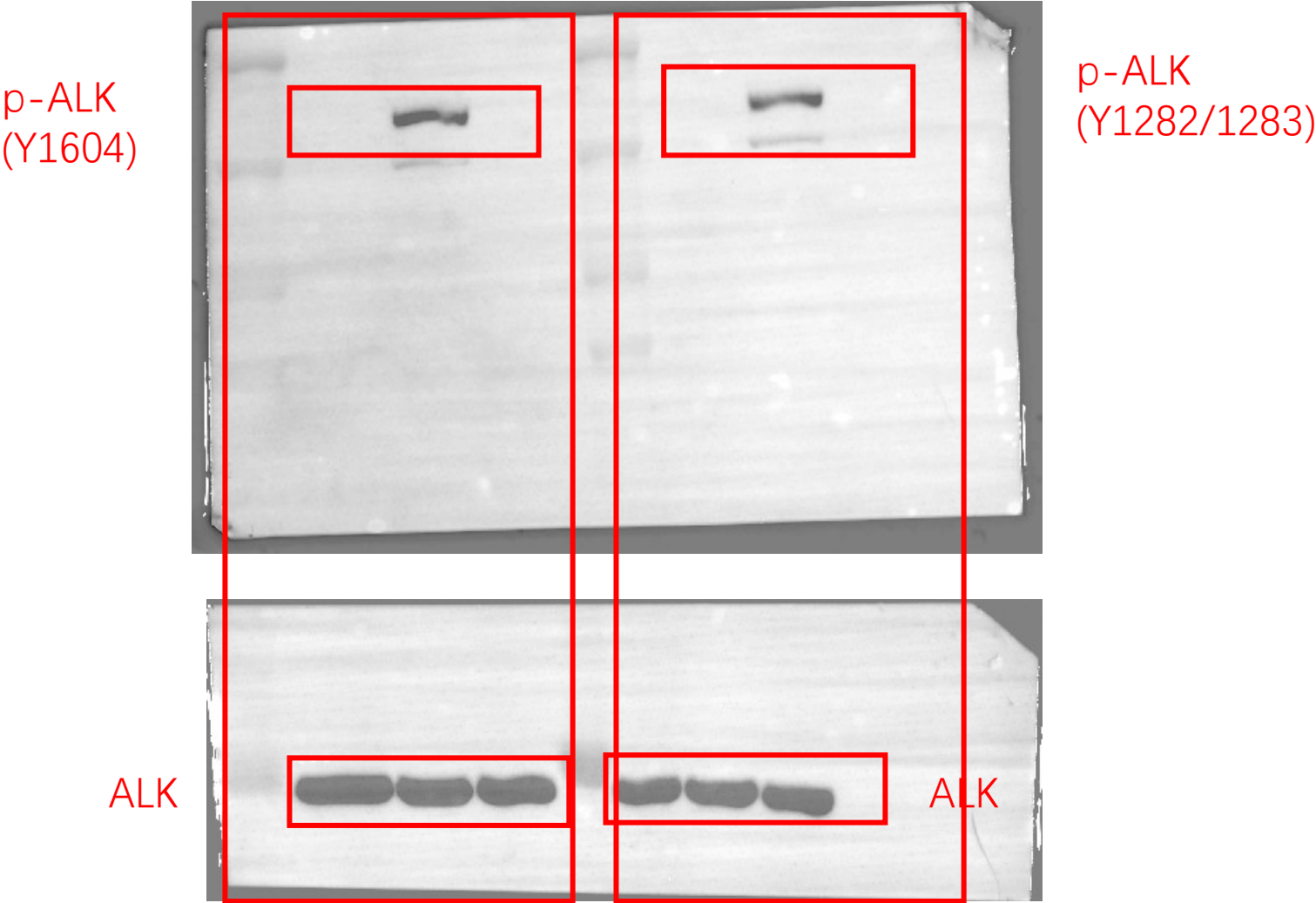

Fig.S6 b

WT ALK

EML4-ALK fusion

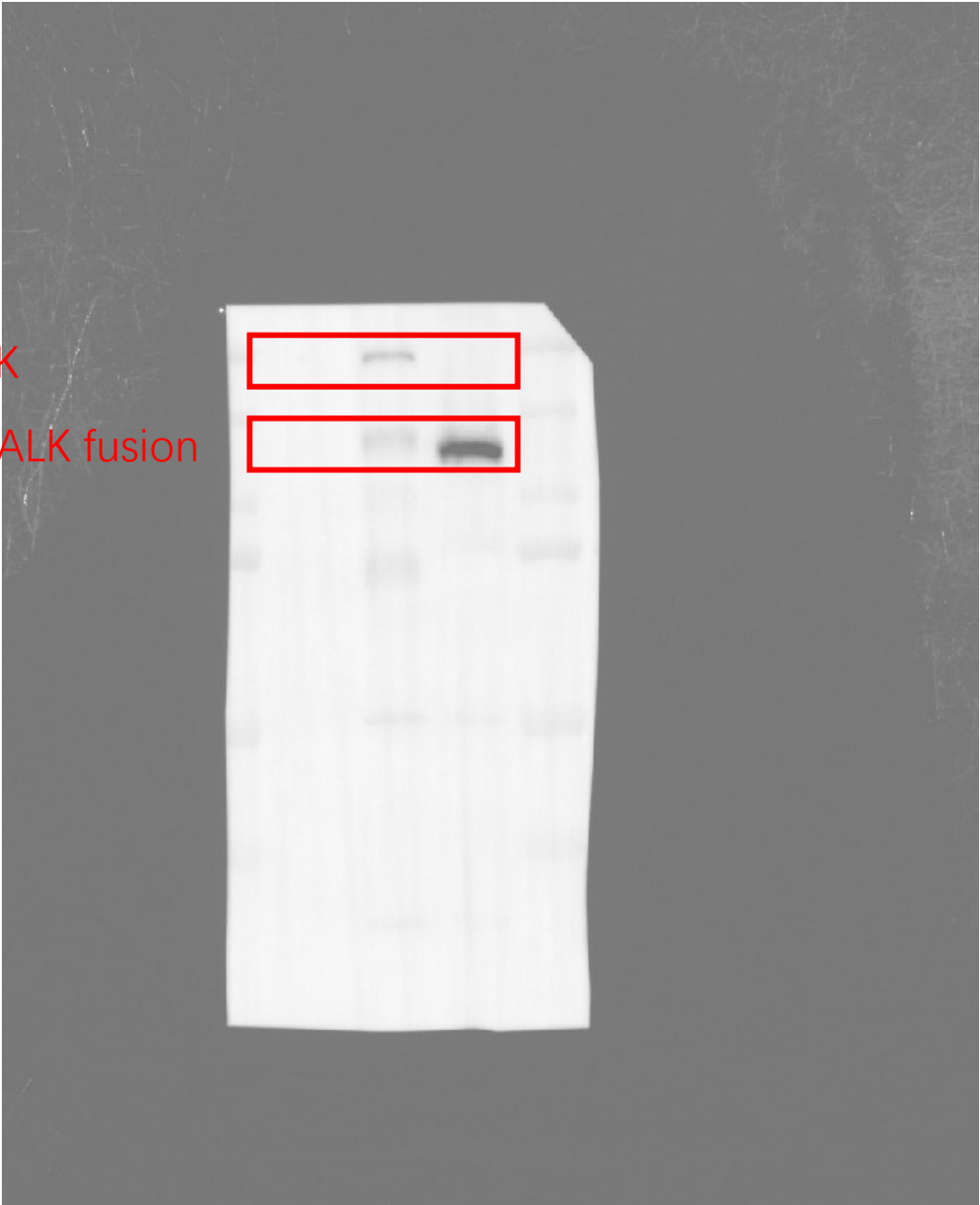

Tubulin

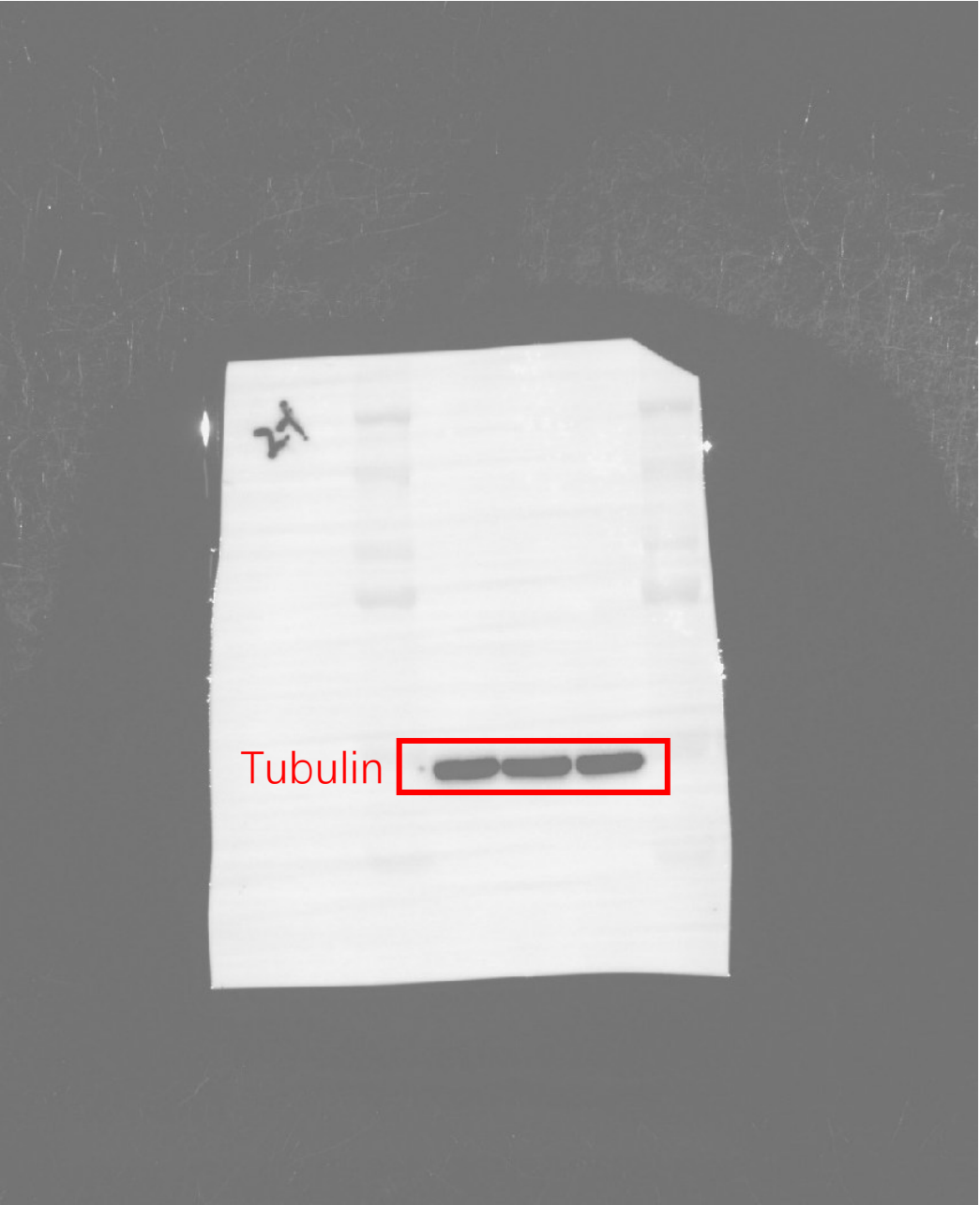

Supplement: Supplementary file 4 — Raw data-Supplementary [file 41392_2025_2206_MOESM4_ESM.pdf]
